# Physicochemical Properties and Bioreactivity of Sub‐10 μm Geogenic Particles: Comparison of Volcanic Ash and Desert Dust

**DOI:** 10.1029/2024GH001171

**Published:** 2025-01-08

**Authors:** Ines Tomašek, Julia Eychenne, David E. Damby, Adrian J. Hornby, Manolis N. Romanias, Severine Moune, Gaëlle Uzu, Federica Schiavi, Maeva Dole, Emmanuel Gardès, Mickael Laumonier, Clara Gorce, Régine Minet‐Quinard, Julie Durif, Corinne Belville, Ousmane Traoré, Loïc Blanchon, Vincent Sapin

**Affiliations:** ^1^ Laboratoire Magmas et Volcans (LMV) CNRS IRD OPGC Université Clermont Auvergne Clermont‐Ferrand France; ^2^ Institute of Genetic Reproduction and Development (iGReD) Translational Approach to Epithelial Injury and Repair Team CNRS INSERM Université Clermont Auvergne Clermont‐Ferrand France; ^3^ Istituto Nazionale di Geofisica e Vulcanologia (INGV) Osservatorio Etneo Catania Italy; ^4^ U.S. Geological Survey (USGS) Volcano Science Center Menlo Park CA USA; ^5^ Department of Earth and Atmospheric Sciences Cornell University Ithaca NY USA; ^6^ Department of Cellular and Molecular Biology School of Medicine University of Texas at Tyler Tyler TX USA; ^7^ Institut Mines‐Télécom (IMT) Nord Europe Centre for Energy and Environment Université Lille Douai France; ^8^ IRD CNRS INRAE INP‐G IGE (UMR 5001) Université Grenoble Alpes Grenoble France; ^9^ Biochemistry and Molecular Genetics Department Centre Hospitalier Universitaire (CHU) Clermont‐Ferrand Clermont‐Ferrand France; ^10^ Infection Control Department Centre Hospitalier Universitaire (CHU) Clermont‐Ferrand Clermont‐Ferrand France; ^11^ Laboratoire Microorganismes: Génome Environnement (LMGE) UMR CNRS Université Clermont Auvergne Clermont‐Ferrand France

**Keywords:** desert dust, volcanic ash, particulate matter, respiratory hazard, physicochemical properties, bioreactivity

## Abstract

Exposure to ambient particulate matter (PM) with an aerodynamic diameter of <10 μm (PM_10_) is a well‐established health hazard. There is increasing evidence that geogenic (Earth‐derived) particles can induce adverse biological effects upon inhalation, though there is high variability in particle bioreactivity that is associated with particle source and physicochemical properties. In this study, we investigated physicochemical properties and biological reactivity of volcanic ash from the April 2021 eruption of La Soufrière volcano, St. Vincent, and two desert dust samples: a standardized test dust from Arizona and an aeolian Gobi Desert dust sampled in China. We determined particle size, morphology, mineralogy, surface texture and chemistry in sub‐10 μm material to investigate associations between particle physicochemical properties and observed bioreactivity. We assessed cellular responses (cytotoxic and pro‐inflammatory effects) to acute particle exposures (24 hr) in monocultures at the air‐liquid interface using two types of cells of the human airways: BEAS‐2B bronchial epithelial cells and A549 alveolar type II epithelial cells. In acellular assays, we also assessed particle oxidative potential and the presence of microorganisms. The results showed that volcanic ash and desert dust exhibit intrinsically different particle morphology, surface textures and chemistry, and variable mineralogical content. We found that Gobi Desert dust is more bioreactive than freshly erupted volcanic ash and Arizona test dust, which is possibly linked to the presence of microorganisms (bacteria) and/or nanoscale elongated silicate minerals (potentially clay such as illite or vermiculite) on particle surfaces.

## Key Points

- There are intrinsic differences in physicochemical properties between inhalable (sub‐10 μm) freshly erupted volcanic ash and desert dust samples
- Gobi Desert dust appears to be more bioreactive in lung airway models than volcanic ash from St. Vincent and a standardized test dust from the Arizona Desert
- Our results confirm that analyzing only one particle property is not sufficient to predict particle bioreactivity for mixed‐mineral dusts

## 1 Introduction

Exposure to ambient particulate matter (PM) with an aerodynamic diameter of ≤10 μm (PM_10_) is a well‐established health hazard that is associated with respiratory and cardiovascular disease exacerbation, hospitalization, and mortality (WHO, 2021). Although air pollution and its health effects are usually associated with particles from human activities, such as fossil fuel combustion (from traffic and industry), vast quantities of PM_10_ are produced by natural events, such as dust storms and volcanic eruptions (Kelly & Fussell, 2020), and are mostly made up of minerals and glass. Such geogenic (earth‐derived) particles, often referred to as mineral dust in the literature, can be an important constituent of both urban and non‐urban PM_10_ that can affect air quality over wide areas and extended periods (Goudie, 2014; Querol et al., 2019; Williams et al., 2017). Still, compared to PM_10_ from anthropogenic sources, less is known about the potential health effects of diverse geogenic PM_10_ (Williams et al., 2017).

One of the main contributors to geogenic PM_10_ are dust storms, events in which large quantities of dust (i.e., particles constituting the uppermost layer of soil) from sparsely vegetated and dry land surfaces in semiarid and arid regions are lofted into the atmosphere by strong winds (Adebiyi et al., 2023). The emission of such particles, hereafter referred to as mineral dust, are increasing due to desertification and changes in land use (Langmann, 2013). Globally, the major source regions for mineral dust emission are deserts, principally the Sahara Desert in Northern Africa (often referred to as “African dust”) and the Gobi Desert in Eastern Asia (“Asian dust”) (Ginoux et al., 2012). These regions are active throughout the year, though the frequency and intensity of dust emissions are subject to seasonal variability (Querol et al., 2019).

Although volcanic eruptions are usually seen as sporadic and unpredictable events, at any given time worldwide there are an average of 20 volcanoes erupting (Siebert et al., 2015), introducing volcanic pollutants (ash particles, gases, and sulfate aerosol) into the atmosphere (Stewart et al., 2022). Volcanic ash (defined as grains <2 mm in diameter) has a heterogeneous composition constituted of fragments bearing amorphous (glass) and crystalline (mineral) material, and it is one of the most widespread volcanic hazards (Jenkins et al., 2015). Ash is generated by magma fragmentation during explosive volcanic events, but also through secondary processes that lead to particle size reduction (Andronico & Del Carlo, 2016; Paredes‐Mariño et al., 2022), and it can contain substantial amounts of PM_10_ (Horwell, 2007). Volcanic ash emission is not restricted to the duration of a volcanic eruption. Ash can also be remobilized into the atmosphere from deposits accumulated on land by the wind and human activity (Fries et al., 2023; Jarvis et al., 2020; Wilson et al., 2011), similar to what is observed for mineral dust, and particularly from regions with volcanic deserts such as Patagonia, Alaska, and Iceland (Arnalds et al., 2016; Forte et al., 2018; Hadley et al., 2004).

While geogenic particles like desert dust and volcanic ash have common main constituents (silicate minerals), these particles are physically and chemically heterogeneous, and their properties can exhibit high spatial and temporal variability (Querol et al., 2019; Stewart et al., 2022). Importantly, even before their release into the atmosphere, the history of mineral dust and volcanic ash particles is rather different (Langmann, 2013). Airborne mineral dust derived from desert environments is typically a mixture of minerals and organic matter that can originate from a specific soil or geological deposit, or multiple local and distal sources (Keil et al., 2016). These particles are subjected to natural weathering and geomorphological processes through time (i.e., extensive mechanical and biogeochemical weathering), which affects their physicochemical properties. By comparison, volcanic ash is considered a relatively fresh material whose chemical and mineralogical composition is dictated by the magma from which it is generated (Langmann, 2013).

Further, particles are subjected to various physicochemical processes during the transport and deposition from the atmosphere that can change their surface composition and reactivity. Before cooling to ambient temperatures, freshly erupted volcanic ash also undergoes processing within the eruption plume where it reacts with volcanic gases and aerosols (Ayris & Delmelle, 2012). These reactions lead to the scavenging of volatiles (such as sulfur and halogen gases and metals) and the formation of salts, which commonly consist of various sulfates and halides on the ash surfaces (Delmelle et al., 2007; Stewart et al., 2020). Aged (weathered) or remobilized ash from volcanic deposits can exhibit properties that are different to primary material (Fries et al., 2023; Horwell et al., 2010), so it can be considered a type of mineral dust (i.e., volcanic dust).

Upon their emission and depending on meteorological factors, geogenic particles can be transported near surface levels or be lofted to high altitudes and undergo long‐range transport to be distributed thousands of kilometers away from the source (Babu et al., 2022; Durant et al., 2012; Ginoux et al., 2012). Volcanic ash and mineral dust particles, in general, can travel with and/or interact with gases and toxic chemicals from anthropogenic emissions when moving across urban regions. This can enrich particles in harmful organic pollutants, sulfates, nitrates and metals (Goudie, 2014; Querol et al., 2019; Tomašek, Damby, Andronico, et al., 2021). Moreover, dust storms can transport biological materials such as bacteria, pollen and spores, fungi and viruses, inherited from the soil biota or picked‐up in the ambient air upon interactions with bioaerosols, which are capable of surviving long‐range transport (Dawrs et al., 2024; Goudie, 2014; Griffin, 2007; Smith et al., 2013). The presence of chemicals and pathogens on geogenic particles can increase their bioreactivity and enhance the toxicity of existing ambient aerosols in urban environments (Fussell & Kelly, 2021).

Other factors that can influence the potential respiratory toxicity of particles include their morphology, mineralogy, chemical composition and surface characteristics, including surface area and the capacity to generate deleterious free radicals and/or deplete the antioxidants in the human body (i.e., oxidative potential (OP)) (Derbyshire, 2007; Fubini, 1997; Plumlee et al., 2006). These properties are well‐researched for mineral‐bearing particles in the occupational setting. It has been established that certain mineral groups, such as crystalline silica and asbestos, because of their surface functionalities and morphologies, respectively, have pro‐inflammatory properties and can induce severe lung conditions, like pneumoconiosis and cancer (Mossman & Churg, 1998; Straif et al., 2009). Much less is known about the characteristics and effects of geogenic particles from environmental exposures. A major complicating factor is the high variability in reported particle bioreactivities, related to their heterogeneous physicochemical properties (Kelly & Fussell, 2012). With regard to desert dust bioreactivity, toxicological studies demonstrated that they can induce oxidative stress and inflammatory signaling (Bredeck, Dobner, et al., 2023; Fussell & Kelly, 2021). Generally, volcanic ash is found to have limited cytotoxic potential, but can provoke inflammatory reactions in animal models and pro‐inflammatory reactions in cell culture models (Damby et al., 2016; Eychenne et al., 2022; Lee & Richards, 2004).

Concerning documented health effects in exposed populations, the occurrence of respiratory symptoms and ailments varies among studies, but most studies report that inhalation of geogenic particles is associated with adverse effects (Williams et al., 2017). It has been reported that exposure to desert dust increases respiratory symptoms and is associated with some acute lung disorders and infections (e.g., “Desert Lung Syndrome” or “Desert Storm pneumonitis”) (Morman & Plumlee, 2013; Taylor et al., 2013). Similarly, acute exposure to volcanic ash was found to trigger acute respiratory issues (e.g., bronchitis) and to exacerbate symptoms of pre‐existing lung conditions (e.g., asthma), but there are not sufficient studies on chronic exposures nor evidence they can trigger silicosis or lung cancer (Baxter & Horwell, 2015; Stewart et al., 2022). Even though there is accumulating evidence of adverse effects associated with exposures to geogenic PM, there are insufficient data to identify differences in the health effects of particles with different physicochemical characteristics nor sufficient studies comparing the different particle types (Stanek et al., 2011; Zosky et al., 2014).

In order to contribute to this limited knowledge, we investigated physicochemical properties and in vitro biological reactivity of naturally abundant geogenic particle types including fresh (unweathered) volcanic ash (from the April 2021 eruption of La Soufrière volcano, St. Vincent) and two desert dust samples: a standardized test dust from Arizona and an environmentally sampled Gobi Desert dust. We isolated sub‐10 μm material from bulk samples using an in‐house separation set‐up and determined particle size, morphology, mineralogy, surface texture and chemistry to establish if there is an association of particle physicochemical properties with in vitro bioreactivity or particle OP, which is generally implicated in inflammatory responses to particles. We also determined the presence of microorganisms, which can generate an immune response. To study the bioreactivity of desert dust and volcanic ash in the lungs, we focused on epithelial cell models, given that the respiratory epithelium is a first barrier against inhaled pollutants and plays a critical role in the defensive response to pollutants through the production of pro‐inflammatory mediators by the epithelial cells (Parker & Prince, 2011). We used epithelial cells ‐ human alveolar (A549) and bronchial (BEAS‐2B) cells ‐ that represent the main pulmonary cell types and are the most used in inhalation toxicity studies (Hiemstra et al., 2018). A549 cells are carcinoma cells representing alveolar type II cells, while BEAS‐2B cells are immortalized cells from normal human bronchial epithelia and resemble airway basal epithelial cells. The cells were cultured at the air–liquid interface (ALI; with cell culture medium only at the basolateral side of a microporous growth membrane) which is an established, highly relevant model for inhalation toxicity studies aiming to better mimic the physiology of the respiratory system (Lacroix et al., 2018). In monocultures of the two cell types, we assessed cytotoxicity and a pro‐inflammatory response (release of cytokines IL‐6, IL‐8, IL‐1β and TNF‐α) to acute particle exposures (24 hr). We compared the cell responses to the particle properties to identify potential drivers of the bioreactivity of the geogenic particle samples studied.

## 2 Materials and Methods

### 2.1 Dust Sample Sourcing and Preparation

Arizona test dust (ATD; nominal 0–10 μm grade; *Powder Technology Inc.*, USA) is a commercially available standardized material. According to the manufacturer's information, it is produced by jet milling of naturally occurring sand collected from Salt River Valley, Arizona, USA. ATD is commonly used as representative of natural mineral dust in environmental and atmospheric chemistry studies (e.g., Romanias et al., 2016; Joshi et al., 2017). Gobi Desert dust (GDD) is a natural aeolian deposit collected in Ningxia Province, China (N 36°29′14.39 “E 107°28′30.75”). After collection, the sample was dry‐sieved to a <100 μm fraction (Urupina et al., 2021). Volcanic ash was sourced pristine (not rained on) from an ashfall of the April 2021 eruption of La Soufrière volcano, St. Vincent. The sample was collected on 10 April 2021, in Kingstown, the capital of St. Vincent and the Grenadines, located 20 km from the volcanic vent. This volcanic ash sample, hereafter referred to as SVA, is from the explosive phases of the eruption that occurred between 9 and 10 April.

A respirable fraction of particles (PM_10_) was isolated from the GDD and the SVA samples using a custom‐made aerodynamic separation apparatus at Laboratoire Magmas et Volcans (LMV; Clermont‐Ferrand, France). Each bulk original sample is introduced in an air elutriation column (6 cm in diameter), where gentle shaking and air flow (6 L/min) ensure the suspension of particles with sizes approximately <25 μm. The particle suspension is sucked into a cyclone (SCCA^®^, by *BGI, Mesa Labs*), in which particles approximately >8 μm are trapped and the particles <8 μm are collected upstream on a filter (*Whatman* Nuclepore Track‐Etched membranes in polycarbonate). The ATD sample did not need separation, representing already a respirable fraction. For the physicochemical analyses, it was dispersed on the same polycarbonate membranes as GDD and SVA by simple elutriation and collection, without cyclone separation.

### 2.2 Sample Characterization

#### 2.2.1 Particle Size Analysis

The particle size distribution of the ATD sample and the isolated respirable fractions from the GDD and SVA samples were assessed by laser diffraction analyses in water on a Malvern Mastersizer 3000 at the Chemistry Institute of University Clermont Auvergne (ICCF; Clermont‐Ferrand, France). Measurements were repeated twice without and with ultra‐sonication. Each individual measurement lasted for 60 s. An absorption coefficient of 0.1 and a refractive index of 1.63 was used to derive particle size distributions in volume % and number %.

#### 2.2.2 Bulk Chemical Composition

The chemical composition of bulk samples was determined by inductively coupled plasma ‐ optical emission spectrometry (ICP‐OES) on the Agilent 5800 instrument at LMV. Aliquots of 100 mg of the bulk (not size separated) samples were mixed with 300 mg of a LiBO_2_ solution in a porcelain dish, transferred to a graphite crucible machined from 25 mm diameter rods and fused for 5 min at about 1100°C in an induction furnace (2 kW). The melt was poured into a disposable polystyrene beaker containing 50 mL of 1 M HNO_3_ and stirred by a magnetic bar. After the complete dissolution of the shattered quenched melt droplets (about 15 min), the solution was passed through a filter paper (*Whatman*, N° 40, 110 mm diameter) to remove graphite particles. The final volume was raised to 200 mL with 1 M HNO_3_.

The instrument was calibrated using reference materials GH (for Si, Na, K) and BR (for the other elements), both from Centre Pétrographique et Géochimique (CRPG, Nancy, France). Prepared in the same way as the unknown samples, they provide the high values of the calibration lines, while a pure LiBO_2_ solution (300 mg in 200 mL of 1 M HNO_3_) was used as the zero value in every case. Data have been recalculated to include loss on ignition, which was determined by weighing the bulk sample before and after 1 hr of calcination at 110°C and after 1 hr at 1000°C. Uncertainty on the analyses was monitored by replication of international rock standards (DR‐N and BHVO‐1) every 10 measurements. The uncertainties for each major element were calculated as 2 times the standard deviation of the measurement errors (differences between the reference value and the measured value).

#### 2.2.3 Particle Imaging and Chemical Analyses

The morphology, surface texture and chemical composition of the separated respirable samples were assessed by scanning electron microscope (SEM) imaging combined with energy dispersive X‐ray spectroscopy (EDS) analysis at LMV. High‐resolution images were collected on a Helios 5 field emission gun (FEG)‐SEM (*Thermo Fisher Scientific*) coupled with a focused ion beam (FIB; Xe plasma) in secondary electron (SE) and backscattered electron (BSE) modes (Eychenne et al., 2022). Images were collected at a minimum of 2.6 × 10^7^ pixels per image, and with pixel dimensions of about 15 nm. Elemental compositions were measured with a 60 mm^2^ annular *FLATQUAD* detector (*Bruker*) and an *XFlash* detector (*Bruker*). All data were collected on unpolished mounts of particles dispersed on polycarbonate membranes, stuck on carbon sticky tape and carbon coated. Beam conditions and working distances varied depending on the type of data collected and are reported in appropriate figures and captions.

One 100‐200 nm‐thick, electron transparent ion‐thinned section was extracted from one calcite particle in the GDD sample in order to characterize the coating found at the surface of the GDD particles. High magnification images were acquired in transmission mode at 30 kV, 0.1 nA and a 5 mm working distance in Bright Field (BF), Dark Field (DF) and High‐angle annular dark‐field (HAADF) imaging modes, using the Scanning Transmission Electron Microscopy detector (STEM) of the LMV Helios 5 electron microscope. Elemental compositions were measured by EDS in transmission mode using the *XFlash* detector at 30 kV, 1.6 nA and a 5 mm working distance.

#### 2.2.4 Sample Mineralogy

The mineralogy of the respirable ATD sample and GDD and SVA isolates was assessed by Raman spectroscopy at LMV on unpolished mounts of particles dispersed on carbon sticky tape, and on polished mounts of particles impregnated in low viscosity epoxy resin (*Logitech LTD*). The spectra were collected on individual particles using an InVia confocal Raman micro‐spectrometer (*Renishaw*) equipped with a 532 nm diode‐pulsed solid‐state laser (200 mW output power), a Peltier‐cooled CCD detector of 1,040 × 256 pixels, a motorized XYZ stage, and a Leica DM 2500 M optical microscope (Eychenne et al., 2022). The scattered light was collected using a back‐scattered geometry. The analytical settings used were a laser power at the grain surface between 0.1 and 1 mW, an acquisition time between 15 and 60 s, a grating of 2400 grooves mm^−1^, a 100X microscope objective and a 20 μm slit aperture (high confocality setting). Calibration of peak position prior to analysis was performed based on the 520.5 cm^−1^ peak of Si. Spectra were recorded in the wavenumber range 60–1,410 cm^−1^, which encompasses vibrational frequencies of mineral phases and aluminosilicate network domain of glasses, and occasionally in the 2,800–3,900 cm^−1^ range to check for H_2_O and OH molecules. Individual spectra were interpreted in terms of phase (mineral, glass or mixture of mineral(s) and glass) by fitting the main peaks or bands, using reference libraries (LMV internal database, RRUFF™ project and Thermo Fisher Grams Spectral ID^®^) and publications (Frezzotti et al., 2012; Schiavi et al., 2018). Between 50 and 100 spectra per sample were acquired and interpreted.

#### 2.2.5 Image Analysis

##### 2.2.5.1 Particle Morphology From SEM Images

SEM‐SE images were analyzed in ImageJ for morphometric analysis. Segmentation and measurement of particles was made in ImageJ via user‐led grayscale and size thresholding using the “Rock pie” set of macros (see Huebsch et al., 2023) available at https://github.com/hornbya/RockPie. Particles were measured using the *ShapeFilter* plugin (Wagner & Lipinski, 2013) to improve perimeter estimation compared to the native *Analyze Particles* function, thereby reducing pixelation artifacts on shape factors. Morphological parameters were quantified on particles with equivalent circular diameter >0.5 μm. We focused on two morphological parameters: (a) the aspect ratio, calculated as *D*_min_/*D*_max_, where *D*_min_ and *D*_max_ are the minimum and maximum Feret diameters of the particle, respectively, and (b) the solidity, calculated as *A*_*p*_/*A*_CH_, where *A*_*p*_ and *A*_CH_ are the areas of the particle and the convex hull, respectively. The aspect ratio quantifies the elongation of the particle, while the solidity quantifies the large scale (i.e., particle scale) irregularities of the particle contour (E. J. Liu et al., 2015). These two parameters have low sensitivities to particle resolution, and describe two features of particle morphologies, namely form and roughness.

##### 2.2.5.2 Phase Proportion Reconstruction

We developed a new image analysis macro to process sets of single‐element EDS maps and report proportions of phases (minerals and glass) defined by thresholding different combinations of element maps. The ImageJ macro “EDSphase mapper 2.1” (Hornby, 2023), described in the flow chart in Figure S1 in Supporting Information S1, is available on Github at https://github.com/hornbya/EDS_phase_mapper. The macro imports a full set of single‐element SEM‐EDS maps as a stack. For each phase, single or multiple grayscale element maps selected by the user are added to a substack and flattened using a median argument (i.e., a single image is created by using the median pixel value for all images in the substack). By applying a user‐determined binary threshold and denoising filters to the flattened image, a phase is defined and assigned a color. The element maps may be chosen freely, or guided by element intensities in a region of interest, or pre‐set for certain phases by selecting a named phase (e.g., “plagioclase feldspar”). The elements selected, defined phases, and RGB color choices are saved as tables. To avoid masking of smaller phases, the final image is reassembled by filling each phase in order of descending area before measuring the number of pixels for each color. Area proportions of phases are saved in a table, and a figure legend is generated matching phase colors, elements and area fractions.

For each sample, 3 to 4 EDS maps, each containing 20 to 60 individual grains, were analyzed to reach a total quantified particle area between 690 and 1,000 μm^2^. The identity of phases was independently constrained by considering the constituent element ratios (EDS spectra), the morphology and distribution in the particles, and by referring to phases identified a priori by Raman spectroscopy. In some instances, the method did not allow specific minerals and polymorphs to be defined; therefore, broad groups of phases are typically assigned (e.g., silica, pyroxene, iron oxides, etc.) and further definition of phases was obtained in conjunction with Raman spectroscopy data, where possible. User‐dependency of the results (phases identified and phase area proportions) was assessed by having the analyses performed independently by two users (A. Hornby and J. Eychenne) and quantifying the errors between the two users as difference in area proportion for each phase of each sample considered.

### 2.3 Oxidative Potential Measurement

To evaluate the OP of samples, we used two methods based on spectrophotometric measurements of depletion rate of target reagents oxidized by redox‐active/catalytic species present in PM, where one assay measures the consumption of dithiothreitol (DTT) and the other ascorbic acid (AA). The assays were performed at the University of Grenoble Alpes (Grenoble, France). Prior to OP measurements, particle samples (between 75 and 100 μg of bulk ATD and respirable isolates for GDD and SVA) were extracted in simulated lung fluid (SLF; Gamble's solution with dipalmitoylphosphatidylcholine as a lung surfactant) while vortexed at 37°C for 75 min in order to mimic lung bioaccessibility and closely simulate exposure conditions, following the methods of Calas et al. (2017).

The assays were performed twice (on two subsamples) for each sample. In both assays, the particle suspensions in SLF (the whole extract including soluble and insoluble constituents) were pipetted into wells of a 96‐well plate at concentrations of 2 μg of sample per well (corresponding to 6.25 μg/cm^2^) in duplicate (DTT) or triplicate (AA). The plate was kept at 37°C and auto‐shaken for 3 s before each measurement.

In the DTT assay, 12.5 nmol of DTT was used (50 μL of 0.25 mM DTT solution in phosphate buffer) to react with 205 μL of phosphate buffer and 80 μL of sample suspension in SLF. DTT depletion was determined by dosing the remaining amount of DTT with DTNB (dithionitrobenzoic acid) at different reaction times (0, 15 and 30 min), and absorbance was measured at 412 nm using a plate spectrophotometer (*Tecan*, M200 Infinite). The AA assay used is a simplified version of the synthetic respiratory tract lining fluid assay (Kelly & Mudway, 2003), where only AA is used. A reaction mix of 80 μL of sample suspension with 24 nmol of AA (100 μL of 0.24 mM AA solution in Milli‐Q water) was used, and AA depletion was read continuously for 30 min by absorbance at 265 nm (*Tecan*, M1000 Infinite). Both depletion rates of DTT and AA were determined by linear regression of the linear section data.

Three laboratory blanks were included in each experiment. The average values of these blanks were subtracted from the sample measurement of the plate. A limit of detection value was defined as three times the standard deviation of laboratory blanks measurements.

The OP_DTT_ and OP_AA_ results measured as oxidative activity of samples in nmol/min were normalized and expressed relative to sample mass (nmol/min/μg).

### 2.4 Microbiological Analysis

The presence of microbes in geogenic samples was assessed by performing microbial colony forming unit (CFU) counts in all samples, in two types of extracts at Centre Hospitalier Universitaire (CHU, Clermont‐Ferrand, France). Bulk samples were extracted with 10 mL of recovery solution (DNP (*Diluant Neutralisant Pharmacopée*) solution containing Polysorbate 80, lecithin, histidine, and thiosulfate; *BioMérieux*, France). Separately, we prepared extracts in cell culture medium by mixing 5 mg of bulk sample with 10 mL of complete cell medium (CCM) for alveolar or bronchial cultures used in the in vitro experiments (Section 2.5) followed by incubation at 37°C for 48 hr. Samples were then centrifuged for 5 min and supernatants recovered for microbiological analysis.

Each sample extract was streaked on R2A agar and Sabouraud agar, and one mL of extract was inoculated in two broths for the isolation of aerobic and anaerobic bacteria. The remaining extract for each sample (ca. 6.5 mL) was filtered (0.22 μm membrane) and deposited on blood agar. Each agar plate was incubated at 37°C for 48 hr. Results were expressed as a number of microorganisms (as CFUs) per filtered volume of particle extract. The maximum quantifiable CFU was set at 150 according to bacteriological standards.

The species/genera identification was done macroscopically and microscopically, and with conventional biochemical methods. Isolates were tested for Gram reaction, catalase production, oxidase production, and using the analytical profile index (API) biochemical tests (*BioMérieux*, France).

### 2.5 In Vitro Bioreactivity Assessment

#### 2.5.1 Lung Cell Cultures

Cell culturing and experiments were done at the Institute of Genetics, Reproduction and Development (iGReD, Clermont‐Ferrand, France). Cell culture consumables including cell culture media, fetal bovine serum (FBS), antibiotics (streptomycin, penicillin, amphotericin B), and phosphate buffered saline (PBS) were obtained from *Fisher Scientific* (Illkirch, France).

The alveolar type II epithelial cell line A549 was cultured in Roswell Park Memorial Institute medium (RPMI 1640) supplemented with 10% heat‐inactivated FBS, 1% antibiotics and 1% (2 mM) L‐glutamine at 37°C in a humidified incubator in a 5% CO_2_ atmosphere. The supplemented RPMI 1640 is further referred to as the CCM for alveolar cultures (aCCM). Bronchial epithelial cell line BEAS‐2B was grown in Dulbecco's Modified Eagle Medium/Nutrient Mixture F‐12 Ham (DMEM/F‐12) with GlutaMAX™ supplemented with 10% FBS and 1% antibiotics on collagen‐coated tissue flasks. The supplemented DMEM/F‐12 is further referred to as the CCM for bronchial cultures (bCCM). Both cell lines were subcultured when ∼80% confluent and used in experiments at passages between 9 and 20.

For the exposure experiments, A549 cells were harvested and seeded onto cell culture inserts (*Transwell^®^‐Clear 3450*, 24 mm diameter inserts, polyester membrane, 0.4 μm pore size, 4.67 cm^2^ growth area; *Corning Incorporated,* USA) at a density of 0.5 × 10^6^ cells in 2 mL aCCM per insert, corresponding to 1.07 × 10^5^ cells/cm^2^ in a 6‐well plate setting. Inserts were placed in culture plates and cells were grown under submerged conditions for 2 days (2 mL of aCCM in the apical and 3 mL in the basal compartment) before they were transferred to ALI conditions by removing the medium from the apical compartment and replacing the medium in the lower compartment with 1.2 mL fresh aCCM.

BEAS‐2B cells were harvested and seeded onto cell culture inserts pre‐coated with collagen (0.6 mL per insert for 1 hr in the incubator) at a density of 1 × 10^6^ cells in 2 mL bCCM per insert, corresponding to 2.14 × 10^5^ cells/cm^2^ in a 6‐well plate setting. Inserts were placed in culture plates and cells were grown under submerged conditions for 5 days (2 mL of bCCM in the apical and 3 mL in the basal compartment). The medium was changed and replaced with fresh bCCM 2 days after the cell seeding. On day 5, the cells were transferred to ALI conditions by removing the medium from the apical compartment and replacing the medium in the lower compartment with 1.2 mL fresh bCCM. Both cell lines were kept under ALI conditions for 24 hr before the particle exposures (Barosova et al., 2021).

#### 2.5.2 Lung Cell Exposures

Prior to the exposures, the particles were suspended in 1 mL of respective serum‐free CCM (aCCM and bCCM without FBS) and vigorously vortexed, before being diluted to working concentrations of 4.67 and 467 μg/mL. The cells at the ALI were exposed to 100 μL of particle suspension which is a method referred to as pseudo‐ALI or quasi‐ALI (Endes et al., 2014; Tomašek et al., 2016), allowing an accessible ALI exposure in the absence of an aerosol exposure system (Meldrum et al., 2022). The used doses correspond to particle depositions of 0.1 and 10 μg/cm^2^, respectively, considering that a majority of particles deposit on the cells and taking into account the growth area of the insert.

The cell‐delivered particle doses used in this study were based on previous studies investigating volcanic ash effects to lung cells cultured at the ALI (Tomašek et al., 2016, 2018). In those studies, the ash doses range between 0.2 and 1 μg/cm^2^ and were determined to represent a particle over‐load scenario relative to a real‐life exposure following a volcanic eruption (Tomašek et al., 2016). As with eruptions, ambient concentrations of desert dusts can be highly variable and are poorly constrained, but the doses used in this study also likely deviate from realistic inhalation exposure. However, the assessment of cellular responses herein is intended as a direct comparison of differences in bioreactivity for the diverse geogenic particle types. The limited amounts of sample available for the study, particularly GDD, only allowed for a four‐point dose‐dependent analysis of cytotoxicity and pro‐inflammatory response for ATD and SVA samples in one cell line (A549). These data, which include cellular response to 50 and 100 μg/cm^2^ particle treatments for the two samples, are reported in Figure S2 in Supporting Information S1.

The experiments with A549 cells were performed in triplicate or duplicate and repeated three times. The experiments with BEAS‐2B cells were performed in duplicate and repeated four times. In all experiments, the negative control cells (untreated cells) were treated with 100 μL of appropriate serum‐free CCM on the apical side of the culture.

#### 2.5.3 Cellular Assays and Analysis

##### 2.5.3.1 Cytotoxicity

Cell death was assessed by measuring the release of the intracellular enzyme lactate dehydrogenase (LDH) into the culture medium, which indicates the loss of cell membrane integrity. The level of LDH activity in the culture medium was measured using an automated enzymatic assay (Vista, *Siemens Health Diagnosis*, France) according to the manufacturer's guidelines at CHU Clermont‐Ferrand (France). To lyse the cells and release the maximum LDH, positive control cells were treated with 100 μL of 0.2% Triton X‐100 in PBS on the apical side and incubated for 24 hr at 37°C and 5% CO_2_.

##### 2.5.3.2 Quantification of Pro‐Inflammatory Response

The pro‐inflammatory response was investigated for both cell lines by quantifying interleukin‐6 (IL‐6), interleukin‐8 (IL‐8), interleukin‐1 beta (IL‐1β) and tumor necrosis factor alpha (TNF‐α) release into the culture medium using an automated multiplex immunoassay on Ella™ (*ProteinSimple*, USA) at CHU Clermont‐Ferrand (France). Lipopolysaccharide (LPS, from *E. coli* at 1 μg/mL for BEAS‐2B experiments and 10 μg/mL for A549 experiments) was applied in 100 μL of serum‐free CCM on the apical side of the culture for 24 hr and served as the positive control for cytokine induction. Analyses were conducted in triplicate for each replicate.

Cytokine concentrations were normalized to total protein concentration of each corresponding sample, which was determined with the Pierce BCA Protein Assay kit (*Pierce Protein Research Products*, USA) according to the manufacturer's instructions. The results are reported as fold change of treated/untreated.

##### 2.5.3.3 Data Processing and Statistical Analysis

Data visualization and statistical analyses were performed using the software Prism (version 10.2.3, *GraphPad Software*, USA). All data are presented as the median with range. Statistical significance was deduced through the use of nonparametric Kruskall‐Wallis test, followed by Dunn's post‐hoc test to determine significance between different exposures and the untreated cells. Differences were considered significant at *p* ≤ 0.05.

## 3 Results

### 3.1 Particle Size Distribution and Morphology

**Figure 1:**
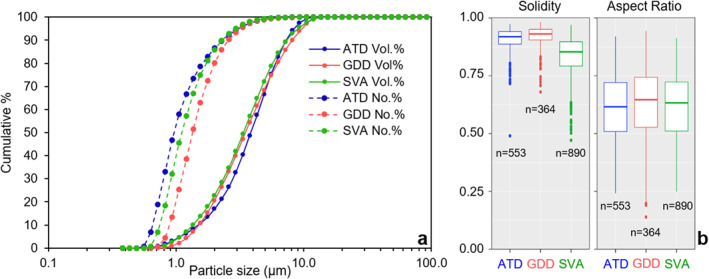
Particle size and morphology distributions of the Arizona test dust (ATD), Gobi Desert dust (GDD) and St. Vincent volcanic ash (SVA) respirable samples. (a) Cumulative particle size distributions in volume percent (Vol.%) and number percent (No.%) of the three samples acquired by laser diffraction. (b) Particle solidity and aspect ratio distributions in the three samples obtained by image analyses of particles with equivalent circular diameters >0.5 μm in scanning electron microscope images (*n* is the number of particles measured). Distributions are presented as Tukey box and whisker plots, where the thick middle line is the median, the lower and upper hinges mark the 25th and 75th percentiles, the lower and upper whiskers extend to 1.5 × the interquartile range (i.e., distance between the first and third quartiles), and outliers are plotted individually.

The volume‐based particle size distributions of the three respirable geogenic samples measured by laser diffraction are very similar (Figure 1a), with median diameters of 3.9, 3.5, and 3.4 μm for the ATD, GDD and SVA samples, respectively (Table 1). The respective ranges of sub‐4, sub‐2.5 and sub‐1 μm particle contents in the three samples are 52–60, 25–33 and 2–4 Vol.% (Table 1).

**Table 1:** Median Diameter and Proportions of Particles Finer Than 4, 2.5 and 1 μm of the Particle Size Distributions Acquired by Laser Diffraction, Expressed as Volume % and Number %

	Volume‐based distribution (%)			Number‐based distribution (%)		
	ATD	GDD	SVA	ATD	GDD	SVA
Median diameter (μm)	3.9	3.5	3.4	1.0	1.3	1.1
Particles <10 µm	99	97	96	100	100	100
Particles <4 µm	52	57	60	98	98	98
Particles <2.5 µm	25	31	33	92	89	92
Particles <1 µm	4	2	4	53	20	40

The number‐based particle size distributions show a shift in the fineness ordering among the three respirable samples, with ATD displaying the finest and GDD the coarsest distributions (Figure 1a), mostly due to their contrasting number‐based content in sub‐1 μm fraction (53, 40, and 20 no.% for ATD, SVA, and GDD, respectively; Table 1). The sample median diameters are 1.0, 1.1, and 1.3 μm for ATD, SVA and GDD, respectively (Table 1).

The aspect ratio distributions of the three respirable samples are approximately equivalent, with similar medians (0.62–0.65) and interquartile ranges (Figure 1b). The two desert dust samples, ATD and GDD, have similar solidity distributions, with medians of 0.92 and 0.93, respectively (Figure 1b). The SVA sample shows lower solidity values, with a median of 0.85 (Figure 1b), which indicates that, at the particle scale, SVA particles have more irregular contours than the dust samples.

### 3.2 Bulk Sample Chemical Composition

Bulk oxide elemental data for samples are listed in Table 2 and indicate the differences in composition of the geogenic PM samples. The silicon dioxide (SiO_2_) content varies among the samples, with the lowest content in SVA (54 wt.%), in good agreement with a magmatic rock of basaltic‐andesite composition, highest in ATD (74 wt.%), and intermediate content measured in GDD (63 wt.%). The SVA sample is comparatively richer in Al_2_O_3_, Fe_2_O_3_, MgO and Na_2_O than desert dust samples. In turn, desert dust samples contain higher proportion of K_2_O than the investigated volcanic ash. Contrasting values of loss on ignition at 1000°C are found among the samples, with values of 8.9 and 3.8 wt.% for GDD and ATD respectively, and 0.2 wt.% for SVA, corresponding to the total carbon content of the samples (including all carbonates) and possibly sample hydration.

**Table 2:** Bulk Chemical Compositions of the Geogenic Particle Samples Used in the Study

	SiO_2_	Al_2_O_3_	Fe_2_O_3_	MgO	CaO	Na_2_O	K_2_O	TiO_2_	MnO	P_2_O_5_	Other	LOI_110_	LOI_1000_	Total
ATD	74.3	8.9	2.6	1.2	2.9	1.6	2.3	0.4	0.1	0.1	0.08	0.6	3.8	98.9
GDD	63.4	9.0	3.5	1.6	9.1	1.8	2.0	0.6	0.1	0.1	0.07	0.3	8.9	100.3
SVA	53.9	19.1	8.0	3.6	8.7	3.5	0.6	0.9	0.2	0.1	0.01	0.4	0.2	99.2

*Note.* Results are presented as component weight percent (wt.%). LOI_110_ and LOI_1000_ correspond to the loss on ignition at 110 and 1000°C, respectively.

### 3.3 Sample Mineralogy

The main constituent mineral phases identified by Raman spectroscopy in the three respirable samples (Supplementary file 1 in Supporting Information S1) are as follows: ATD: quartz (crystalline silica), plagioclase (calcium‐sodium feldspar), potassium feldspar, magnetite (iron oxide), hematite (iron oxide), goethite (iron oxide), mica, calcite, and occasional olivine, pyroxene, sodalite, halite; GDD: calcite and other carbonates, hematite, magnetite, mica, quartz, plagioclase, potassium feldspar, clay (vermiculite‐type), and occasional anatase (titanium oxide), silicate glass, epidote; SVA: dacitic and rhyolitic glass, plagioclase, pyroxene, quartz, cristobalite (crystalline silica), hematite, magnetite, ilmenite (iron‐titanium oxide), pyrite (iron sulphide), anhydrite (calcium sulfate), alunite and natroalunite (aluminum potassium sulfate), sylvite (potassium chloride).

**Figure 2:**
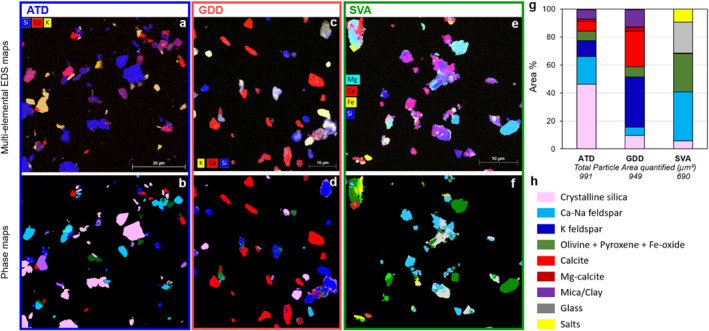
Phase quantification in the three respirable samples Arizona test dust (ATD), Gobi Desert dust (GDD) and St. Vincent volcanic ash (SVA), performed by image analyses of the EDS maps acquired with the FlatQuad detector using the ImageJ macro “EDS‐pie.” Example multi‐elemental EDS maps are shown in (a), (c) and (e). Color keys are displayed on the maps. The corresponding phase map constructions are shown in (b), (d) and (f). Color key is in (h). The phase salts is inclusive of all identified sulfates/sulphides and chlorides. Phase proportions (in area %) for the three samples (calculated from 3 to 4 maps for each, all provided in Supplementary file 2 in Supporting Information S1) are shown in (g) with the corresponding color key in (h). The values for the “total particle area quantified” correspond to the mean of the two users (due to differences in image cropping). In (f), the brown phase corresponds to Fe‐Ti oxides, that are counted together with olivine and pyroxene in (g).

Image analyses of the EDS maps (Figure 2, Table S1 and Supplementary file 2 in Supporting Information S1) demonstrate that: (a) ATD is dominated by crystalline silica (quartz according to Raman data; 46 area %) and feldspar (potassium feldspars and calcium‐sodium feldspars; 31 area %), followed by calcite (including magnesium calcite, 8 area %), mica/clay (7 area %), magnesium and iron rich minerals (pyroxene, iron oxides and olivine according to Raman data; 7 area %); (b) GDD is dominated by feldspar (potassium feldspars and calcium‐sodium feldspars; 42 area %) and calcite (calcite and magnesium calcite; 28.5 area %), followed by mica/clay (12 area %), crystalline silica (quartz according to Raman data; 10 area %), magnesium and iron rich minerals (pyroxene and iron oxides according to Raman data; 7 area %); (c) SVA is dominated by calcium‐sodium feldspar (35 area %; no potassium feldspar was observed in the volcanic ash) and glass (22 area %), followed by pyroxene (clinopyroxene and orthopyroxene according to Raman data; 22 area %), surface salts (anhydrite, alunite, natroalunite, sylvite; 9 area %), crystalline silica (quartz and cristobalite according to Raman data; 6 area %) and iron‐titanium oxides (5 area %). The errors between the two users vary between 0.1 and 5.3 area %, depending on the sample and phase considered (Table S1 in Supporting Information S1). These errors can be considered low based on the variability among the three samples which is higher than the errors quantified (Figure 2g). Note that despite pyroxene, iron oxides and olivine being grouped in Figure 2g, no olivine was observed by Raman in our GDD and SVA samples.

### 3.4 Particle Surface Texture and Chemistry

**Figure 3:**
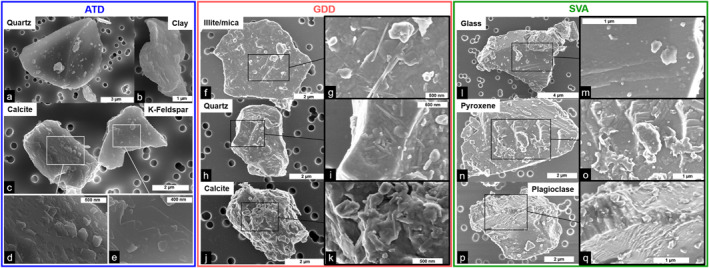
Surface texture of respirable particles from the Arizona test dust (ATD), Gobi Desert dust (GDD) and St. Vincent volcanic ash (SVA) samples imaged by scanning electron microscopy in secondary electron mode. (a–e) ATD particles imaged at 10 kV, 0.1 nA and a working distance of 5 mm. (f–k) GDD particles imaged at 2 kV, 50 pA and a working distance of 4 mm. (l–q) SVA particles imaged at 2 kV, 50 pA and a working distance of 4 mm.

**Figure 4:**
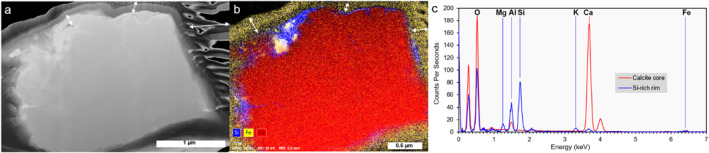
Chemical composition of adhering grains present at the surface of Gobi Desert dust (GDD) particles. (a) Scanning Transmission Electron Microscopy (STEM) image in Dark Field (DF) mode of the electron transparent thin section extracted from a calcite particle. (b) EDS map of the thin section in (a) showing the distribution of silicon (Si; blue), iron (Fe; yellow) and calcium (Ca; red). The yellowish outer rim, as indicated by the white arrows in (a) and (b), represents the platinum‐based protective layers deposited on the calcite grain before extraction of the thin section. (c) EDS spectra of the core of the calcite particle (red spectrum) and the silica‐rich rim (blue spectrum) shown in blue in (b). The unlabeled peaks at 0.9 and 2.0 keV in (c) are the L band of copper (material constituting the thin section stand) and the M band of platinum, respectively.

SEM images confirm that the three respirable samples mainly contain particles 1–10 μm in size with heterogeneous surface textures (Figure 3). Irrespective of their mineralogy, particles from all the samples show surfaces characterized by the presence of adhering grains. Within the ATD sample, these surface grains are generally finer than 500 nm with equant shapes (Figures 3a–3e). They are Fe‐oxides (often <100 nm in size), calcite, quartz and various aluminosilicates (Supplementary file 3 in Supporting Information S1). Within the GDD sample, the surface grains are densely distributed (Figures 3f–3k). They are predominantly elongated, up to 1 μm in length and a few tens of nm in width, or are shaped as flakes, less than 500 nm in size. The electron transparent thin section of a calcite particle (Figure 4) shows that these surface grains create a “crust” of silicates (Figures 4b and 4c) whose dominant compositions matches with a clay mineral (Figure 4c, Table S2 and Supplementary file 3 in Supporting Information S1), possibly illite (mineral containing silicon, aluminum, magnesium, iron and potassium) and/or vermiculite (observed by Raman). Within the SVA sample, surface grains are mostly equant and less than 500 nm in size (Figures 3l–3q). They are Fe‐Ti‐oxides, pyroxene, plagioclase and glass fragments, as well as sulfur and phosphorus bearing phases (Supplementary file 3 in Supporting Information S1). Regularly spaced, sub‐rounded nodules smaller than 10 nm in size can also be observed preferentially on the surface of glassy particles (Figure 3m). Surfaces of glassy particles are enriched in chlorine compared to other grains (Supplementary file 3 in Supporting Information S1), but specific Cl‐bearing surface‐adhering phases cannot be identified at the resolution of our study.

The intrinsic surfaces of the particles (i.e., below the adhering surface grains) are also variable between the three samples. Within the ATD sample, quartz and feldspar particles have generally smooth surfaces, except for stepped and conchoidal fractures and cleavage plans (Figure 3e). Calcite crystals show rough surfaces suggesting dissolution (Figures 3c and 3d). Clay is characterized by sheet‐like surfaces (Figure 3b). Within the GDD sample, the surface grains form such a dense network that the intrinsic particles' surfaces cannot be observed (Figures 3f–3k). The nano‐scale surface roughness created by the network of surface grains suggests that the specific surface area of GDD particles will be higher than those of ATD and SVA particles. Within the SVA sample, particle surfaces are generally smoother than in the ATD and GDD samples, with fractures and cleavage plans on mineral phases and glass (Figures 3m, 3o, and 3q).

### 3.5 Presence of Microorganisms

Geogenic particle samples were cultured under several culture conditions (i.e., different extraction and growth media, with or without antibiotics) to allow for better isolation and identification of microorganisms present. We detected the presence of bacteria in extracts of both desert dust samples but not in volcanic ash (SVA), which was found to be sterile (i.e., with no detectable viable bacteria, CFU <1; Table 3). While ATD and GDD samples overall had similar bacteria levels (CFU >150 in recovery solution), culturable bacterial concentrations were the highest in the GDD sample under lung cell culture conditions (aCCM and bCCM; Table 3). The results indicate that these samples are polymicrobial, considering the differences in detected colonies and identified microorganism types across the different extraction media.

**Table 3:** Bacterial Colony Forming Units (CFU) per Volume of Analyzed Extract for Arizona Test Dust (ATD), Gobi Desert Dust (GDD) and St. Vincent Volcanic Ash (SVA) Samples

Dust sample extracts	Recovery solution	aCCM	bCCM
ATD	>150	16	<1
*Microorganism*:	*Bacillus sp.*	*Bacillus sp.*	
GDD	>150	31	80
	*Bacillus sp.*	*Actinomyces sp.*	*Actinomyces sp.*
*Microorganism*:	*Corynebacterium sp*.		
SVA	<1	<1	<1

*Note.* Cell culture medium (aCCM and bCCM; Section 2.5.1) contains antibiotics (1%). A value of <1 equates to no detectable viable bacteria.

The bacterial isolates identified in ATD were from *Bacillus* sp., while the GDD sample also contained *Corynebacterium* sp. and *Actinomyces* sp. (Table 3). The *Bacillus* sp. present in ATD are likely from multiple strains, as indicated by the lower CFU in aCCM and the fact that no colonies were viable in bCCM. Similarly, it can be postulated that GDD contains different subtypes of *Bacillus* sp. than ATD, as no colonies of *Bacillus* sp. were identified in the complete cell culture media extracts (containing antibiotics). It has to be noted that the identified bacteria are only a subset of total viable organisms (CFUs) detected.

### 3.6 Oxidative Potential

**Figure 5:**
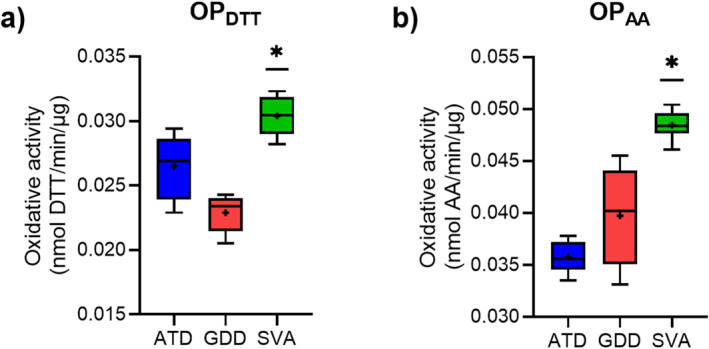
Oxidative potential (OP) of the three respirable geogenic samples: Arizona test dust (ATD), Gobi Desert dust (GDD) and St. Vincent volcanic ash (SVA). Data are expressed as oxidative activity (rate of reagent consumption) normalized to sample mass in nmol/min/μg, as determined by measurement of depletion of (a) dithiothreitol (OP_DTT_) and (b) ascorbate (OP_AA_). Data are presented as Tukey boxplots with the mean represented as a + and the median as a horizontal line. Data are from measurement replicates *n* = 16 for OP_DTT_ and *n* = 12 for OP_AA_. Statistical significance between OP of different samples is indicated as * (*p* ≤ 0.05).

Results of the OP measurements for the respirable samples are shown in Figure 5. Among the samples, SVA shows the highest reactivity in both OP assays (Figure 5), which is significantly different (*p* ≤ 0.05) from the reactivity of both desert dust samples. Desert dust samples exhibit statistically comparable activities, however, with higher mean oxidative activity of ATD in the DTT assay (Figure 5a) and higher mean AA activity for the GDD sample (Figure 5b). Overall, all samples exhibit higher potency to deplete AA in comparison to DTT, as can be seen by the higher maximum activity values measured in this assay (Figure 5b). Mean OP values normalized to mass for ATD, GDD and SVA samples, respectively, are 0.027, 0.023 and 0.030 nmol/min/μg for OP_DTT_ and 0.036, 0.040, and 0.049 nmol/min/μg for OP_AA_.

### 3.7 Lung Cell Response

**Figure 6:**
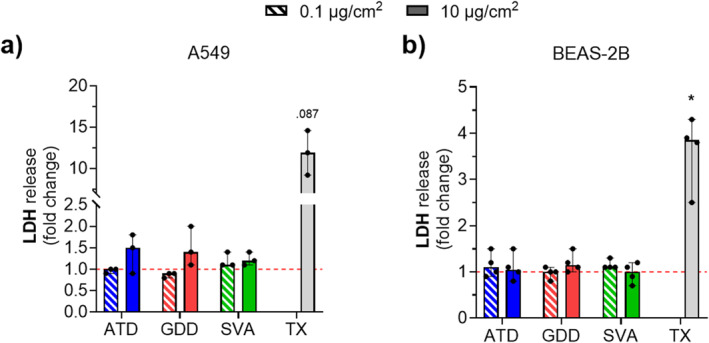
Cytotoxicity toward (a) A549 and (b) BEAS‐2B cells of the respirable Arizona test dust (ATD), Gobi Desert dust (GDD) and St. Vincent volcanic ash (SVA) at two doses (0.1 and 10 μg/cm^2^) after 24 hr exposure at an ALI, measured by lactate dehydrogenase (LDH) activity. LDH release is expressed as a fold change relative to untreated cells (serum‐free cell culture medium only) measured from three (A549 *N* = 3) or four (BEAS‐2B *N* = 4) independent experiments. Triton X‐100 (TX) at 0.2% in phosphate buffered saline acted as the positive assay control. The data are presented as the median with range. The points are the average value of individual measurements of the replicates within an experiment. Statistical significance between treated and untreated cells is indicated as * (*p* < 0.05).

The particle‐induced cytotoxicity after 24 hr was low for both doses of all samples in both cell models, as seen by the limited increase in LDH release compared to the untreated cells (Figure 6), indicating little acute effect of the particle treatments on cell viability. Moreover, there was no statistical difference in LDH release among different particle types.

**Figure 7:**
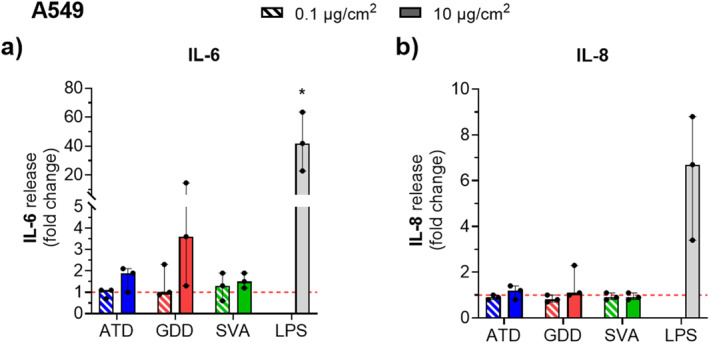
Pro‐inflammatory response in alveolar epithelial cells (A549) assessed by cytokine production following acute exposure to the respirable Arizona test dust (ATD), Gobi Desert dust (GDD) and St. Vincent volcanic ash (SVA) at two doses (0.1 and 10 μg/cm^2^) measured after 24 hr exposure at an ALI in culture supernatants on duplicates or triplicates of three independent experiments (*N* = 3). Protein production is presented as the cytokine concentration normalized to the total protein concentration and expressed as a fold change relative to untreated cells for (a) IL‐6 and (b) IL‐8. Lipopolysaccharide (LPS, from *E. coli*, at 10 μg/mL) was used as a pro‐inflammatory stimulant. Data are presented as the median with range. The points are the average value of individual measurements of the replicates within an experiment. Statistical significance between treated and untreated cells is indicated as * (*p* < 0.05).

No significant (*p* > 0.05) pro‐inflammatory response was found for any particle, as measured for chosen markers in the A549 alveolar epithelium cell model (Figure 7), although we observed increased production of IL‐6 compared to the untreated cells (i.e., exposed to aCCM, alone) for ATD and GDD samples at the high dose (10 μg/cm^2^). Similar, slightly elevated release was observed for IL‐8 after exposure to the high dose of desert dusts. No production of TNF‐α or IL‐1β was recorded after 24 hr particle exposures (data not shown). Overall, the secretion of cytokines in A549 appears to be stimulated more by desert dust particles than volcanic ash, with the most prominent response seen upon exposure to the GDD sample which, on average, induced the highest levels of detected cytokines (without reaching statistical significance).

**Figure 8:**
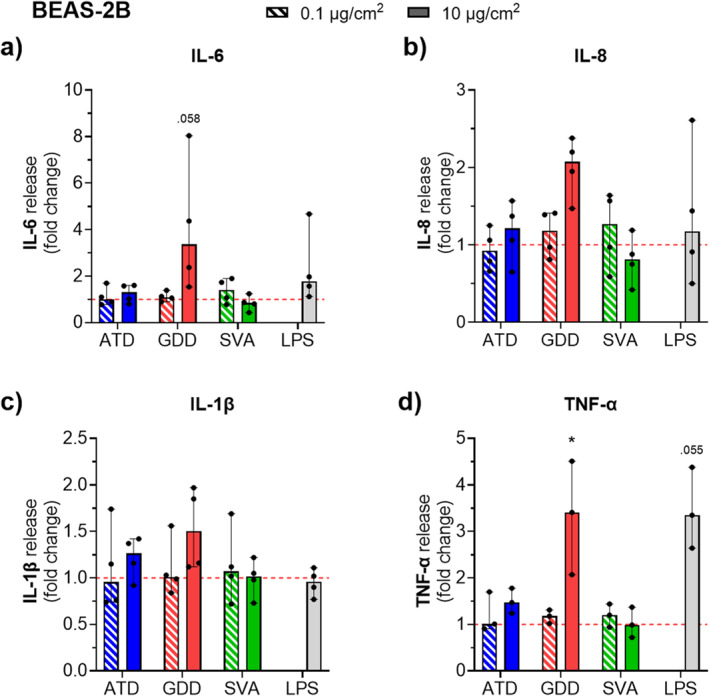
Pro‐inflammatory response in bronchial epithelial cells (BEAS‐2B) assessed by cytokine production following acute exposure to the respirable Arizona test dust (ATD), Gobi Desert dust (GDD) and St. Vincent volcanic ash (SVA) at two doses (0.1 and 10 μg/cm^2^) measured after 24 hr exposure in culture supernatants on duplicates of four independent experiments (*N* = 4). Protein production is presented as the cytokine concentration normalized to the total protein concentration and expressed as a fold change relative to untreated cells for (a) IL‐6, (b) IL‐8, (c) IL‐1β, and (d) TNF‐α. The background level of TNF‐α (i.e., in untreated cells) was unquantifiable in one experiment, and therefore, a fold change could not be calculated for this experiment and the data are presented for *N* = 3. Lipopolysaccharide (LPS, from *E. coli*, at 1 μg/mL) was used as a pro‐inflammatory stimulant. Data are presented as the median with range. The points are the average value of individual measurements of the replicates within an experiment. Statistical significance between treated and untreated cells is indicated as * (*p* < 0.05).

A similar trend in responses was observed in the BEAS‐2B bronchial epithelial cell model when it comes to the tendency for increased production of all investigated cytokines upon particle exposures compared to their background levels (Figure 8). A dose‐dependent response of this model can be seen for desert dust samples (ATD and GDD) but not for volcanic ash. Moreover, in BEAS‐2B cells, GDD induced the highest levels of all cytokines compared to other samples, specifically for the high dose (10 μg/cm^2^), which induced a significant (*p* < 0.05) release of TNF‐α (Figure 8d).

## 4 Discussion

Given our aim to explore differences in physicochemical properties and bioreactivity of various geogenic particles, we selected a suite of mineral dust samples with contrasting charateristics and origins, in order to represent different geogenic particle types that are present in the Earth system. The SVA sample was chosen because it is representative of andesitic explosive eruptions of mid‐intensity, which are the most frequent explosive eruptions on Earth and produce the majority of volcanic ash by mass (Galetto et al., 2023; Siebert et al., 2015). It is a fresh sample that was collected soon after it formed by magmatic fragmentation, and therefore is different from the desert dust samples, which are weathered materials that have been exposed to environmental conditions for an extended period of (geologic) time (Langmann, 2013). We also chose to include one standardized desert dust sample representative of particles collected from the source (ATD), which was industrially reprocessed and would have comparatively fresh surfaces, and one desert dust sample representative of dust storm particles (GDD), which are “wind‐blown” or “ambient” dusts sampled at a location distal from the source.

While these samples allow us to explore some key differences between various mineral dusts (e.g., fresh vs. altered material, transported vs. in situ particles), it is important to acknowledge that the desert dust samples we studied are not representative of dusts from other desert regions, nor that the volcanic ash sample chosen is representative of all volcanic ash types. Desert dust properties vary significantly based on the environmental, geological, and meteorological conditions of the source areas. As a result, desert dusts from different sources contain varying amounts of typical minerals (e.g., quartz, clay, carbonates, etc.), as demonstrated here by the different mineralogical content of ATD and GDD (Figure 2). Additionally, during dust transport, processes such as atmospheric aging and interactions with atmospheric pollutants can further alter particle characteristics. Hence, even when originating from the same source, dusts may carry different amounts of adsorbed pollutants (e.g., metals, sulfates, nitrates, organic compounds) and biological materials (Israelevich et al., 2003; Moreno et al., 2006). Similarly, there can be substantial differences in physicochemical properties between volcanic ash from different volcanoes and different phases of an eruption (Damby et al., 2017). Ash composition is largely dictated by magma composition (e.g., basaltic, andesitic, rhyolitic), but it can also be altered through physicochemical interactions within volcanic and urban atmospheres (Stewart et al., 2020; Tomašek, Damby, Andronico, et al., 2021). Therefore, while the outcomes of this study will allow interpretation of key differences in bioreactivity as a function of the physiochemical properties of different geogenic particle types, they cannot be universally transferred to any other desert dust or volcanic ash.

### 4.1 Physicochemical Properties of Geogenic Particles

The particle size distributions of the respirable samples were comparable (Figure 1a), allowing us to appropriately assess the differences in other sample properties (mineralogical composition, morphology and surface properties) in the same size fraction (PM_10_).

Bulk chemical composition of volcanic ash is mainly determined by the magma from which it is generated. The studied ash (SVA) is classified as basaltic andesite (54 wt.% SiO_2_ and 4.1 wt.% Na_2_O + K_2_O; Table 2), which is consistent with published petrological data acquired for the explosive deposits of the 2021 La Soufrière volcano, St. Vincent eruption (Frey et al., 2024; Horwell et al., 2024). Major element analysis showed that the bulk compositions of ATD and GDD are dominated by SiO_2_, suggesting that they contain silicate mineral phases (Table 2). These two samples also contain a significant proportion of carbonates (e.g., limestone fragments) and potentially hydrated minerals (such as clay), as indicated by the values of loss on ignition at 1000°C (Table 2). These results are in agreement with what is known about desert dust composition (Adebiyi et al., 2023; Querol et al., 2019) and earlier analyses on these ATD and GDD samples (Joshi et al., 2017; Urupina et al., 2019).

Despite some similarities in the main constituent phases identified in the volcanic ash and desert dust samples (e.g., crystalline silica, calcium‐sodium feldspar, pyroxene, iron‐titanium oxides), some important differences can be noted, such as the presence of calcite, clay and potassium feldspar in the desert dusts that are entirely absent in the volcanic ash sample, and the presence of sulfate salts, as well as abundance of glass and chloride salts in the volcanic ash (Figure 2). In terms of proportions, the volcanic ash sample is dominated by calcium‐sodium feldspar (plagioclase) and silicate glass, while ATD is dominated by quartz and GDD by potassium feldspar and calcite (Figure 2). This is consistent with the bulk chemical composition of the samples (Table 2), and with the generally reported mineral load in dust storm events which is mainly made up of quartz, a variety of clays, feldspars, and variable contents of carbonate minerals (mostly calcite and dolomite) and iron oxides (Adebiyi et al., 2023; Querol et al., 2019). The differences in phases and their proportions between the two desert dust samples are most likely associated with varying mineralogy of the dust source regions and the different degrees of weathering of geological terrains, which generally leads to the depletion of primary minerals and formation of secondary minerals such as clays (Formenti et al., 2011; Journet et al., 2014; Langmann, 2013; Querol et al., 2019). The presence of pyroxenes and abundant quartz, as well as feldspars and mica, in ATD and GDD (Figure 2) suggest the influence of igneous rocks and sediments on the desert basins, while carbonates, which are absent in volcanic ash, suggest a strong sedimentary influence. Whereas ATD is reprocessed and sourced from a well‐defined area (Salt River Valley, Arizona, USA), it cannot be ruled out that some material not associated with the dust source was integrated during transport of the GDD sample. The componentry of the volcanic ash sample is consistent with the magma composition that fed the 2021 La Soufrière volcano, St. Vincent eruption and the eruptive dynamics of the early explosive phase (Frey et al., 2024; Robertson et al., 2024; G. Weber et al., 2024), which incorporated some dome rock fragments (explaining the presence of crystalline silica, the rhyolitic glass, and the rare mica) as well as fresh volcanic particles (accounting for the other phases identified) that interacted with volcanic gases (leading to the formation of the salts). Note that the differences in phase’ proportions between the three samples discussed above are well above the error associated with the phase quantification method and the variability between users (Figure 2 and Table S1 in Supporting Information S1), and can thus be reliably interpreted in terms of variability between samples.

The particle morphology data indicate that all the samples contain particles with similar aspect ratios, while the volcanic ash clearly shows more irregular contours than the desert dusts (Figure 1b). This is also what the SEM observations qualitatively show (Figure 3) and can be explained by the remnant vesicle (bubble wall) shapes on the ash particles created by the fragmentation of the bubbly magma. Despite such differences in the particles' contour irregularities, particles from all samples are generally angular with varying amounts of sub‐micron particles adhering to the surfaces of larger particles (Figure 3). Conversely, we found that the overall surface texture of particles, including the nature of adhering grains, varies among the samples. These differences are most probably associated with the different origins and history of the samples, including the atmospheric (while suspended) and/or environmental (while deposited) processing of particles at various stages of their “life cycle.” The smoothest surfaces are found on glassy volcanic ash particles that are pre‐fragmentation features (walls of liquid between the gas bubbles in the rising magma). In our ash sample, particle surfaces are relatively clean because the particle spent minimal time in the environment before sampling (i.e., no weathering), contrary to the desert dust samples which show more complex surface textures and features (Figure 3). Volcanic ash did undergo processing during transport in the volcanic plume where it interacted with volcanic gases, where the sulfur and chlorine salts formed (Supplementary files 1 and 3 in Supporting Information S1). Particles within the ATD sample are either smooth (quartz) or rough (calcite, clay, mica), depending on the componentry (Figures 3d and 3e), which could be associated with sample processing (jet milling) and dissolution/leaching (while in the environment), respectively. The particle surfaces of the GDD sample stand out among the samples as they are completely covered by nano‐scale, elongated or flake shaped, clay minerals, possibly illite or vermiculite (Figure 4 and Table S2 in Supporting Information S1), a chemical weathering product that occurs in almost all soils across the globe (Journet et al., 2014). These differences in particle surfaces have implications for potential reactivity of the samples, as their texture, chemistry and componentry dictate the nature, distribution and abundance of reactive surface sites and, in this way, how the particles interact at various interfaces (e.g., with atmospheric gases or in contact with biological fluids or tissues) (Maters et al., 2016; Zeineddine et al., 2023).

### 4.2 Particle‐Associated Microorganisms

Our microbiological analyses revealed the presence of viable bacteria in desert dust samples, while we did not find any detectable bacteria in volcanic ash (Table 3). It is well established that desert dust can contain a wide variety of microorganisms, including fungi, bacteria and viruses, some of which are capable of causing disease in a range of organisms, including plants, animals and people (Gonzalez‐Martin et al., 2014; Kellogg & Griffin, 2006). In our investigation, we found *Bacillus* sp. in both ATD and GDD samples (Table 3), which is a spore‐forming genus of bacteria that can survive long‐range transport and is commonly recovered in dust‐storm microbiology studies (Griffin, 2007). It is also the species that were identified as dominant during Asian dust events for which the Gobi Desert is the major source (Hagiwara et al., 2021; Yoo et al., 2019). In the GDD sample, we also identified *Corynebacterium* sp. and *Actinomyces* sp., which have been previously found in samples from Gobi and Sahara deserts (Griffin, 2007; Hagiwara et al., 2020). These microorganisms are generally considered to be of low pathogenicity so they pose a low human health risk, but research has shown that some strains may behave as opportunistic pathogens (Griffin, 2007).

The differences in recognized bacterial communities between ATD and GDD samples may reflect geographic differences in microorganism populations, and may also stem from the differences in particle chemistry and/or mineralogy, which are properties that can influence the distribution and availability of nutrients necessary for bacterial growth (Uroz et al., 2015; Witt et al., 2017). These properties are influenced by weathering, which alters nutrient availability and can form mineral phases that are not present in the fresh, source material (Kelly et al., 2010). We observed clear differences in the dominant mineral phases and particle surface features of our samples (Figures 2 and 4) that may have an influence on the type of associated microorganisms. The nano‐sized clay minerals covering the surfaces of GDD particles (Figure 3), as well as the presumed higher surface area of this sample as a result, may play a role in higher microbial load and diversity, as clay content is typically associated with greater microbial biomass in soil studies (Wardle, 1992).

The absence of microorganisms on SVA was not surprising since freshly erupted volcanic ash is expected to be free from bacterial or fungal contamination due to eruption temperatures and the formation of new particle surfaces (Forbes et al., 2003). However, Dawrs et al. (2024) showed recovery of respiratory‐relevant mycobacteria from volcanic ash from an eruption where abundant pre‐existing edifice material was incorporated, and other studies have demonstrated that freshly deposited volcanic ash is a favorable substrate for diverse bacterial colonization (Kelly et al., 2011; Kerfahi et al., 2017; Witt et al., 2017). This suggests that “aged” volcanic ash could contain microorganisms, similar to desert dust, that could be transported long‐distance by resuspension of volcanic deposits (Meinander et al., 2022; Đorđević et al., 2019). While outside the context of hazards that pose an immediate health concern from ashfall during an eruption (Horwell & Baxter, 2006; Stewart et al., 2022), the health hazards of ash resuspension events have garnered increasing attention (Arnalds et al., 2016; Forte et al., 2018; Hadley et al., 2004), including on St. Vincent (Horwell et al., 2024), and the presence of adhered pathogens may be a relevant consideration.

### 4.3 Particle Surface Reactivity

In this study, we assessed one facet of particle surface reactivity by measuring their OP. We determined the OP of investigated geogenic particle samples by conducting two commonly used acellular methods, the dithiothreitol (DTT) and ascorbic acid (AA) depletion assays. The principle of both assays is to measure the ability of particles or particle‐bound components to oxidize (and hence deplete) the target substrates, which are proxies for cellular reductants (DTT) or antioxidants (AA).

Overall, all of the measured OP values in this study (Figure 5) could be considered low when compared with those reported in other studies and for other PM in ambient urban environment (Q. Liu et al., 2020; S. Weber et al., 2021). Our OP values measured by DTT assay were ≤30 nmol/min/mg, which is in agreement with values measured during dust events in some areas (Farahani et al., 2022), but these are typically lower than OP_DTT_ for anthropogenic PM sampled in industrial and urban areas which range between 30 and 50 nmol/min/mg or more (Farahani et al., 2022; Q. Liu et al., 2020). The literature demonstrates that the OP of mineral dust is consistently lower than the OP of anthropogenic pollutants, despite the OP of individual particle types being rarely reported (Sauvain et al., 2021; Uzu et al., 2011). It is more common to analyze ambient air PM filters collected during dust events and compare their OP to that of PM sampled during non‐dust periods (e.g., An et al., 2022; Chirizzi et al., 2017; Farahani et al., 2022; Lovett et al., 2018; Nishita‐Hara et al., 2023). The latter largely corresponds to PM from anthropogenic sources in the urban areas, and the former still contains PM from these sources in addition to geogenic PM. Even still, these studies found that PM sampled during dust events have a lower OP than PM sampled outside of dust periods, with overall low intrinsic OP per mass of geogenic particles in comparison to urban (anthropogenic) PM.

Our results demonstrate that there are differences in measured sample OP activities between the two assays, with OP_AA_ being overall higher than OP_DTT_ (Figure 5). This difference is likely due to the different sensitivities of these tests to different particle components. It has been reported that DTT is in general more responsive to organic species and, to a lesser extent, transition metals to which AA is more sensitive to (Bates et al., 2019; Fang et al., 2016). Our results thus suggest the involvement of metals in the measured sample OP and support the usefulness of doing complementary OP tests that can give different perspectives on similar biological processes (Calas et al., 2018).

We found that volcanic ash exhibits higher OP than desert dust samples, which is consistent for both assays (Figure 5). The processes behind this result are challenging to interpret based on the state of the art. Yet, from our data on physicochemical properties, we hypothesize that the differences in measured sample OPs could be associated with two sample characteristics.

First is iron content. The amount of bulk iron (as Fe_2_O_3_, measured by ICP‐AES) is higher for the volcanic ash (8.0 wt.%; Table 2) compared to desert dust samples (2.6–3.5 wt.%). The ability of volcanic ash surfaces to generate hydroxyl radicals has been used in the literature as a proxy to surface reactivity (Damby et al., 2017; Horwell et al., 2003, 2007, 2017). Findings of a study investigating ash from Eyjafjallajökull volcano, Iceland, with AA assay, showed that ash which generates low amounts of hydroxyl radicals (<0.5 μmol m^−2^) shows no oxidative activity (Horwell et al., 2013), but the corollary (i.e., ash particles generating high amounts of hydroxyl radicals showing high oxidative activity) has not yet been demonstrated. Free radical generation from the ash surface (via the iron‐based Fenton reaction) is thought to potentially, but not exclusively, result from the presence and coordination of iron on freshly fractured ash surfaces (Horwell et al., 2007, 2017). Yet, it has been shown that hydroxyl radical generation and bulk chemical composition of ash is not always correlated (Horwell et al., 2007, 2017). Here, we did not characterize the release of soluble iron (as done by Horwell et al. (2007)) or measure other implicated transition metals; however, it is likely that the fresh SVA contains higher concentrations of water‐ or lung fluid‐soluble elements (Stewart et al., 2020; Tomašek, Damby, Stewart, et al., 2021) than the desert dusts because water‐soluble transition metals are expected to be dissolved from the surfaces of mineral dust over time (Shi et al., 2012). Earlier findings showed that freshly erupted ash is more prone to generate hydroxyl radicals than aged, weathered ash (Damby et al., 2017; Horwell et al., 2003), which may show a so‐far unaddressed likeness to desert dust. It is important to stress that most of this past knowledge on ash surface reactivity is based on studies using electron paramagnetic resonance spectroscopy and mostly on bulk samples rather than on a PM_10_ fraction (Horwell et al., 2007, 2013, 2017). Considering the differences in the approaches used in the literature compared to the approach used in our study, the comparison between the OP of SVA with the limited data reported for ash from other volcanoes must be done with caution. Based on the intermediate magmatic composition of SVA (Table 2, Section 4.1), we can assume that it is probably less potent than ash from basaltic composition (iron‐rich, crystalline silica‐poor ash), though this cannot be confirmed at this time.

A second plausible explanation for lower OP in desert dust is that it is affected by the presence of bacteria in these samples (Table 3). It has been shown that microorganisms have the ability to modulate the OP of chemicals present in atmospheric PM, depending on their type and their concentration (Samake et al., 2017). The OP values observed in our study for desert dust samples (Figure 5a) are consistent with the findings of a recent study that investigated OP_DTT_ of desert dust reference materials, including ATD (ISO 12103‐1, A2 Fine Test Dust) and Gobi Kosa dust (NIES CRM No. 30), which found that ATD has a higher OP_DTT_ than their Gobi Desert dust reference sample (Nishita‐Hara et al., 2023).

### 4.4 Bioreactivity of Geogenic Particles

Particles that enter the respiratory system deposit throughout the airways and in different parts of the lung, and are therefore in contact with different types of lung cells (Kooter et al., 2013; Schlinkert et al., 2015). Accordingly, we investigated the responses in alveolar (A549) and bronchial (BEAS‐2B) lung epithelium models and we hypothesized that, under similar conditions of exposure, the responses to particle exposures may differ. Since A549 cells are derived from a lung tissue of a patient with lung cancer, it has to be noted that the responses observed in A549 cells may not fully reflect those of healthy lung tissue. They were used due to their similarity to alveolar type II cells, which have an important role in lung physiology, as well as for being a well characterized cell line (Barosova et al., 2021). Using the BEAS‐2B cells in parallel allowed us to get a broader physiologically relevant insight into respiratory epithelial responses.

As evidenced by low levels of LDH release (Figure 6), our geogenic particle treatments resulted in minimal cytotoxicity to both cell types. The exposures did result in detectable amounts of IL‐6 and IL‐8 pro‐inflammatory cytokines in both cell models and at all treatment concentrations (Figure 7). This is in agreement with the reported ability of desert dust and volcanic ash to induce the release of pro‐inflammatory mediators in respiratory epithelial cells (Damby et al., 2016; Eychenne et al., 2022; Fussell & Kelly, 2021). However, the amounts of released cytokine in most cases did not differ significantly (*p* > 0.05) from unexposed cells (Figures 7 and 8). Such limited cellular effects, contrary to some reported in the literature, may be related to the particle doses used in this study, which are much lower (0.1 and 10 μg/cm^2^) than those tested in other in vitro monoculture studies (on submerged cultures) ranging between 25 and 200 μg/cm^2^ (Bredeck, Busch, et al., 2023; Eychenne et al., 2022). The only significant increase (*p* < 0.05) was seen for the higher dose of GDD (10 μg/cm^2^) in BEAS‐2B cells for TNF‐α and nearly significant for IL‐6 (Figures 8a and 8d). Overall, this exposure is clearly the most potent inducer of a pro‐inflammatory response in both cell lines and across measured markers, suggesting that GDD is more reactive than volcanic ash and the other desert dust sample tested here (ATD). We can also note variabilities in the pro‐inflammatory response between experiments (Figures 7 and 8). Given the heterogeneity of mineral dust samples in terms of morphology, mineralogical content, surface texture and chemistry, variability in biological responses between experiments, and even more between different studies, should be expected.

Our observations on bioreactivity of GDD are consistent with studies investigating the effects of dust from the Gobi Desert in BEAS‐2B cells, which generally agree that it can induce release of pro‐inflammatory cytokines IL‐6 and IL‐8 (Honda et al., 2014; Shin et al., 2013; B. Wang et al., 2016). Our findings also confirm variable inflammatory potencies between different desert dust samples (Figures 7 and 8). Generally, in vitro studies (which are less numerous than in vivo studies) report variable toxic potencies of desert dust depending on the source regions (Fussell & Kelly, 2021). Because most of the studies examine the cellular effects of ambient air PM collected in periods of dust storm events, which can contain an important background of urban pollutants, it is difficult to infer bioreactivity of a given desert dust sample based on the reported toxicity of samples from other deserts (Bredeck, Busch, et al., 2023). This is the first time that ash from the 2021 eruption of La Soufrière volcano was analyzed for its potential toxicity and complements work done during the eruption on its potential respiratory hazard (Horwell et al., 2024). This is why we can only state that its bioreactivity is in agreement with what is generally observed in *in vitro* studies of volcanic ash of similar composition (i.e., andesite; e.g., Damby et al., 2016, 2018; Eychenne et al., 2022; Tomašek et al., 2018), but we cannot infer whether it is more potent than ash from other phases of this eruption or ash from other volcanoes. It is worth noting that geogenic particles are in general considered to be less biologically potent than anthropogenic particles (i.e., combustion‐derived). This is mainly because they tend to have lower contents of organic compounds and metals, which are known inflammatory stimulants (Donaldson et al., 2005). In the literature, there is a limited number of studies directly comparing bioreactivity of various particle types but this notion is confirmed in a few in vitro studies where anthropogenic particles represented by NIST (National Institute of Standards and Technology) materials triggered stronger pro‐inflammatory response in lung cell models than mineral dust or volcanic ash (Ghio et al., 2014; Grytting et al., 2022; Tomašek et al., 2016).

Despite the overall low‐level pro‐inflammatory response, our results demonstrate the acute pro‐inflammatory potential of geogenic particles in the lung epithelium that could potentially aggravate existing airway inflammation by initiating recruitment and/or activation of different immune cells (e.g., macrophages, neutrophils). Through potential impairment of immune responses, these particles could also render individuals more susceptible and/or exacerbate the response to respiratory infections (Clifford et al., 2015; Williams et al., 2023). In this study, we focused on the potential effects of acute exposures and with our current in vitro ALI approach it was not possible to simulate chronic exposure conditions. The possible effects of long‐term exposure to geogenic particles therefore cannot be directly deduced from our experiments. However, considering the observed pro‐inflammatory potency and what is known from the literature, we can hypothesize that repeated exposure to sustained even low‐level doses could potentially lead to cumulative and increased biological effects (Chen & Hoek, 2020; Cherrie et al., 2013), especially in the presence of other volcanic or urban (anthropogenic) particle types. This hypothesis requires further research.

Interestingly, we observed that the effects of particle exposures in BEAS‐2B cells appear to be more pronounced compared to A549 cells based on the mean LDH release (Figure 6) and mean cytokine level (Figure 8) fold change above background. Moreover, BEAS‐2B cells released IL‐1β and TNF‐α whereas their levels were below detection limits in A549 cells. This difference in response suggests that bronchial epithelial cells were more sensitive and responsive to particle exposures, which could have important implications for understanding the respiratory hazard posed by geogenic particles. In the environment, geogenic particles are generally present in the PM_10_ fraction (Williams et al., 2017), which can deposit throughout the whole respiratory tract, and especially in the upper respiratory tract and bronchial region, where they can induce adverse health effects (Heyder et al., 1986; Nishita‐Hara et al., 2023). Importantly, the bronchiolar small airways play a major role in the development of airway diseases, such as asthma and chronic obstructive pulmonary disease (Fransen & Leonard, 2022). Considering the differences in responses (and the ability to induce different cytokines) for the two cell types tested in our study and different pathologies associated with different regions in the lungs, future research efforts could give more attention to the effects that geogenic particles can induce in bronchial region of the lungs.

### 4.5 Potential Drivers of the Biological Response

The extensive characterization of samples in this study helps to inform which particle components or properties may be implicated in their bioreactivity and associated responses in lung epithelium models upon particle exposures. We assessed some of the common respiratory toxicity‐associated properties including particle morphology, mineralogy, OP, and presence of microorganisms. It has to be noted that we assessed the whole sample bioreactivity, hence recording the effects of particle mixtures rather than analyzing the specific contribution of individual particle components to observed bioreactivity.

Particle size and shape were comparable across the desert dust samples, while the volcanic ash sample was more irregular (Figure 1), and no fiber‐like particles were observed that are considered a respiratory concern (Riediker et al., 2019), which suggests that these properties would have little impact on differences in the cellular responses.

In the SVA sample, we identified the presence of quartz and cristobalite (Supplementary file 1 in Supporting Information S1), with total crystalline silica comprising 6 area % of the sub‐10 μm fraction. This aligns with recent work that quantified cristobalite in bulk (i.e., not size separated) La Soufrière ash from the same eruption period and found low amounts (<5 wt.%) (Horwell et al., 2024). Crystalline silica minerals have been implicated in volcanic ash bioreactivity (Damby et al., 2016, 2018; Nattrass et al., 2017), but the SVA exposure resulted in relatively low cellular responses (Figure 7). Volcanic cristobalite is known to trigger variable reactivity (Damby et al., 2018; Horwell et al., 2012), which can explain the low cellular response to SVA. Similarly, a limited cellular response despite the presence of sulfates on ash surfaces (Supplementary files 1 and 3 in Supporting Information S1) is in agreement with previous findings where it has been shown that sulfates do not have an impact on ash toxicity in vitro (Tomašek et al., 2019). A similar and limited pro‐inflammatory response in cells was observed for the ATD sample at the doses presented here (Figure 7), even though its most prevalent phase is quartz (46 area %; Figure 2), another form of crystalline silica of respiratory concern which is known to stimulate release of measured cytokines (IL‐8 and IL‐1β) in lung epithelial cells (Stone et al., 2007). However, the extent and nature of the cellular responses upon exposure to quartz are also known to vary, which is attributed to variations in surface functionalities (Pavan & Fubini, 2017). Moreover, at the concentrations explored here, the response between ATD and SVA appears to be similar even though there are observable differences in the particle bulk and surface composition, and surface features (Figure 3). By comparison, the GDD sample induced a notably higher release of cytokines (Figure 7), although it is largely composed of calcite (CaCO_3_), for which there is limited evidence of toxic potential. The existing studies suggest that respirable calcite dust particles have little to no effect on epithelial cells or macrophages in vitro but that their effects may increase in co‐exposure with silica (Khaliullin et al., 2019), which would be the case for GDD (Figure 2). Overall, in our results, there is no clear link between bulk particle mineralogy and the differences in cellular responses observed among the samples.

Unique to GDD is its complex surface texture, including the presence of extensive elongate clay particles (Figure 3). Surface roughness created by these clay particles may have increased GDD's ability to interact with the cells due to an increased contact area (Duffin et al., 2002). Clay minerals are also known to be charged minerals, which could play a role in their surface reactivity. The toxicity of clay minerals has been rather extensively investigated, especially ubiquitous clays such as kaolinite and montmorillonite, but there is no definitive conclusion regarding their toxicity (Maisanaba et al., 2015; Wagner et al., 1998). Illite and vermiculite, which could be the clay phases covering the surface of GDD sample, have been less researched, but a recent study on illite found that it can be potentially more cytotoxic to lung cells than some other clays (Ramirez Diaz, 2021).

Oxidative potential is associated with the potential of particles to act as catalysts in the formation of reactive oxygen species (ROS), either through redox‐active species (such as transition metals) that can be released from mineral surfaces upon contact with lung fluid or directly through surfaces of insoluble metal‐containing minerals. Hence, measurement of OP is considered biologically relevant and regarded as an indicator of particle biological activity associated with an oxidative stress response, which has been implicated as a mechanism causing mineral dust‐induced inflammatory responses (Bates et al., 2019; Øvrevik, 2019). Although some studies associate OP assay results with in vitro endpoints (Leni et al., 2020; Uzu et al., 2011), in general, it seems there is no consistent link between biological response, in terms of cytotoxicity and inflammation, and measured particle OP (Øvrevik, 2019; Øvrevik et al., 2006). This is the case in our results, where OP determined by both DTT and AA assays does not correlate with the cellular release of cytokines. In fact, GDD, which is the sample with the highest levels of released IL‐6 and IL‐8, shows the lowest OP_DTT_ (Figure 5a), suggesting that the oxidizing capacity of PM is not the main driver of the observed cellular effects. This could mean that GDD induced release of cytokines through pathway(s) independent of oxidative stress (e.g., Bredeck, Busch, et al., 2023) and that the response is not driven by ROS. Considering the lack of correlation and overall low‐level OP measured in our samples, comparable to ambient background values (S. Weber et al., 2021), it may also be that OP is not a sensitive enough metric for bioreactivity of geogenic particles, at least not at the concentrations used in our study.

The presence of bacteria on both desert dust samples suggests that a biological component may play a role in the cellular responses observed. Indeed, the induction of pro‐inflammatory responses by dust particles has been previously linked with the presence of bacteria (Fussell & Kelly, 2021). Moreover, the species identified in the samples (*Bacillus* sp. and *Actinomyces* sp.) are rod‐shaped gram‐positive bacteria, and gram‐positive bacteria were previously found to stimulate release of IL‐6 and IL‐8 in A549 cells (Larsson et al., 1999). More pronounced effects for GDD in comparison to ATD, which contains *Bacillus* sp. like GDD but also *Actinomyces* sp., might be due to higher overall bacterial count (CFU) in the GDD sample (Table 3) but also a different potency of bacteria to stimulate cytokine production. It has been shown that whole bacteria as well as only their soluble products and/or outer membrane components are capable of inducing cytokine release (Larsson et al., 1999), as is the case for LPS used in our experiments. It is also possible that exposure to bacteria in GDD triggered the release of pro‐inflammatory cytokines and rendered the cells more susceptible to other particle components (Damby et al., 2018; Rodríguez‐Cotto et al., 2013), which perhaps did not occur for the ATD sample. The exact potency of associated microorganisms cannot be confirmed at this time, since we did not do a comparison of pristine dust samples with heat‐treated samples. This would remove endotoxins and, in this way, possibly modify and/or diminish the cellular response (Bredeck, Busch, et al., 2023) thus revealing the “true” toxicity of particles. Without such additional testing including particle endotoxin levels, which was not possible at the time of study, we cannot comment further on the influence and contribution of identified microorganisms to the observed pro‐inflammatory responses.

Considering that GDD is an aeolian sample, it is possible that this sample contains other adsorbed compounds or particles from other sources (e.g., anthropogenic) that we did not test for in our investigation. Notably, the presence of organic compounds was shown to enhance the toxicity of dust (Fussell & Kelly, 2021). This will be important to consider in the future as studies suggest that combined exposure to PM may induce increased effects compared with the individual compounds (e.g., Grytting et al., 2022; Z. Wang et al., 2019; Tomašek et al., 2016).

Finally, assessment of cellular signaling pathways underlying the observed cytokine release would help shed the light on key drivers of cellular response to GDD. For example, endotoxin from gram‐negative bacteria in desert dust has been implicated in activation of toll‐like receptor 4 (TLR‐4) pathway, which leads to production of cytokines and aggravation of allergic lung inflammation (Fussell & Kelly, 2021). Another pathway implicated in pro‐inflammatory response incited by desert dust is the NLRP3 inflammasome (Bredeck, Busch, et al., 2023, Bredeck, Dobner, et al., 2023), which can also be activated by volcanic ash stimulation (Damby et al., 2018). Such assessments were beyond the scope of the present study and remain to be addressed in the future.

## 5 Conclusions

This study provides the first systematic insight into the differences between freshly erupted volcanic ash and desert dust in terms of their physicochemical properties and bioreactivity. This was achieved through extensive particle characterization and toxicological appraisal in human lung airway models in vitro, with conductive airways represented by BEAS‐2B bronchial epithelial cells and the respiratory zone by A549 alveolar epithelial cells.

Our results reveal intrinsic differences in the physicochemical properties of geogenic particles and provide evidence for variability in their biological effects. We showed that both volcanic ash and desert dust induce biological effects to some extent, but that their potency differs. Desert dust from the Gobi Desert was found to be more bioreactive than freshly erupted volcanic ash from La Soufrière volcano, St. Vincent and a standardized test dust from the Arizona Desert. We hypothesize that the higher pro‐inflammatory response for the Gobi Desert dust sample is linked to the presence of microorganisms (bacteria) and/or nanoscale, elongated silicate minerals (clay such as illite or vermiculite) on the particle surfaces. In agreement with some past studies, our results show that analyzing only one particle property (e.g., OP, crystalline silica content) is not sufficient to predict particle bioreactivity for mixed‐mineral dusts and that biological testing may be necessary.

Considering the differences in cellular response to geogenic particle treatments where effects in bronchial cells appeared to be more pronounced in comparison to their effects in alveolar cells, our findings further suggest that the testing of geogenic particle respiratory effects should be considered in different lung models.

Globally, populations are at an increasing risk of frequent exposure to geogenic particles due to increases in dust emissions resulting from climate change and desertification associated with changes in land use. The observations of this study expand on the rather limited knowledge base from in vitro studies on desert dust and volcanic ash toxicity, thereby advancing the understanding of the hazard posed by different geogenic particles. Thus, these data provide information that can be used alongside appropriate exposure information in respiratory health risk assessments.

## Conflict of Interest

The authors declare no conflicts of interest relevant to this study.

## Supporting information

Supporting Information S1

## Data Availability

The data sets used and/or analyzed during the current study are available at Tomašek (2024).
