# Supplementary material for: Physicochemical Properties and Bioreactivity of Sub‐10 μm Geogenic Particles: Comparison of Volcanic Ash and Desert Dust
Source: Geohealth. 2025 Jan 8;9(1):e2024GH001171. doi: 10.1029/2024GH001171 (PMC11711107; doi:10.1029/2024GH001171)

Physicochemical properties and bioreactivity of sub-10 µm geogenic particles: comparison of volcanic ash and desert dust

Ines Tomašek[1,2,3*], Julia Eychenne[1,2], David E. Damby[4], Adrian Hornby[5,6], Manolis N. Romanias[7], Severine Moune[1], Gaëlle Uzu[8], Federica Schiavi[1], Maeva Dole[1], Emmanuel Gardès[1], Mickael Laumonier[1], Clara Gorce[1], Régine Minet-Quinard[2,9], Julie Durif[9], Corinne Belville[2], Ousmane Traoré[10,11], Loïc Blanchon[2†], Vincent Sapin[2,9†]

[1]Laboratoire Magmas et Volcans (LMV), CNRS, IRD, OPGC, Université Clermont Auvergne, France.

[2]Institute of Genetic Reproduction and Development (iGReD), Translational Approach to Epithelial Injury and Repair Team, CNRS, INSERM, Université Clermont Auvergne, France.

[3]Istituto Nazionale di Geofisica e Vulcanologia (INGV), Osservatorio Etneo, Catania, Italy.

[4]Volcano Science Center, U.S. Geological Survey (USGS), USA.

[5]Department of Earth and Atmospheric Sciences, Cornell University, USA.

[6]Department of Cellular and Molecular Biology, School of Medicine, University of Texas at Tyler, USA.

[7]Institut Mines-Télécom (IMT) Nord Europe, Centre for Energy and Environment, Université Lille, France.

[8]Université Grenoble Alpes, IRD, CNRS, INRAE, INP-G, IGE (UMR 5001), France.

[9]Centre Hospitalier Universitaire (CHU) Clermont-Ferrand, Biochemistry and Molecular Genetics Department, France.

[10]Centre Hospitalier Universitaire (CHU) Clermont-Ferrand, Infection Control Department, France.

[11]Laboratoire Microorganismes: Génome Environnement (LMGE), UMR, CNRS, Université Clermont Auvergne, France.

[†]These authors share last authorship.

[*]Correspondence:

Ines Tomašek (ines.tomasek@uca.fr)

Supplementary material

Content:

- Supplementary Figures 1 and 2
- Supplementary Tables S1 and S2
- Supplementary files 1, 2 and 3
    - **Supplementary file 1:** Representative Raman spectra for each of the phase identified in the ATD, GDD and SVA samples.

- **Supplementary file 2:** Data and results of the phase reconstruction using the ImageJ macro "EDS pie": raw EDS maps and corresponding reconstructed phase maps for each independent user.
- **Supplementary file 3:** SE images, EDS maps and EDS spectra for example surface species observed on representative ATD, GDD and SVA particles.

Supplementary figures:

Figure S1: Flow chart describing the ImageJ macro "EDS pie" for phase quantification using EDS single-element maps.

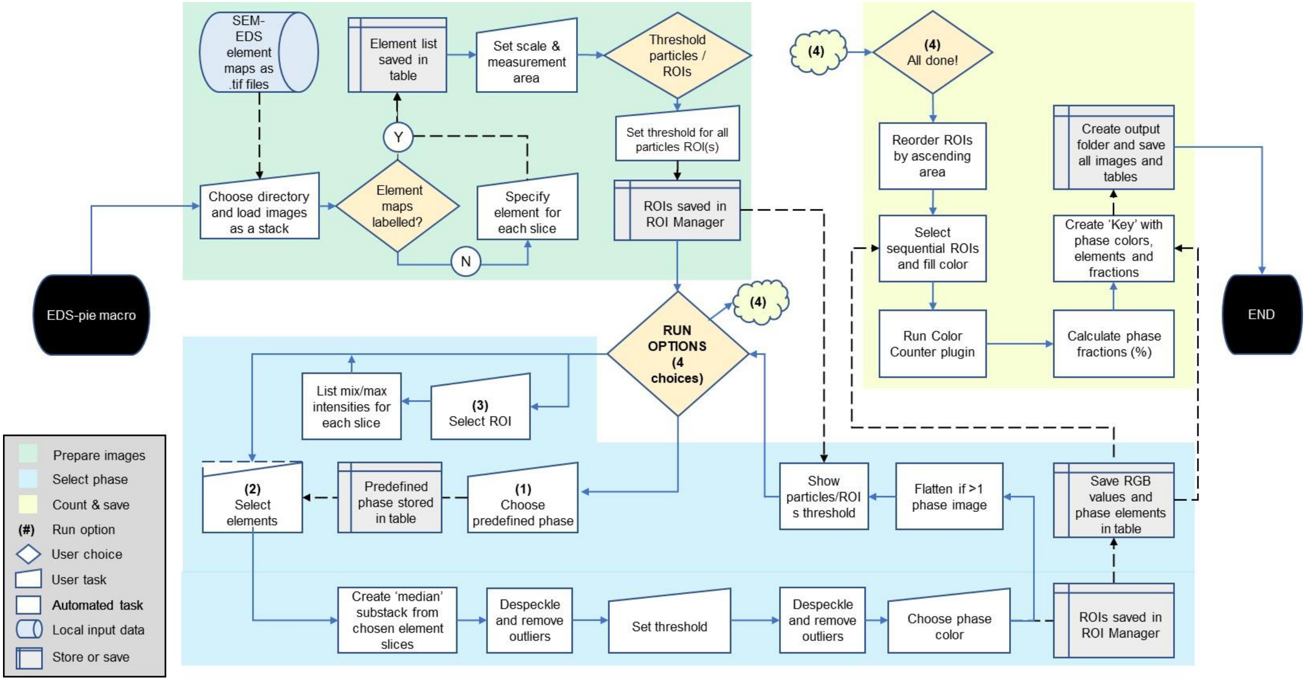

Figure S2: Dose-response in alveolar epithelial cells. **(a)** Cytotoxicity towards A549 of the respirable Arizona test dust (ATD), Gobi Desert dust (GDD) and St. Vincent volcanic ash (SVA) at different doses (0.1, 10, 50 or 100 µg/cm^2) after 24 h exposure at an ALI, measured by lactate dehydrogenase (LDH) activity. LDH release is expressed as a fold change relative to untreated cells (serum-free cell culture medium only) measured from three independent experiments (N=3). Quartz particles (Q; Min-U-Sil < 10 µm) at 100 µg/cm^2 in serum-free aCCM and Triton X-100 (TX) at 0.2% in phosphate buffered saline acted as the positive assay controls. **(b)** and **(c)** Pro-inflammatory response in alveolar epithelial cells (A549) assessed by cytokine production following acute particle exposures at different doses (0.1, 10, 50 or 100 µg/cm^2) measured after 24 h exposure at an ALI in culture supernatants on duplicates or triplicates of three independent experiments (N=3). Protein production is presented as the cytokine concentration normalised to the total protein concentration and expressed as a fold change relative to untreated cells for **(b)** IL-6 and **(c)** IL-8. Lipopolysaccharide (LPS, from E. coli, at 10 µg/mL) and quartz particles (Q; Min-U-Sil < 10 µm) at 100 µg/cm^2 in serum-free aCCM were used as a pro-inflammatory stimulants. All data are presented as the median with range. The points are the average value of individual measurements of the replicates within an experiment.

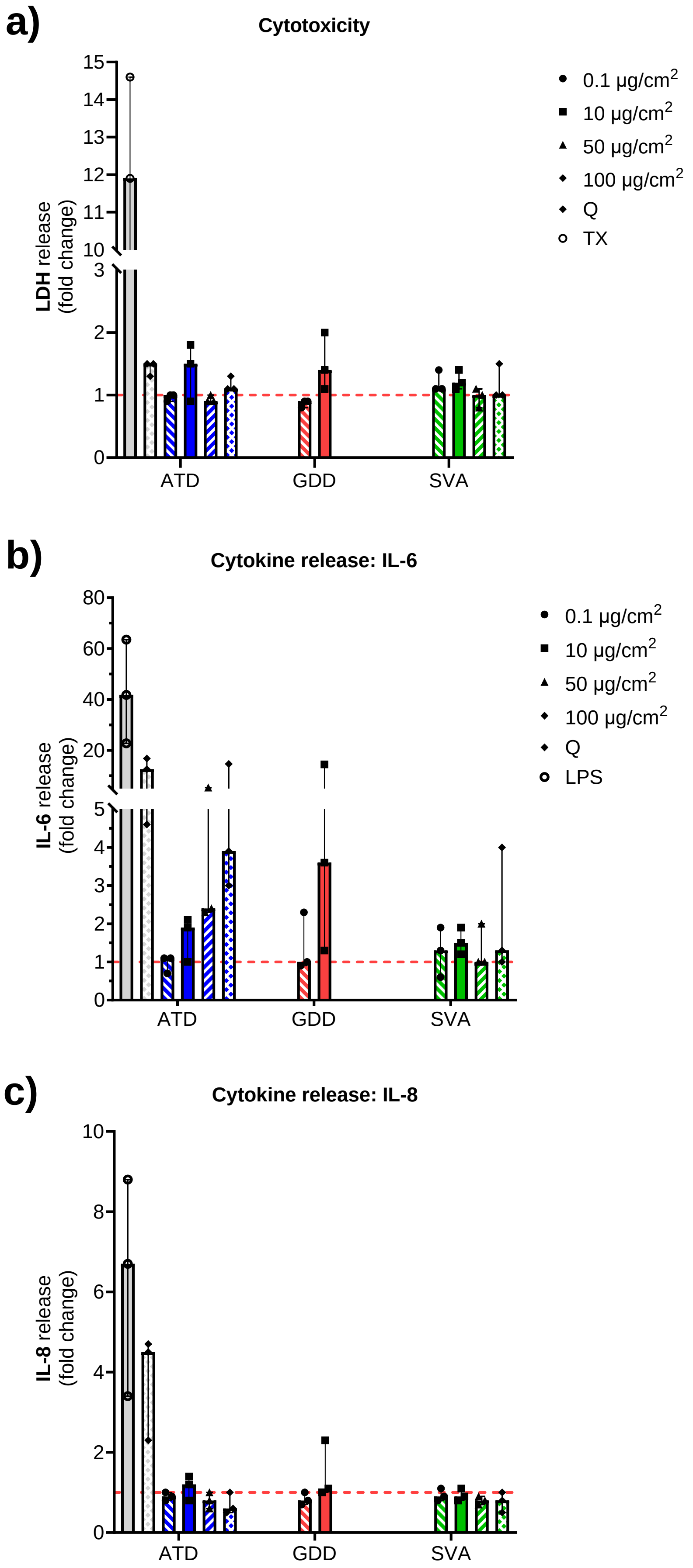
a)
Cytotoxicity
LDH release (fold change)
15
14
13
12
11
10
3
2
1
0
ATD
GDD
SVA
0.1 µg/cm2
10 µg/cm2
50 µg/cm2
100 µg/cm2
Q
TX
b)
Cytokine release: IL-6
IL-6 release (fold change)
80
60
40
20
5
4
3
2
1
0
ATD
GDD
SVA
0.1 µg/cm2
10 µg/cm2
50 µg/cm2
100 µg/cm2
Q
LPS
c)
Cytokine release: IL-8
IL-8 release (fold change)
10
8
6
4
2
0
ATD
GDD
SVA

Supplementary tables:

Table S1: Results of the phase proportion estimation by image analysis of EDS maps for the three samples using the ImageJ macro 'EDS-pie' by two independent users. For each user: elements chosen to threshold the phase and area % of the phase. Mean area % and error between the two users calculated as the difference between the area % quantified independently by the two users. For typical compositions of the phases identified below, refer to international mineral databases such as: www.mindat.org/ or https://webmineral.com/

Sample	Phase	Elements chosen for identification AH	Area% AH	Elements chosen for identification JE	Area % JE	Mean Area %	Error
**ATD**	Crystalline silica	Si	46.3	Si	46.1	46.2	0.2
	K-feldspar	Al+K+Si	10.2	K	12.2	11.2	1.9
	Ca-Na-feldspar	Al+Na	18.1	Na and Al+Ca	21.7	19.9	3.6
	Calcite	Ca	8.2	Ca	5.4	6.8	2.8
	Mg-calcite	Mg+Ca	1.8	Ca+Mg	1.7	1.8	0.1
	Mica/Clay	Mg+Fe+(Na/K+Al)	8.9	K+Mg	5.3	7.1	3.6
	Pyroxene + Olivine + Fe oxide	Fe+Mg	6.4	Mg	7.4	6.9	1.0
**GDD**	K-Feldspar	Al+K+Si	34.3	K	37.8	36.1	3.6
	Ca-Na-feldspar	Al+Na	5.4	Na	6.3	5.8	0.9
	Calcite	Ca	26.4	Ca	24.1	25.2	2.4
	Mg-calcite	Mg+Ca	3.5	Mg+Ca	3.0	3.3	0.5
	Crystalline silica	Si	11.5	Si	7.9	9.7	3.6
	Mica/Clay	Mg+Fe+(Na/K+Al)	9.5	Al+Si and Al	14.8	12.2	5.3
	Pyroxene + Olivine + Fe oxide	Fe+Mg	9.3	Mg	5.1	7.2	4.1
	S-Ti-bearing species	Ti+S	0.3	Ti	0.3	0.3	0.03
**SVA**	Ca-Na-feldspar	Al+Ca+Na	35.0	Al+Ca+Na	35.2	35.1	0.2
	Glass (dacitic + rhyolitic compositions)	K+Si	21.8	K	22.4	22.1	0.6
	Orthopyroxene + Olivine	Fe+Mg	14.6	Mg	17.6	16.1	3.0
	Clinopyroxene	Ca+Mg+Fe	6.3	Ca+Mg	6.0	6.1	0.3
	Crystalline silica	Si	6.5	Si	5.1	5.8	1.4
	Fe-Ti oxides	Fe+Ti	5.5	Fe+Ti	4.6	5.1	0.9
	Salts	S+(Cl)+(Mg)+(Ca)	9.9	S+(Cl)+(Ca)+(Al)	8.6	9.2	1.2
	Mica	K+Mg	0.4	Mg + K	0.4	0.4	0.01
SVA salts	*Anhydrite*	*Ca+S*	*4.2*		*3.2*		
	Cl-Mg salts	*Cl+Mg*	*3.3*				
	Cl-S salts	*Cl+S*	*2.3*				
	Chlorides				*3.7*		
	Pyrite and/or Fe-chloride				*0.8*		
	Natroalunite				*0.9*		

Table S2: Semi-quantitative composition in normalized oxide wt.%, of the calcite core and silicate rim of the GDD particle shown in Fig. 4, calculated from the spectra presented in Fig. 4c. Given that the samples were carbon coated, the carbon content is not quantified.

	SiO_2	TiO_2	Al_2O_3	FeO	MgO	CaO	Na_2O	K_2O
**Calcite core**	0.0	0.4	3.0	0.3	0.0	96.3	0.0	0.0
**Si-rich rim**	40.6	2.0	18.7	20.0	4.3	9.4	0.0	5.0

Supplementary file 1:

Representative Raman spectra of the main mineralogical phases identified in the ATD, GDD and SVA samples.

Arizona Test Dust (ATD) mineralogical phases' raman spectra

Assessed by Raman spectroscopy on respirable particles deposited on carbon tape.

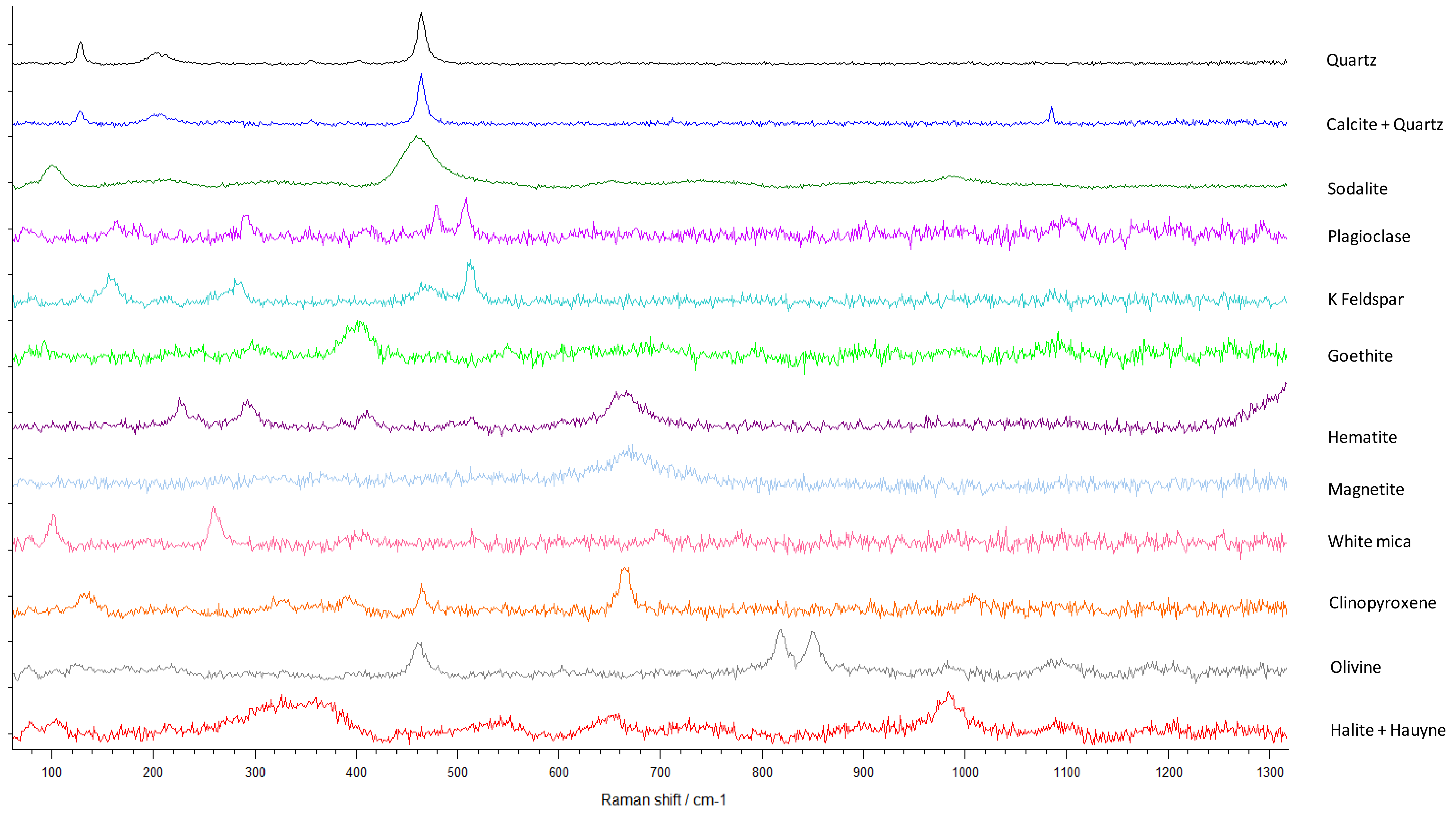
Quartz
Calcite + Quartz
Sodalite
Plagioclase
K Feldspar
Goethite
Hematite
Magnetite
White mica
Clinopyroxene
Olivine
Halite + Hauyne
100
200
300
400
500
600
700
800
900
1000
1100
1200
1300
Raman shift / cm-1

Gobi Desert Dust (GDD) mineralogical phases' raman spectra

Assessed by Raman spectroscopy on respirable particles deposited on carbon tape.

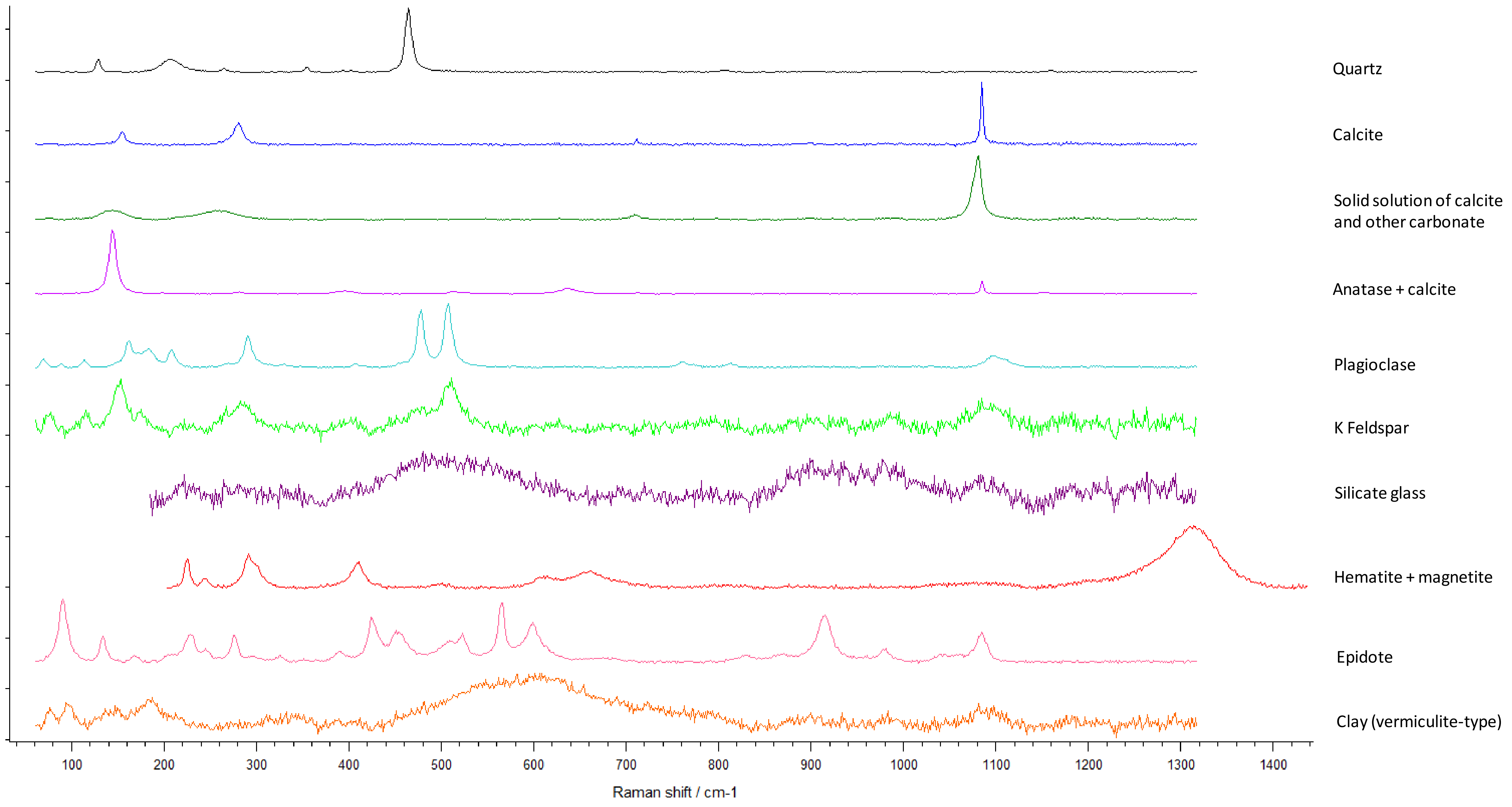
Quartz
Calcite
Solid solution of calcite and other carbonate
Anatase + calcite
Plagioclase
K Feldspar
Silicate glass
Hematite + magnetite
Epidote
Clay (vermiculite-type)
100
200
300
400
500
600
700
800
900
1000
1100
1200
1300
1400
Raman shift / cm-1

St Vincent ash (SVA) mineralogical phases' raman spectra

Assessed by Raman spectroscopy on polished mounts of particles impregnated in low viscosity epoxy resin (Logitech LTD).
* Grey stars represent peaks from resin.

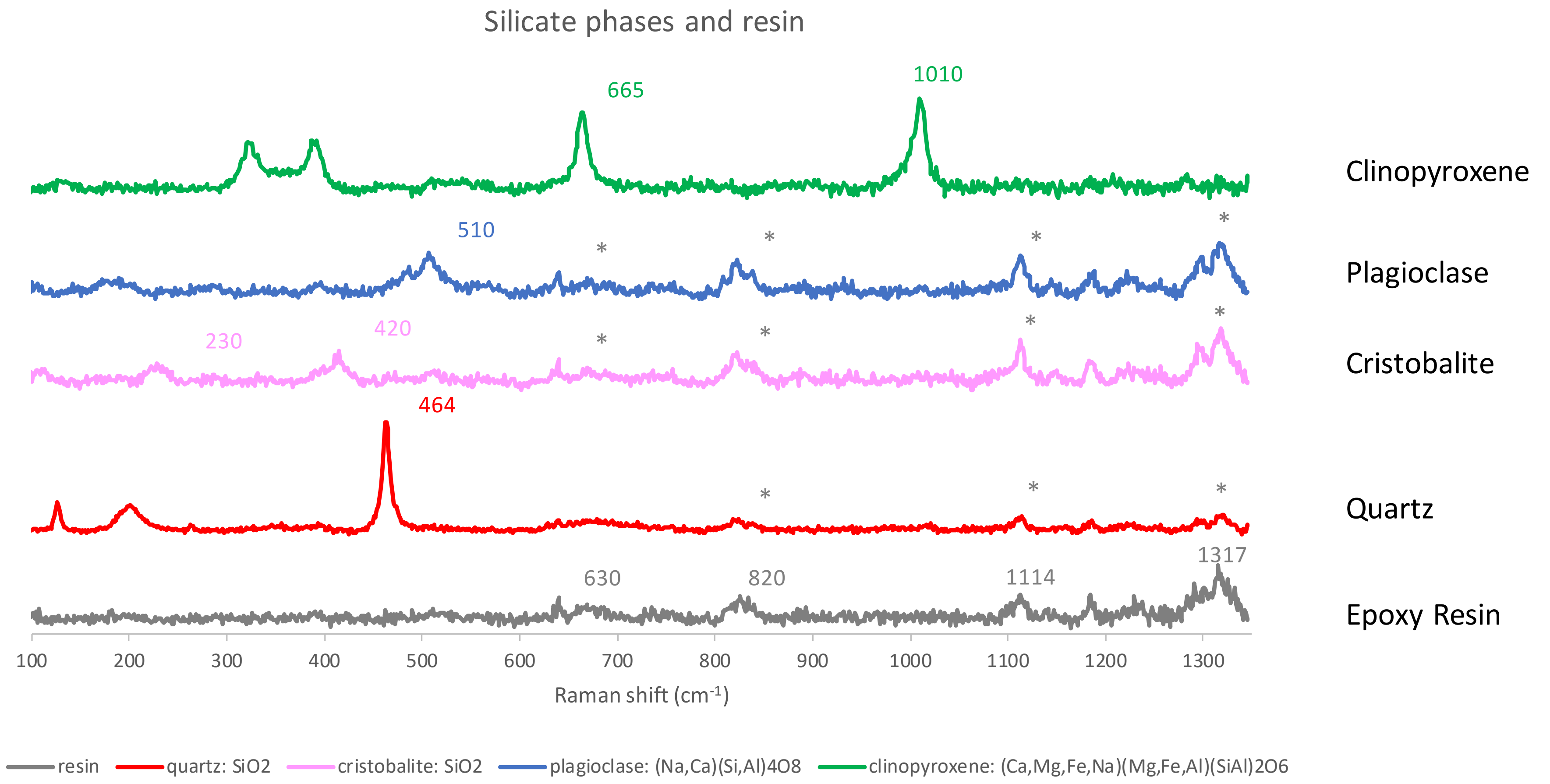

Silicate phases and resin
1010
665
Clinopyroxene
510
Plagioclase
230
420
Cristobalite
464
Quartz
630
820
1114
1317
Epoxy Resin
100 200 300 400 500 600 700 800 900 1000 1100 1200 1300
Raman shift (cm-1)
resin
quartz: SiO2
cristobalite: SiO2
plagioclase: (Na,Ca)(Si,Al)4O8
clinopyroxene: (Ca,Mg,Fe,Na)(Mg,Fe,Al)(SiAl)2O6

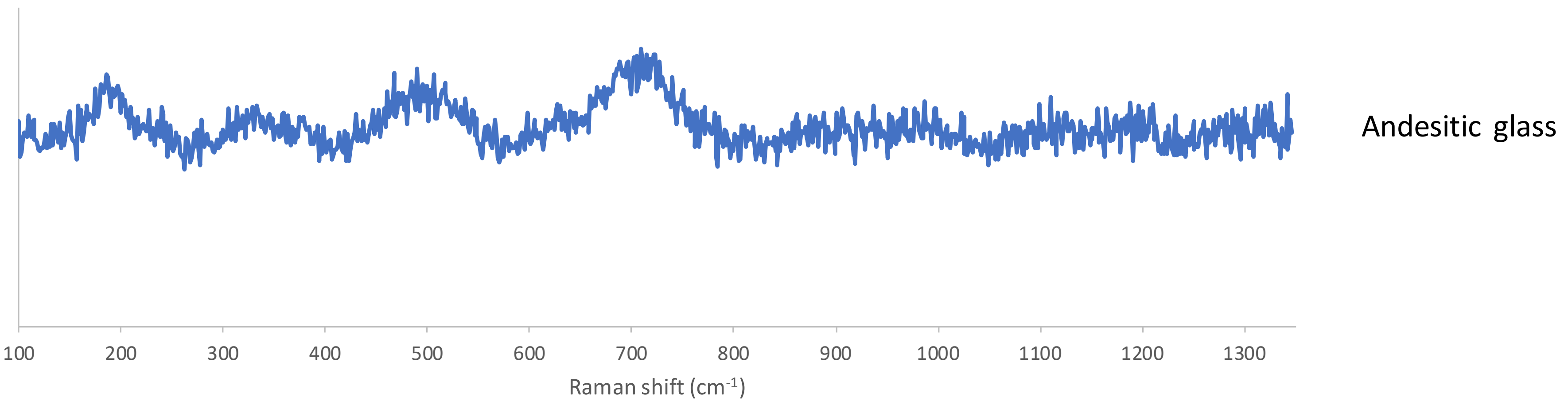

Andesitic glass
100 200 300 400 500 600 700 800 900 1000 1100 1200 1300
Raman shift (cm-1)

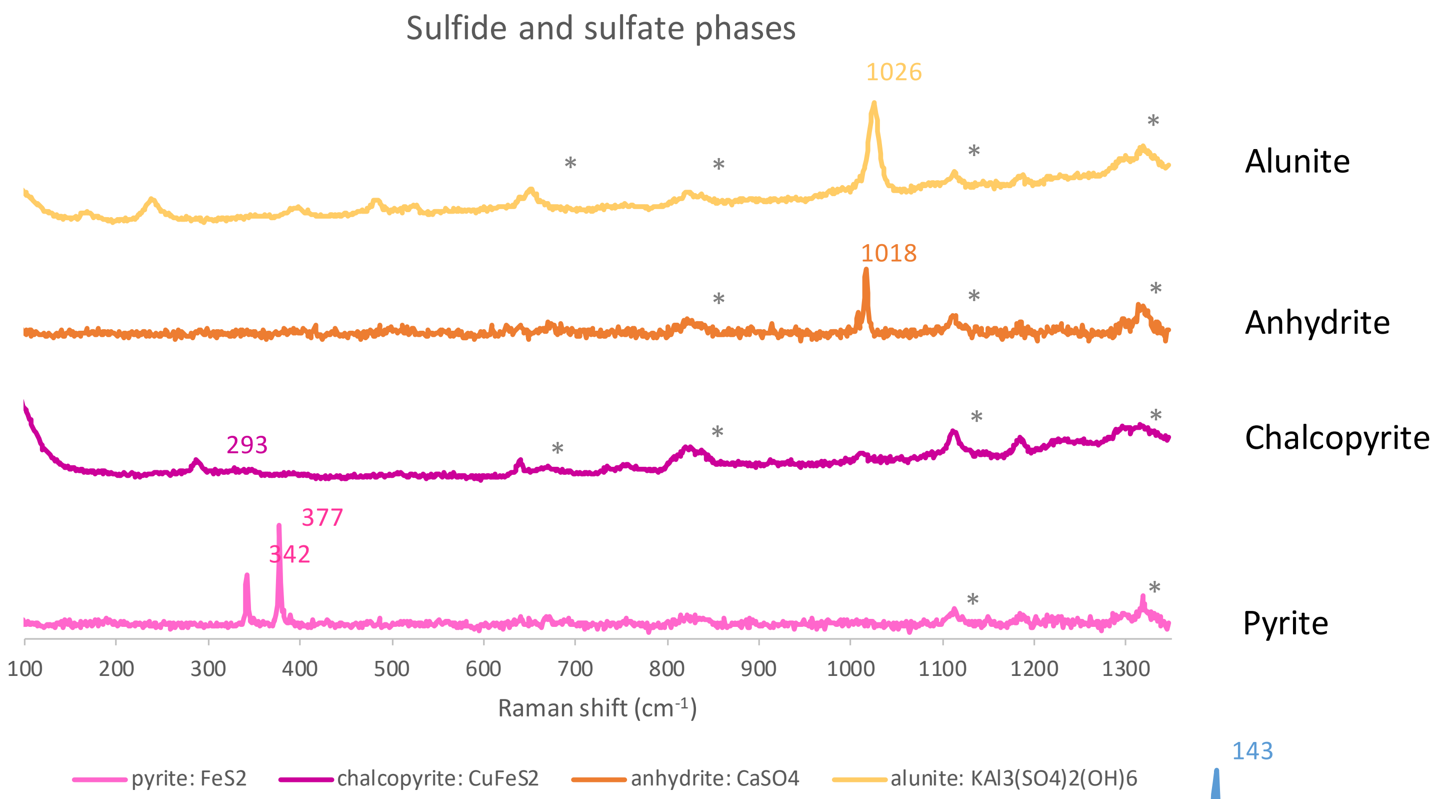
Sulfide and sulfate phases
1026
1018
293
377
342
Alunite
Anhydrite
Chalcopyrite
Pyrite
100 200 300 400 500 600 700 800 900 1000 1100 1200 1300
Raman shift (cm^{-1})
pyrite: FeS2
chalcopyrite: CuFeS2
anhydrite: CaSO4
alunite: KAl3(SO4)2(OH)6

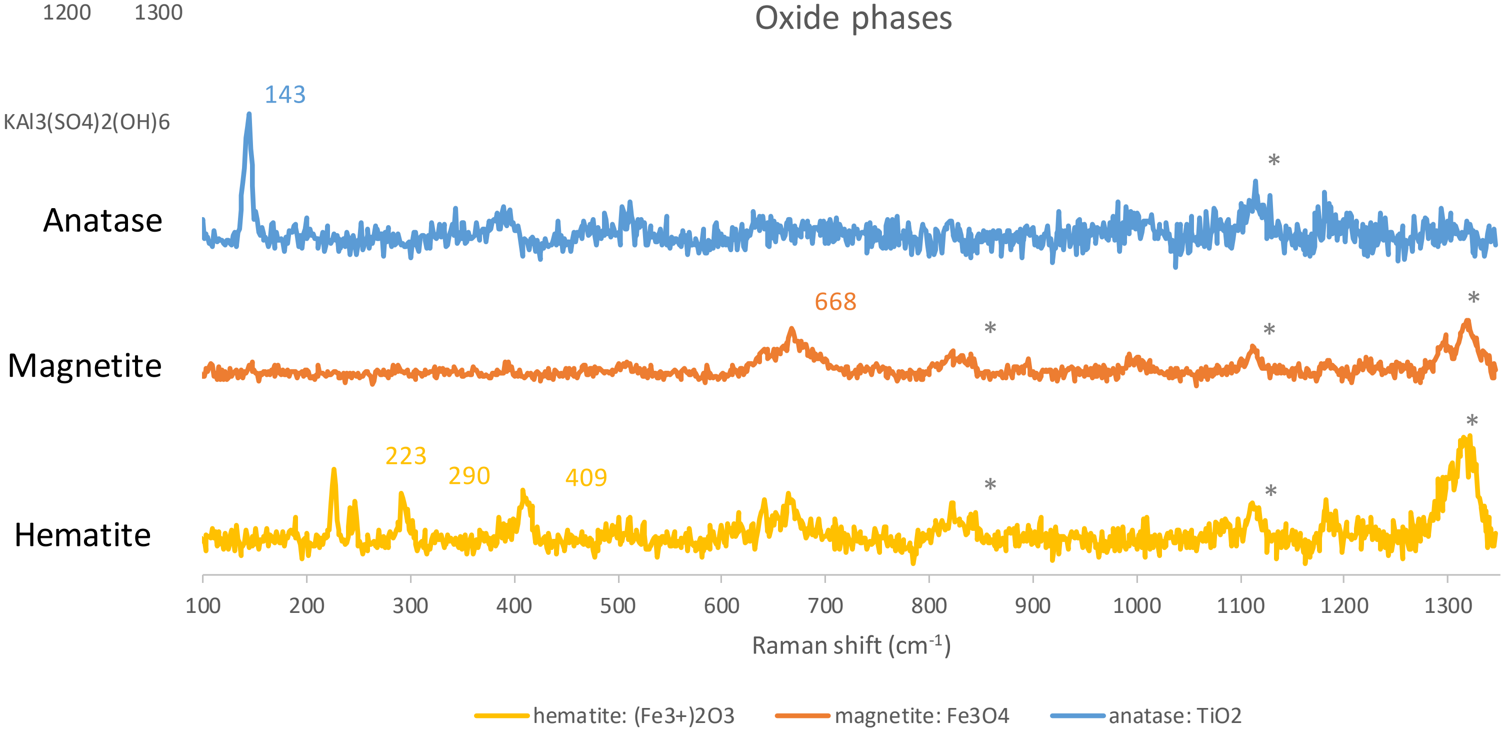
Oxide phases
143
668
223
290
409
Anatase
Magnetite
Hematite
100 200 300 400 500 600 700 800 900 1000 1100 1200 1300
Raman shift (cm^{-1})
hematite: (Fe3+)2O3
magnetite: Fe3O4
anatase: TiO2

Supplementary file 2

Data and results of the mineralogical phase reconstruction in the ATD, GDD and SVA dust samples, using the ImageJ macro “EDS pie”:

- multi-elemental EDS maps
- corresponding reconstructed phase maps for each independent user (JE and AH)

Arizona Test Dust (ATD) maps

Map 1:

Multi-elemental EDS map

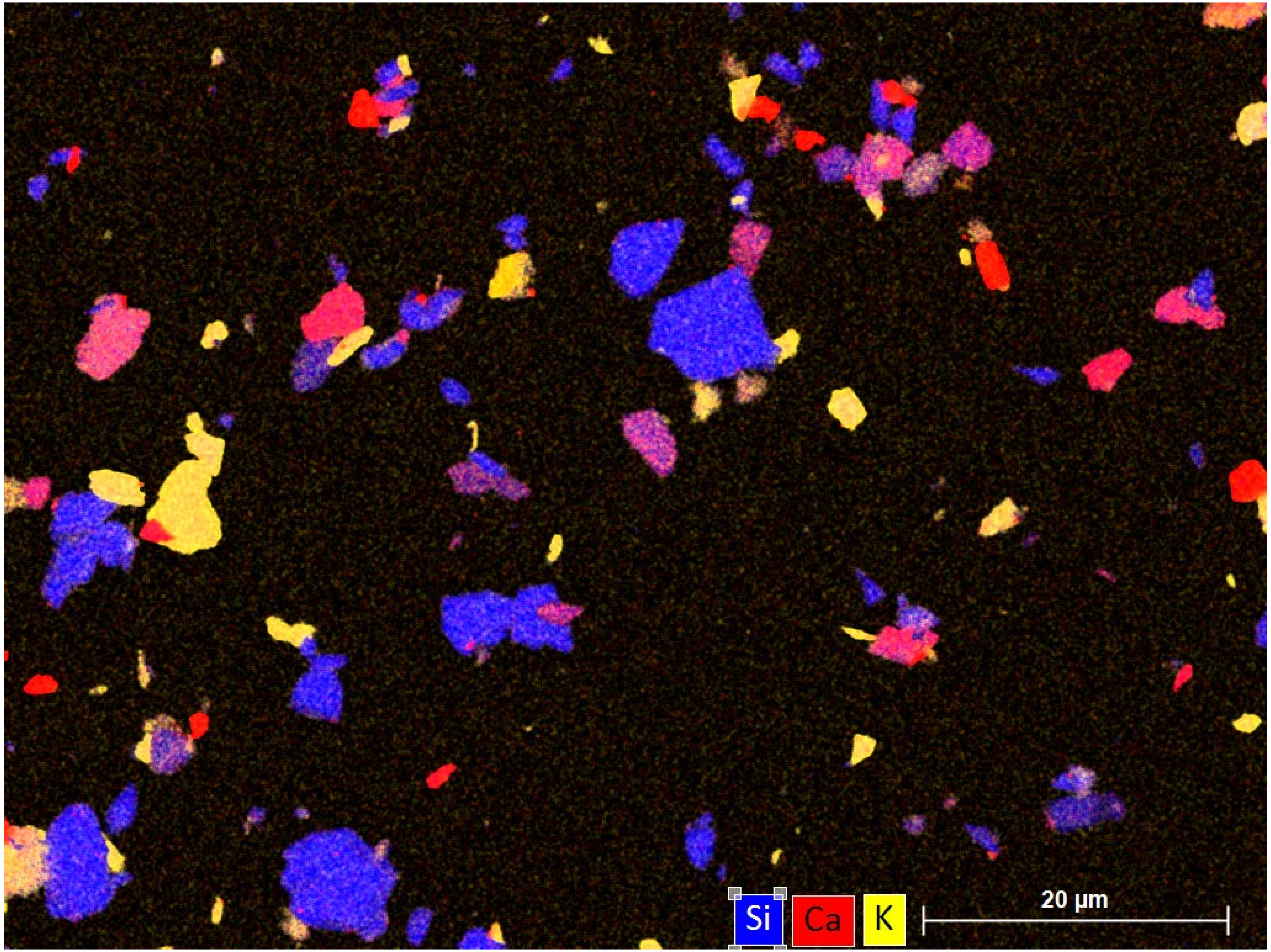

Map 1: Phase maps

Output JE

			Area % normalized to 100%
Crystalline silica (quartz)	33.2%	"Si"	43.3
Plagioclase (all compositions)	16.7%	"Na"	21.8
K-feldspar	7.49%	"K"	9.8
Pyroxene + Olivine + Fe oxide	6.53%	"Mg"	8.5
Clay/Mica	6.22%	"K"+"Mg"	8.1
Calcite	3.84%	"Ca"	5.0
Mg-calcite	1.61%	"Ca"+"Mg"	2.1
Plagio	1.14%	"Al"+"Ca"	1.5

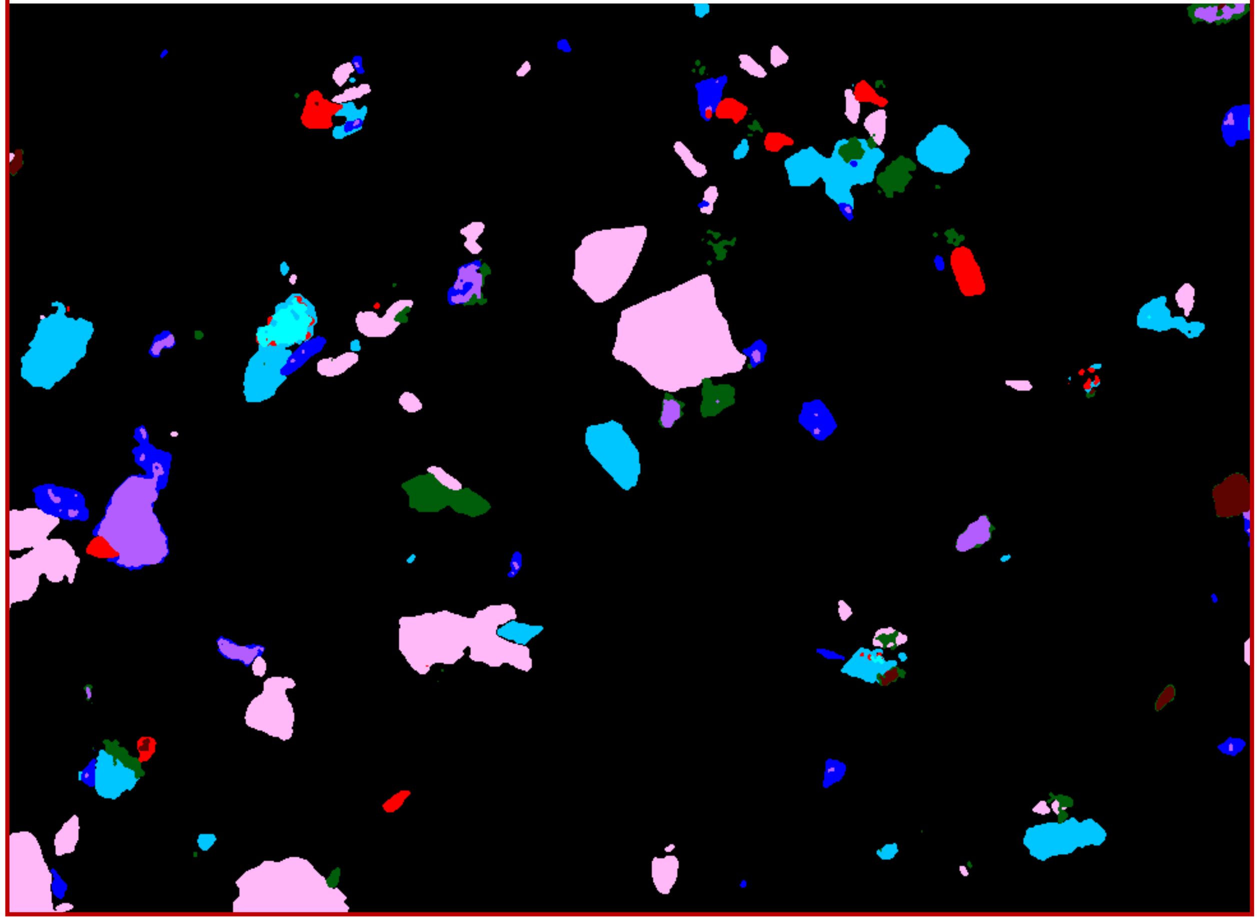

Output AH

Area % normalized to 100%

Quartz	39.3
Calcite	8.5
K-feldspar	14.0
Feldspar	21.6
Mica/clay	7.3
Olivine/OPX	1.1
Fe-Oxide	5.9
Mg-calcite	2.4

31.0%	"O"+"Si"
31.0%	"Si"
11.0%	"K"
11.0%	"Al"+"K"
9.84%	"Al"+"Ca"
7.23%	"Al"+"Na"
6.72%	"Ca"
5.74%	"Al"+"K"+"Mg"
5.74%	"K"+"Mg"
4.67%	"Fe"
1.86%	"Ca"+"Mg"
0.83%	"Fe"+"Mg"

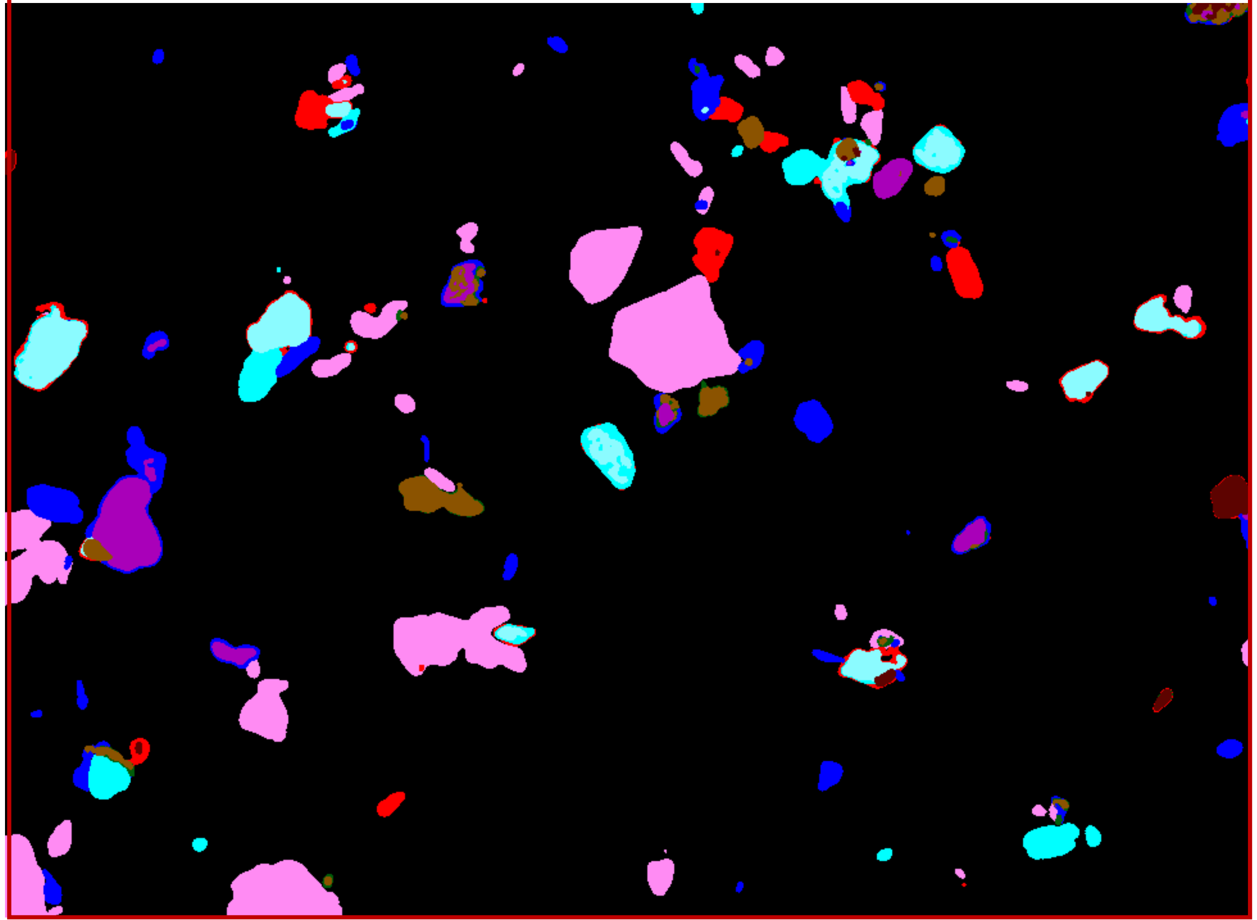

Map 2:

Multi-elemental EDS map

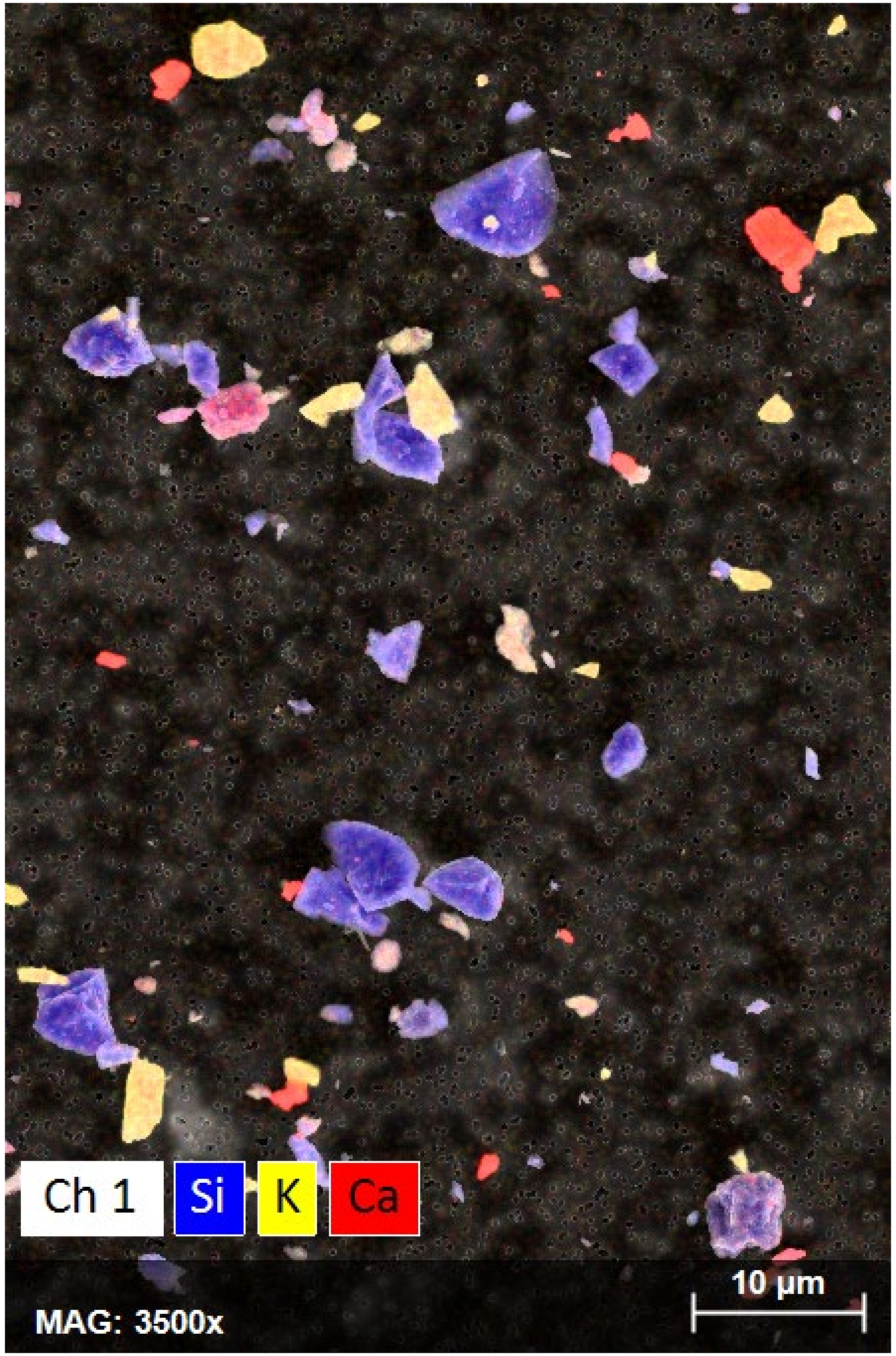

Map 2: Phase maps

Output JE

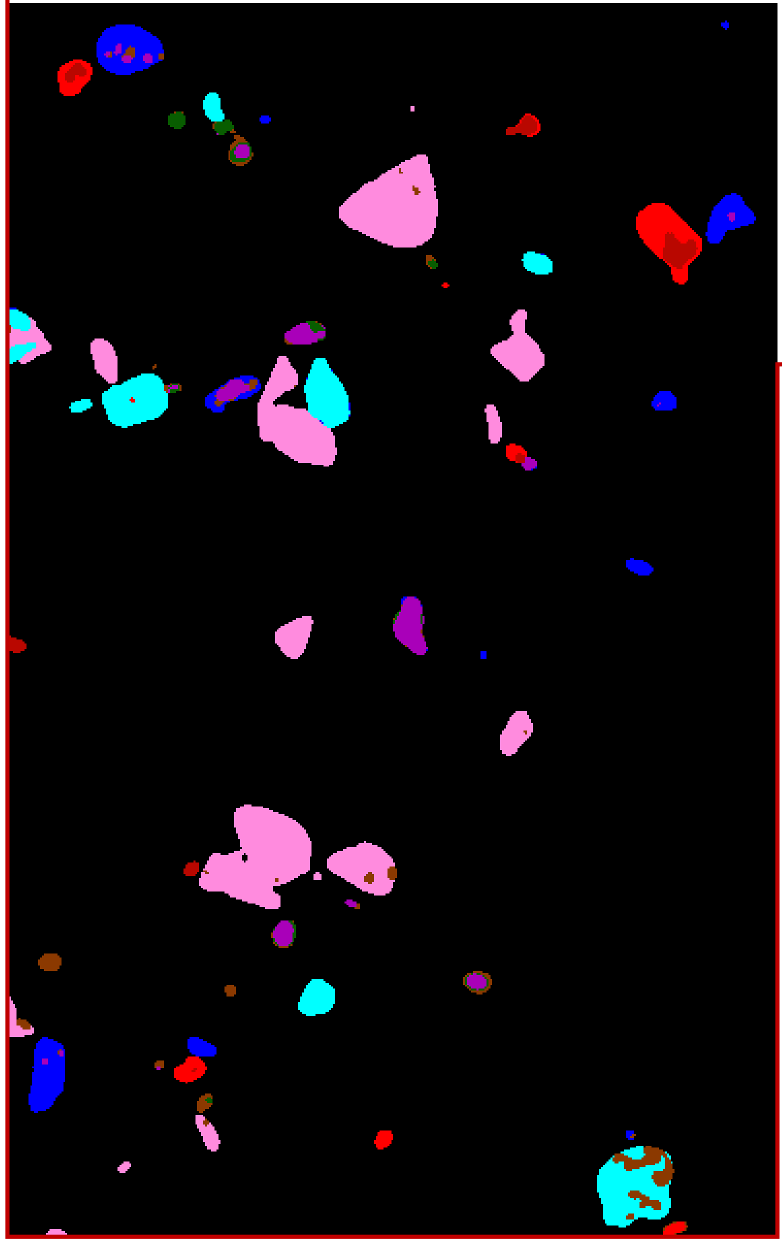

			Area % normalized to 100%
Crystalline silica (quartz)	33.9%	"Si"	42.8
K-feldspar	15.7%	"Na"	19.8
Plagioclase (all compositions)	14.4%	"K"	18.3
Calcite	5.37%	"Ca"	6.8
Clay/Mica	3.56%	"K"+"Mg"	4.5
Mg-calcite	2.87%	"Mg"	3.6
Pyroxene + Olivine	2.54%	"Ca"+"Mg"	3.2
Undetermined aluminosilicate	0.77%	"Al"+"Si"	1.0

Output AH

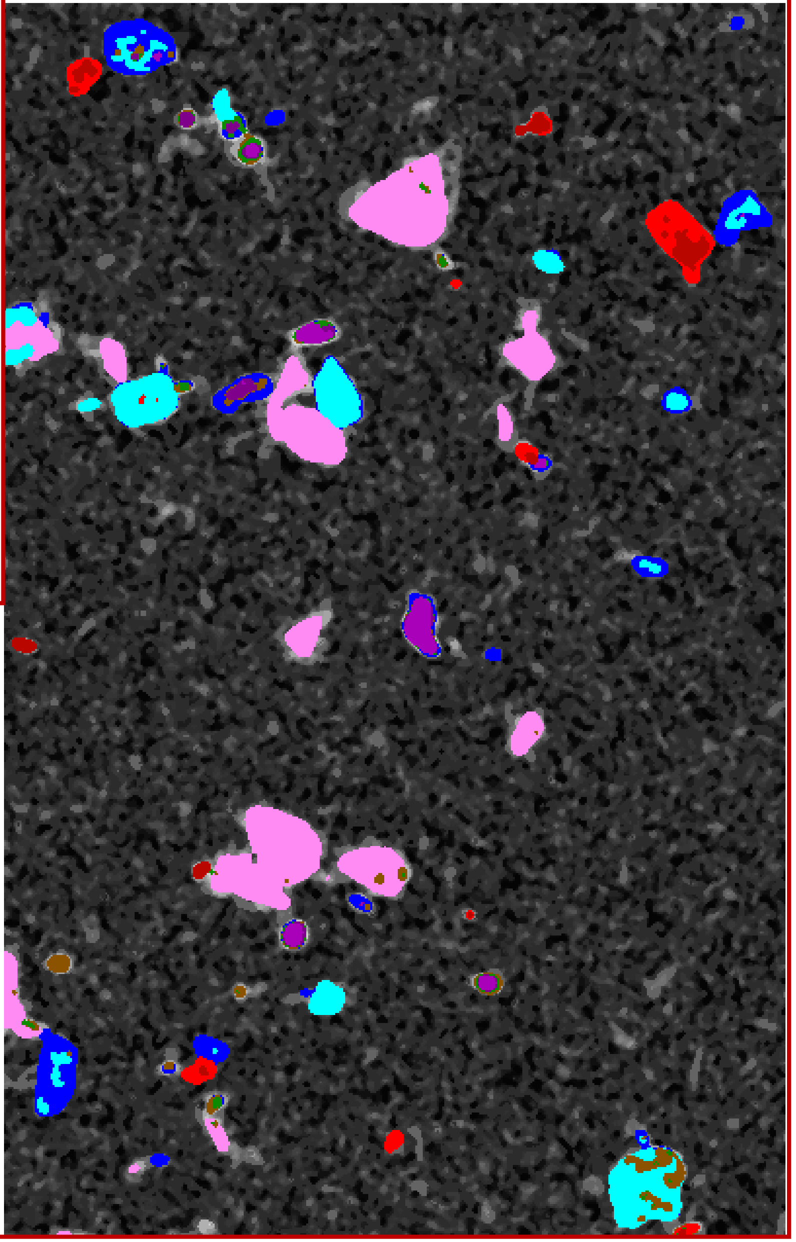

Crystalline silica (quartz)	36.5%	"Si"
Plagioclase (all compositions)	18.0%	"Al"+"Na"
K-feldspar	14.3%	"Al"+"K"
Calcite	5.77%	"Ca"
Fe oxide	4.24%	"Fe"
Mica	3.43%	"K"+"Mg"
Mg-calcite	3.36%	"Ca"+"Mg"
Pyroxene (OPX) or Olivine	1.64%	"Fe"+"Mg"
Mica	1.51%	"Fe"+"K"+"Mg"

Area % normalized to 100%

Quartz	44.9
Calcite	7.3
K-feldspar	12.5
Feldspar	18.4
Mica/clay	6.1
Olivine/OPX	1.9
Fe-Oxide	5.8
Mg-calcite	3.1

Map 3:

Multi-elemental EDS map

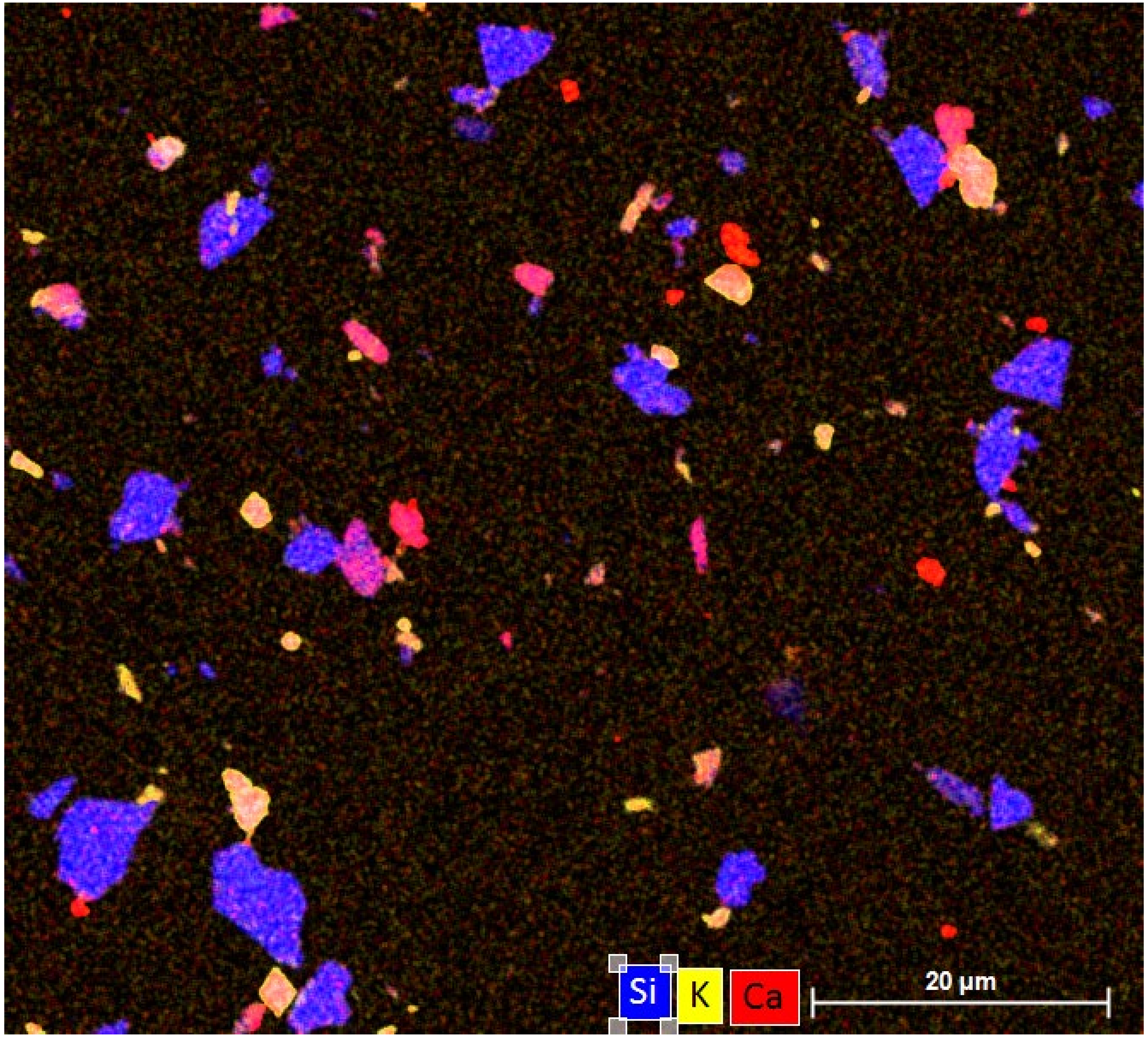

Map 3: Phase maps

Output JE

Area % normalized to 100%

Crystalline silica (quartz)	39.8%	"Si"	54.0
Plagioclase (all compositions)	8.60%	"Na"	11.7
K-feldspar	7.09%	"K"	9.6
Plagioclase	6.79%	"Al"+"Ca"	9.2
Pyroxene + Olivine + Fe oxide	6.06%	"Mg"	8.2
Calcite	3.56%	"Ca"	4.8
Clay/Mica	1.82%	"K"+"Mg"	2.5

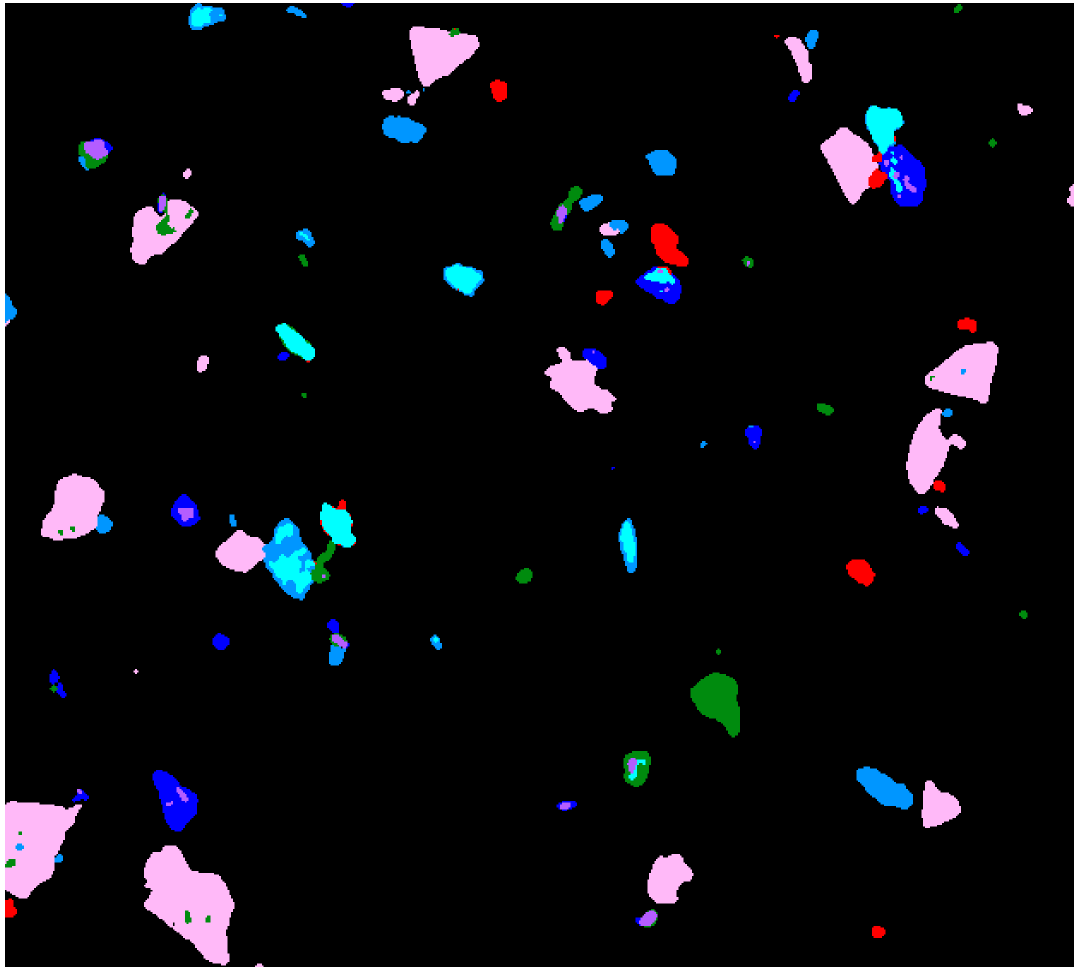

Output AH

Crystalline silica (quartz)	45.3%	"Si"
Clay/Mica	10.5%	"Al"+"K"+"Mg"
	10.5%	"Al"+"Mg"
	10.5%	"K"+"Mg"
Plagioclase (all compositions)	9.92%	"Na"
Calcite	6.69%	"Ca"
Olivine/OPX	3.69%	"Mg"
K-feldspar	2.46%	"K"

Area % normalized to 100%

Label	Area %
Quartz	45.4
Calcite	6.7
K-Feldspar	2.5
Plagioclase	9.9
Mica	10.5
Olivine/OPX	3.7

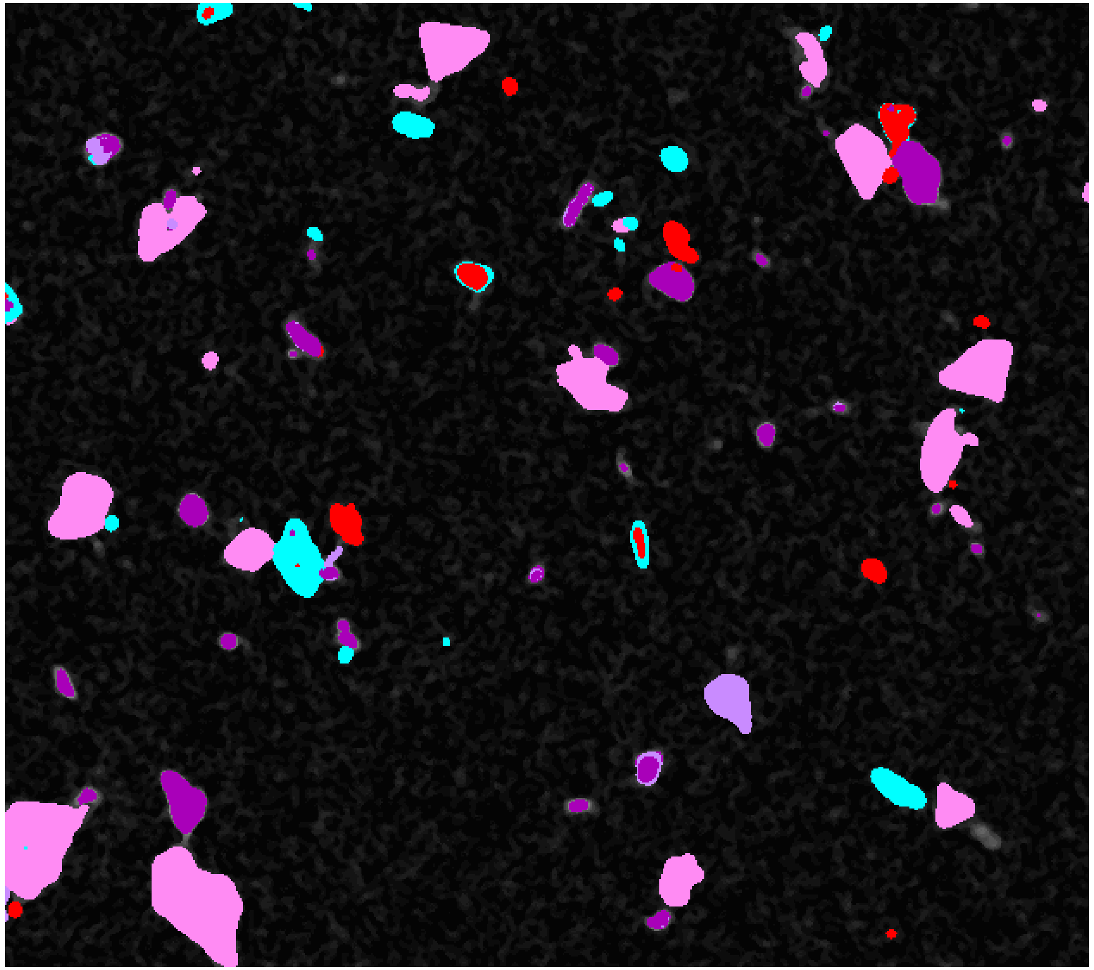

Summary ATD: Mean of maps 1 to 3

Output JE

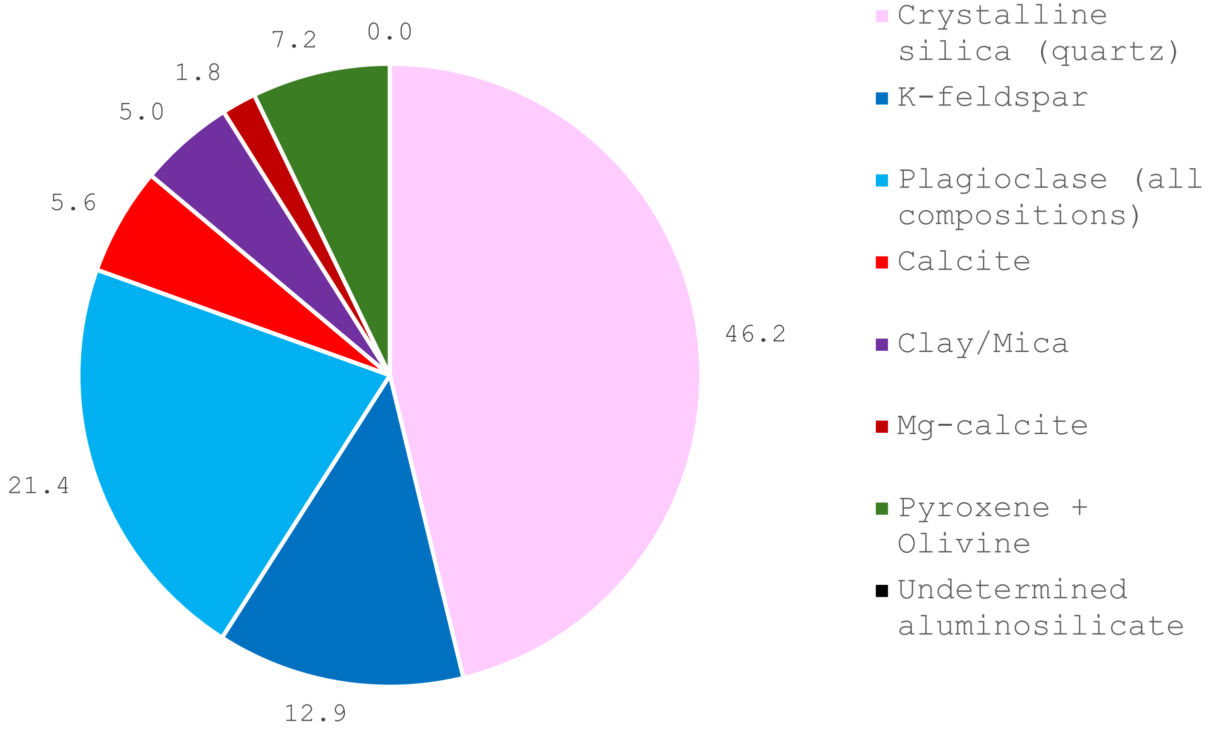

Output AH

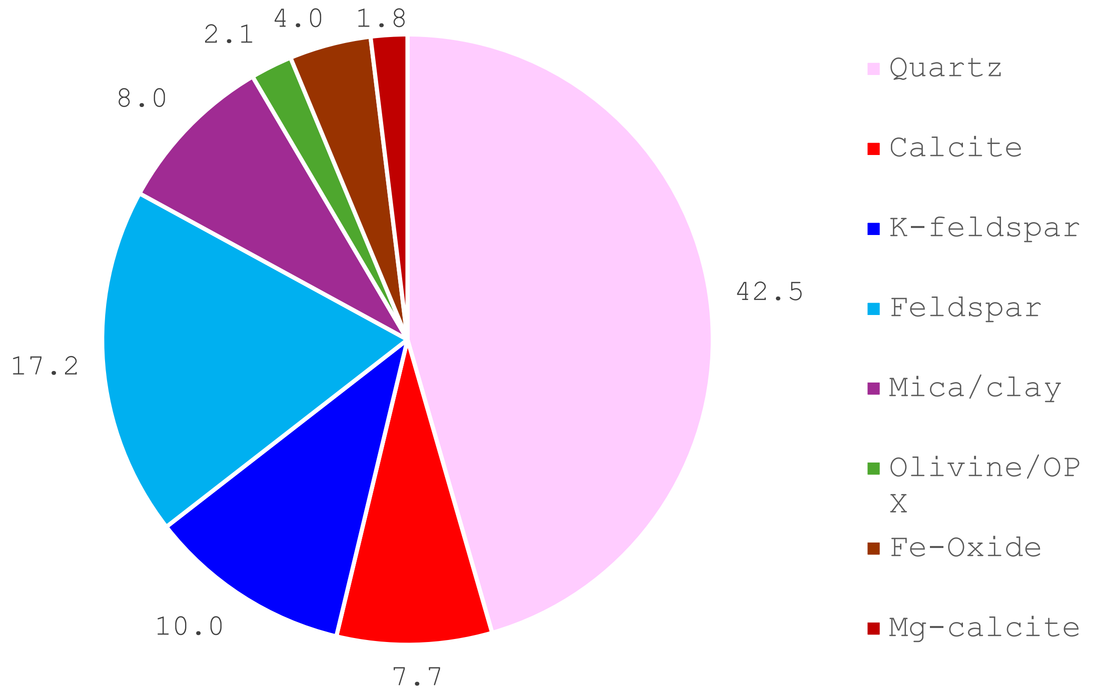

Gobi Desert Dust (GDD) maps

Map 1:

Multi-elemental EDS map

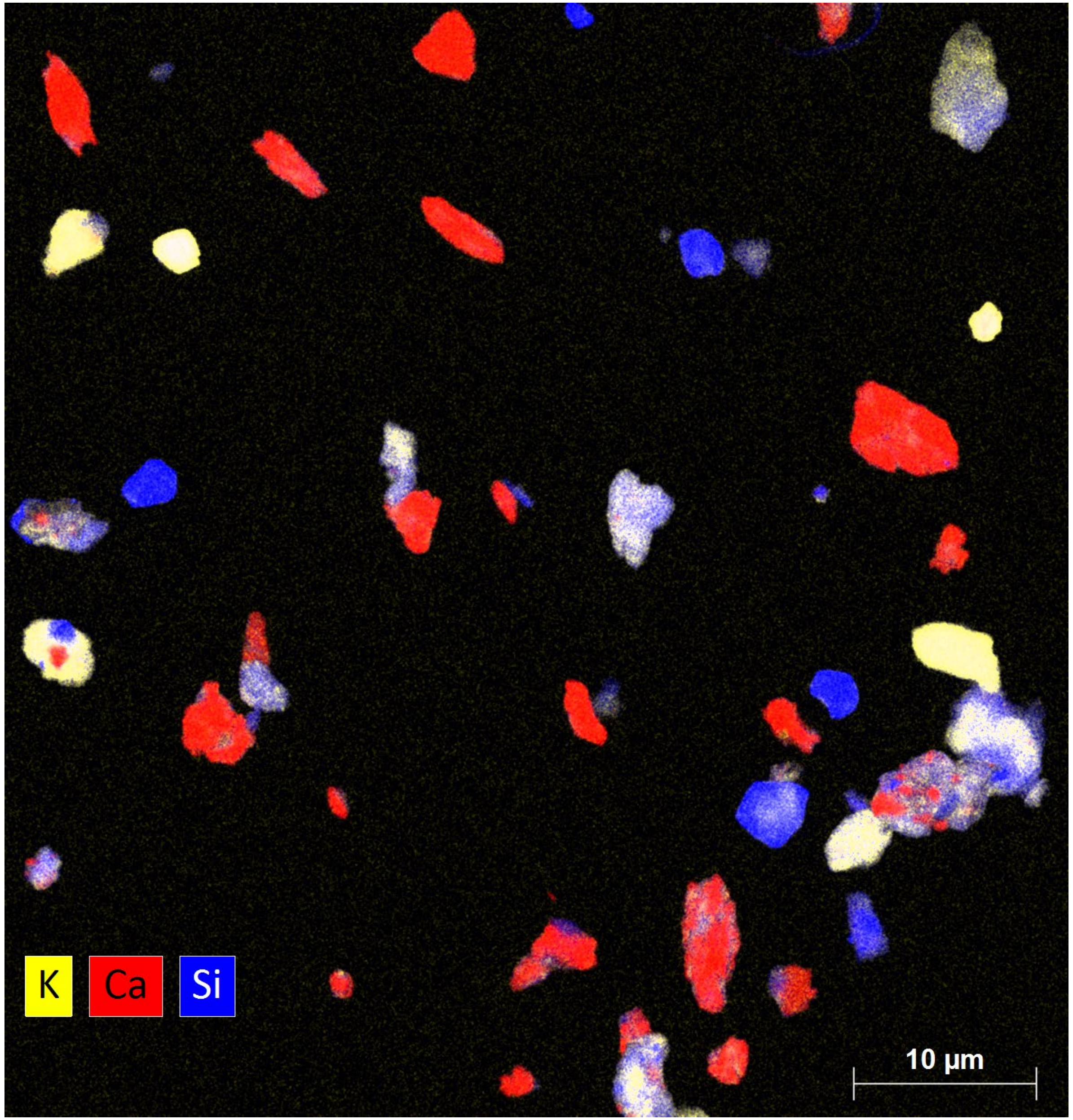

Map 1: Phase maps

Output JE

			Area % normalized to 100%
Calcite	37.5%	"Ca"	39.5
K-feldspar	29.2%	"K"	30.8
Mica/Clay	11.5%	"Al"+"Si"	12.1
Quartz	6.32%	"Si"	6.6
Olivine + Px + Fe-oxide	4.97%	"Mg"	5.2
Mg-calcite	2.88%	"Ca"+"Mg"	3.0
Plagio	2.55%	"Na"	2.7

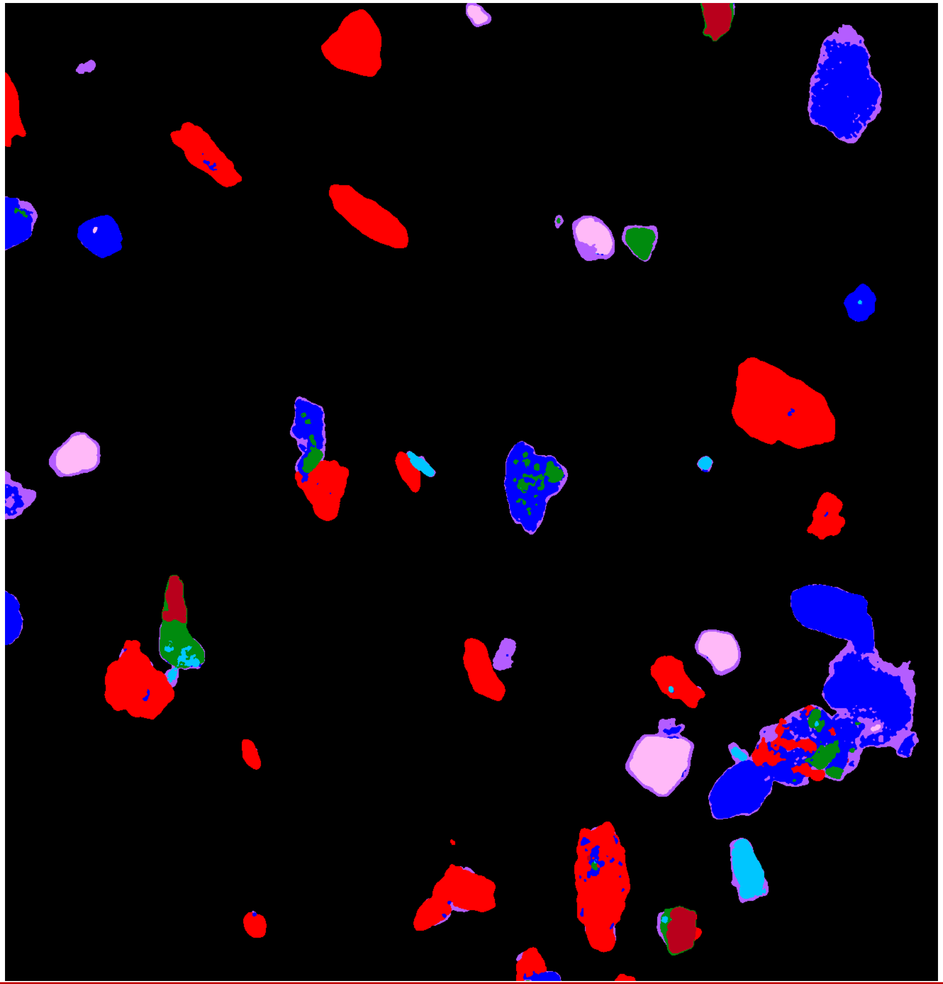

Output AH

Calcite	38.9%	"Ca"
K-feldspar	20.5%	"Al"+"K"
Mica/illite?	12.2%	"Mg"
Quartz	8.18%	"Si"
Feldspar	3.47%	"Na"
Fe-oxide	3.36%	"Fe"
Mg-calcite	2.21%	"Ca"+"Mg"

Area % normalized to 100%	
K-feldspar	23.1
Calcite	43.8
Quartz	9.2
Clay	13.8
Plagioclase	3.9
Fe-Ti oxide	3.8
Olivine/OPX	0.0
Mg-calcite	2.5
S-bearing	0.0

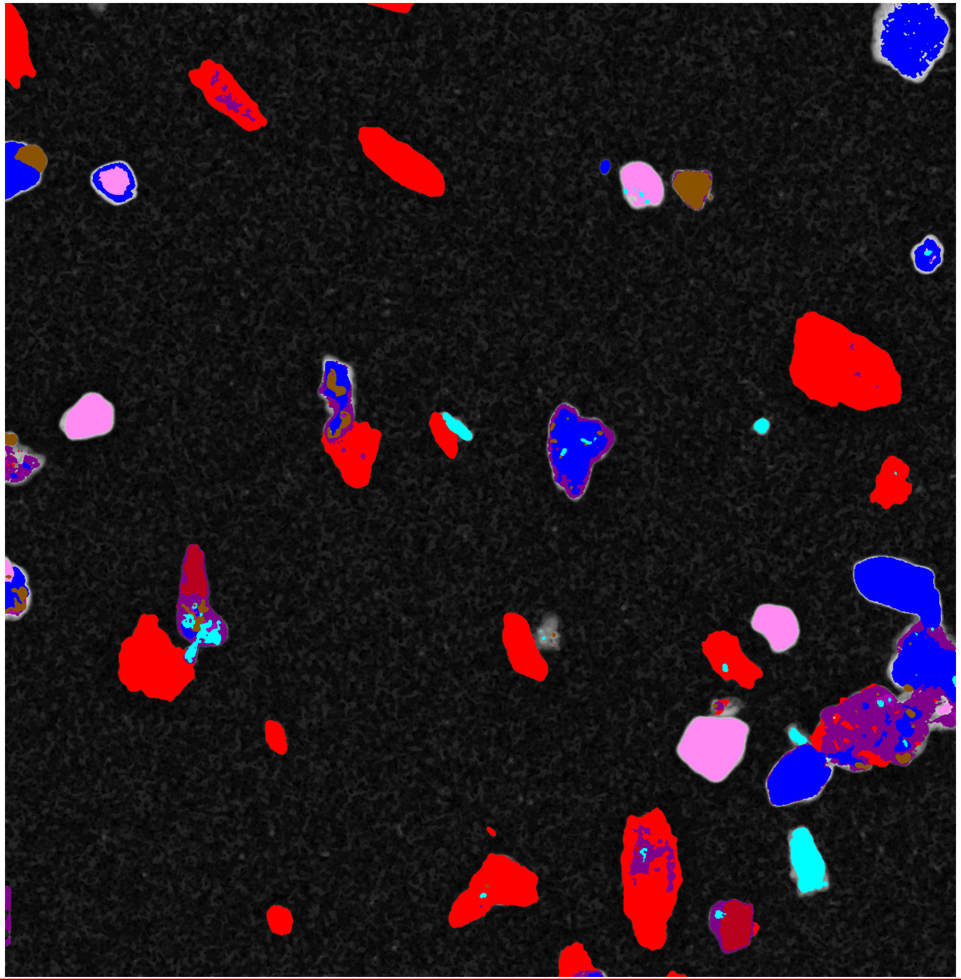

Map 2:

Multi-elemental EDS map

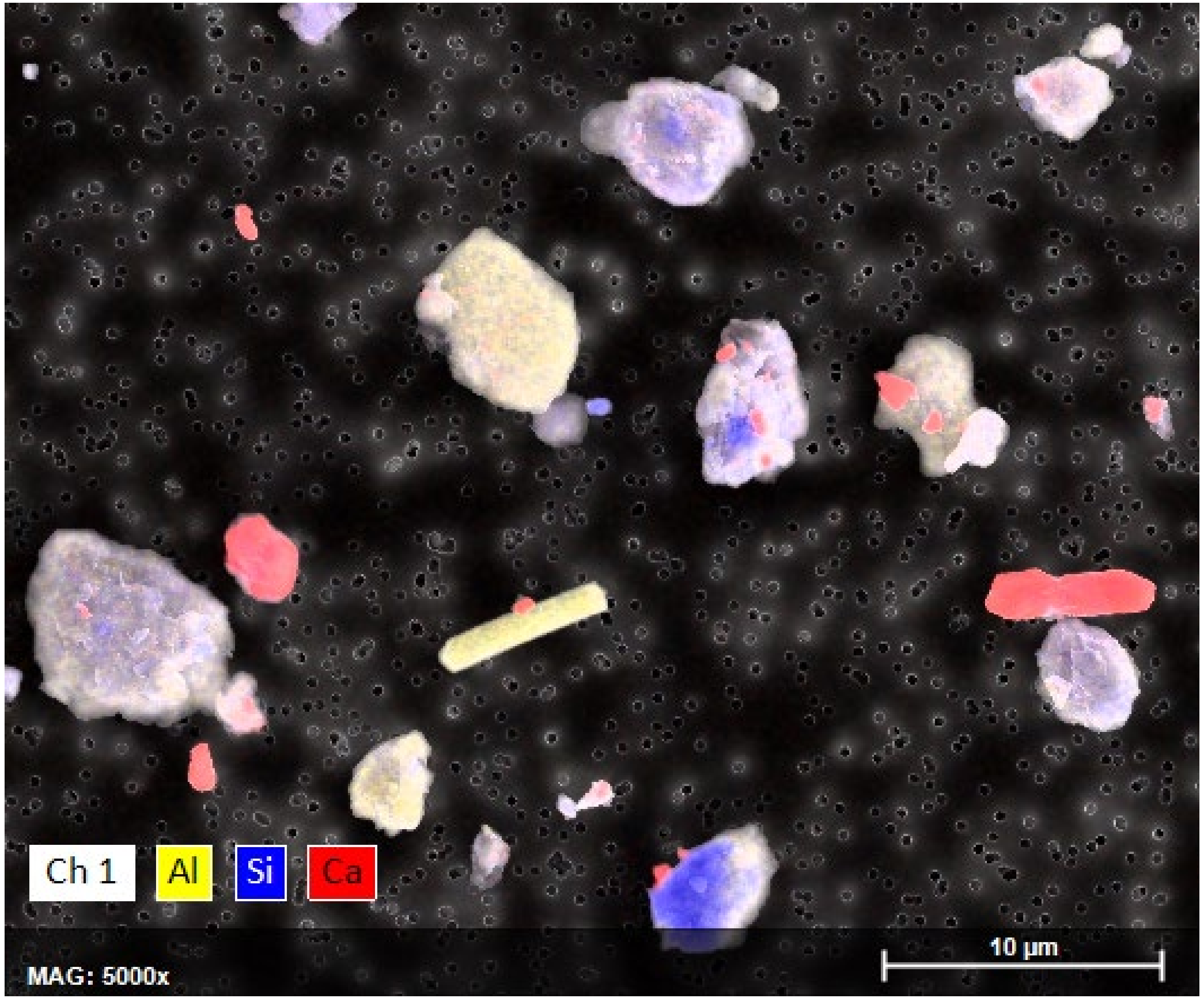

Map 2: Phase maps

Output JE

Phase	%	Element(s)	Area % normalized to 100%
K-feldspar	49.0%	"K"	55.5
Calcite	12.8%	"Ca"	14.6
Plagio	11.6%	"Na"	13.1
Mica/Clay	9.39%	"Al"	10.6
Quartz	4.13%	"Si"	4.7
Olivine + Px + Fe-oxide	0.73%	"Mg"	0.8
Mg-calcite	0.61%	"Ca"+"Mg"	0.7

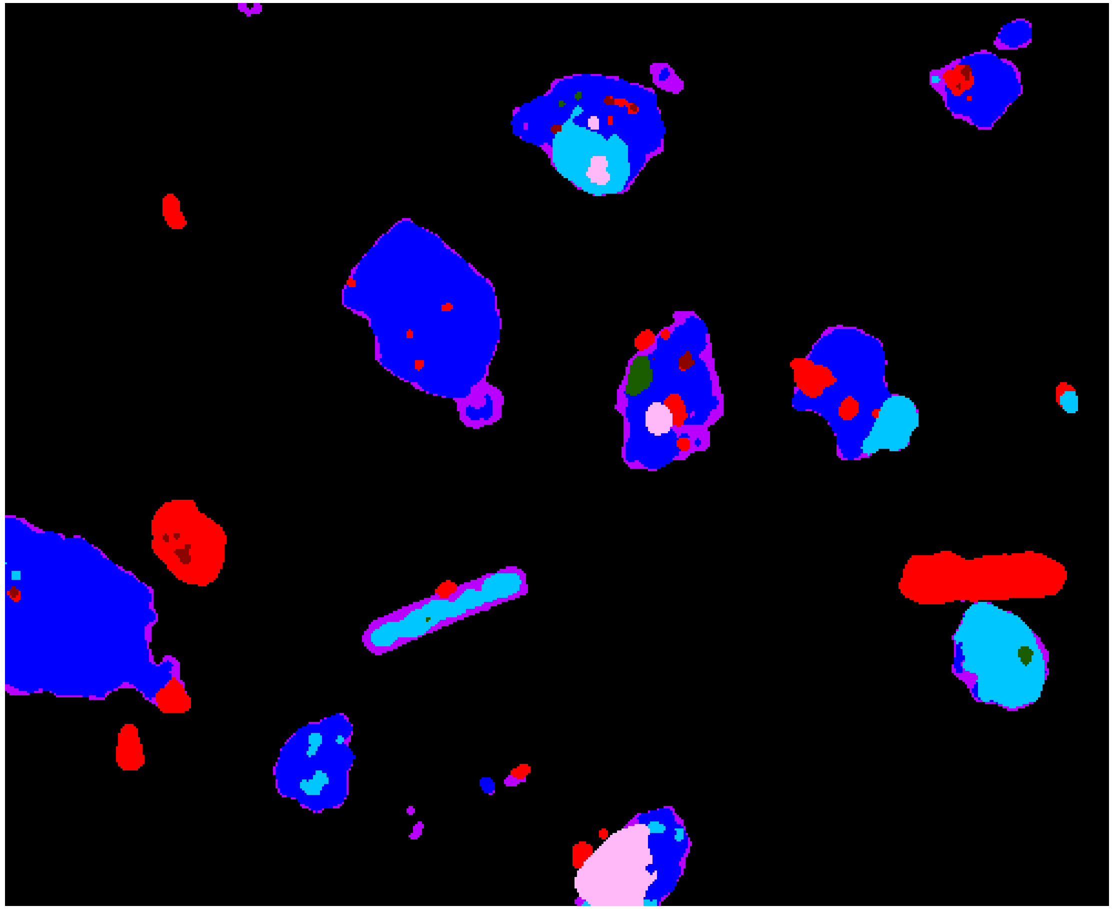

Output AH

Phase	%	Element(s)
K-feldspar	32.4%	"Al"+"K"+"Si"
Calcite	12.6%	"Ca"
K-feldspar	8.48%	"Al"+"K"+"O"
Feldspar	5.41%	"Na"
Quartz	5.31%	"O"+"Si"
Clay?	4.84%	"Al"+"Mg"+"Na"+"O"+"Si
Fe-oxide	4.75%	"Fe"+"Ti"
Feldspar	2.00%	"Al"+"Na"+"Si"
Olivine/OPX	1.42%	"Fe"+"Mg"+"Si"

Phase	Area % normalized to 100%
K-feldspar	52.9
Calcite	16.3
Quartz	6.9
Clay	6.3
Plagioclase	9.6
Fe-Ti oxide	6.2
Olivine/OPX	1.8
Mg-calcite	0.0
S-bearing	0.0

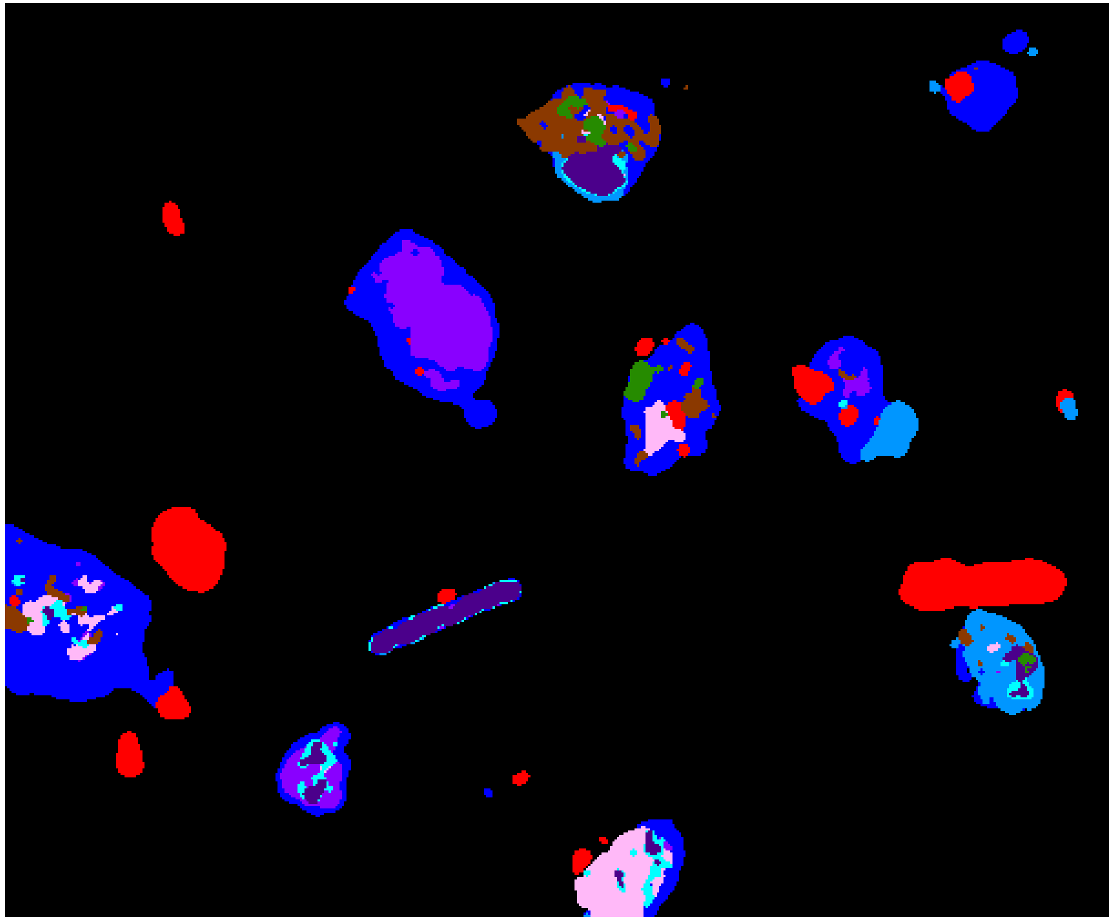

Map 3:

Multi-elemental EDS map

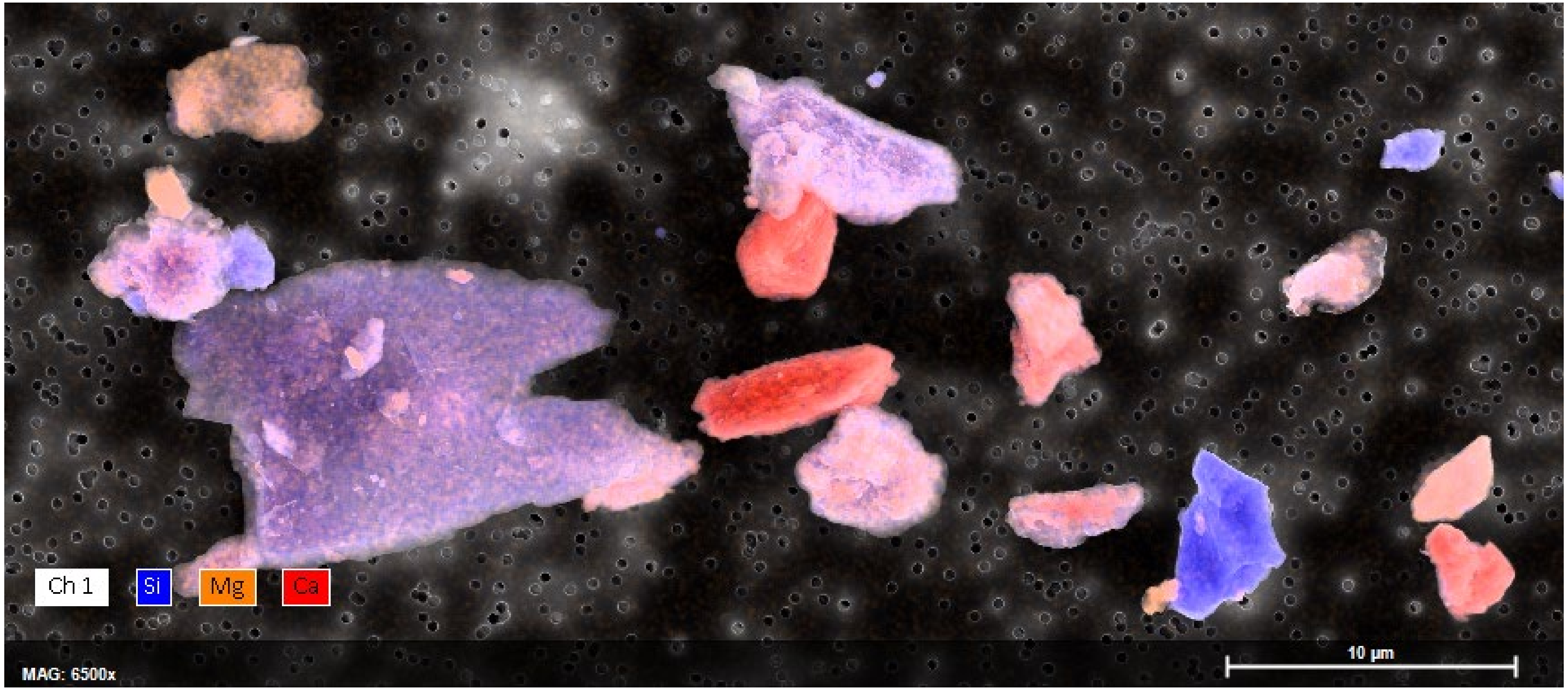

Map 3: Phase maps

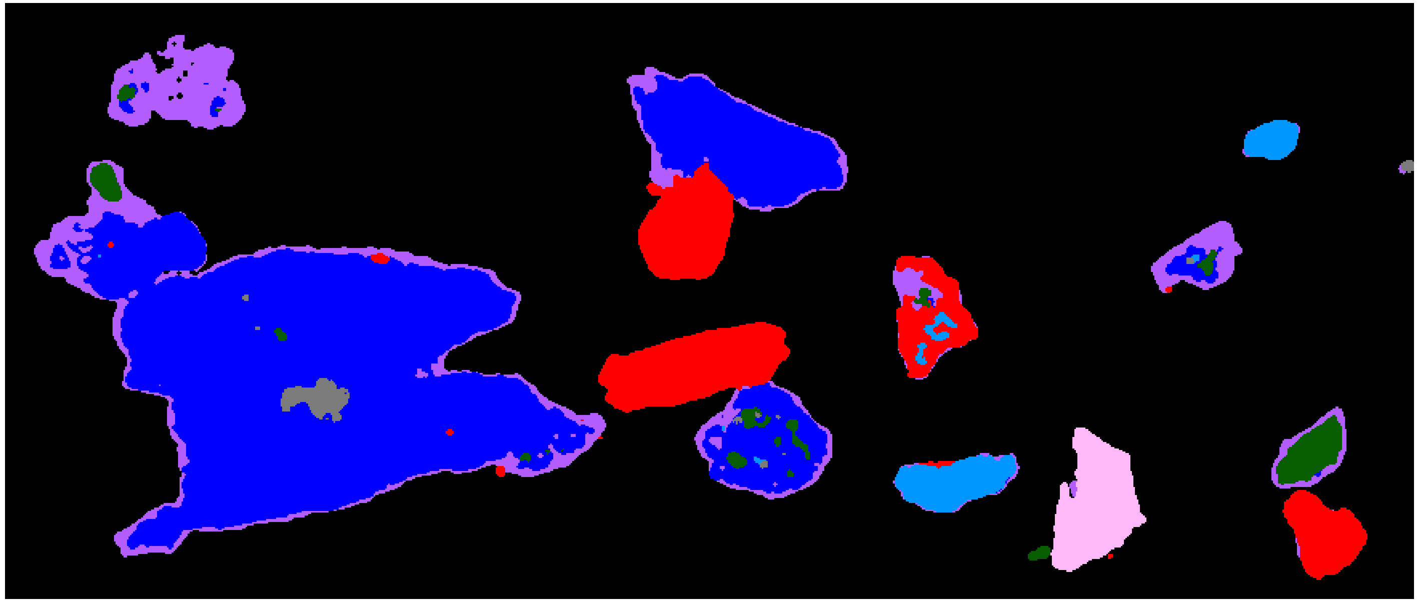

Output JE

			Area % normalized to 100%
K-feldspar	56.6%	"K"	58.8
Mica/Clay	14.5%	"Al"	15.2
Calcite	14.0%	"Ca"	14.5
Quartz	4.03%	"Si"	4.2
Plagio	3.35%	"Na"	3.5
Olivine + Px + Fe-oxide	2.71%	"Mg"	2.8
Anthropogenic pollutant	1.01%	"Ti"	1.1

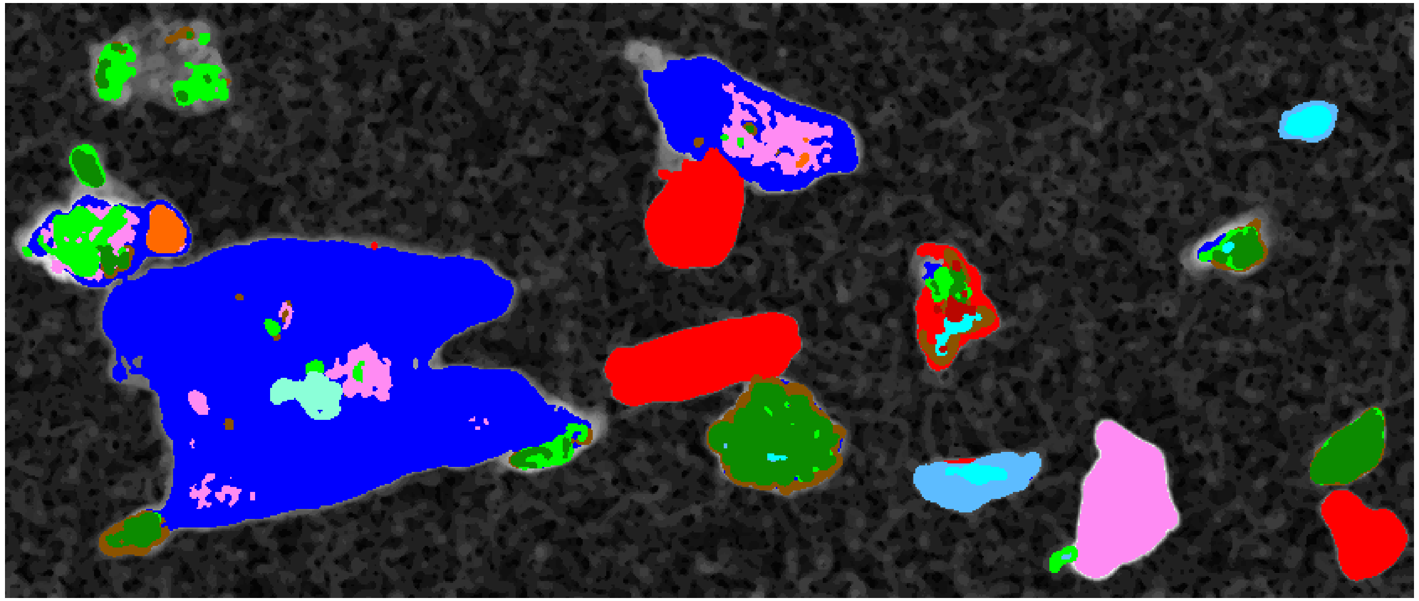

Output AH

K-feldspar	44.5%	"Al"+"K"
Calcite	12.7%	"Ca"
Quartz	9.01%	"Si"
Olivine/OPX	8.00%	"Fe"+"Mg"
Mica/illite	4.57%	"Mg"
Fe-oxide	2.95%	"Fe"
Feldspar	2.32%	"Na"
Feldspar	1.26%	"Ca"+"Na"+"Si"
Ti-sulfate?	1.01%	"S"+"Ti"
?	0.74%	"K"+"Si"
Mg-calcite	0.33%	"Ca"+"Mg"

	Area % normalized to 100%
K-feldspar	51.8
Calcite	14.6
Quartz	10.3
Clay	5.2
Plagioclase	4.1
Fe-Ti oxide	3.4
Olivine/OPX	9.1
Mg-calcite	0.4
S-bearing	1.2

Map 4:

Multi-elemental EDS map

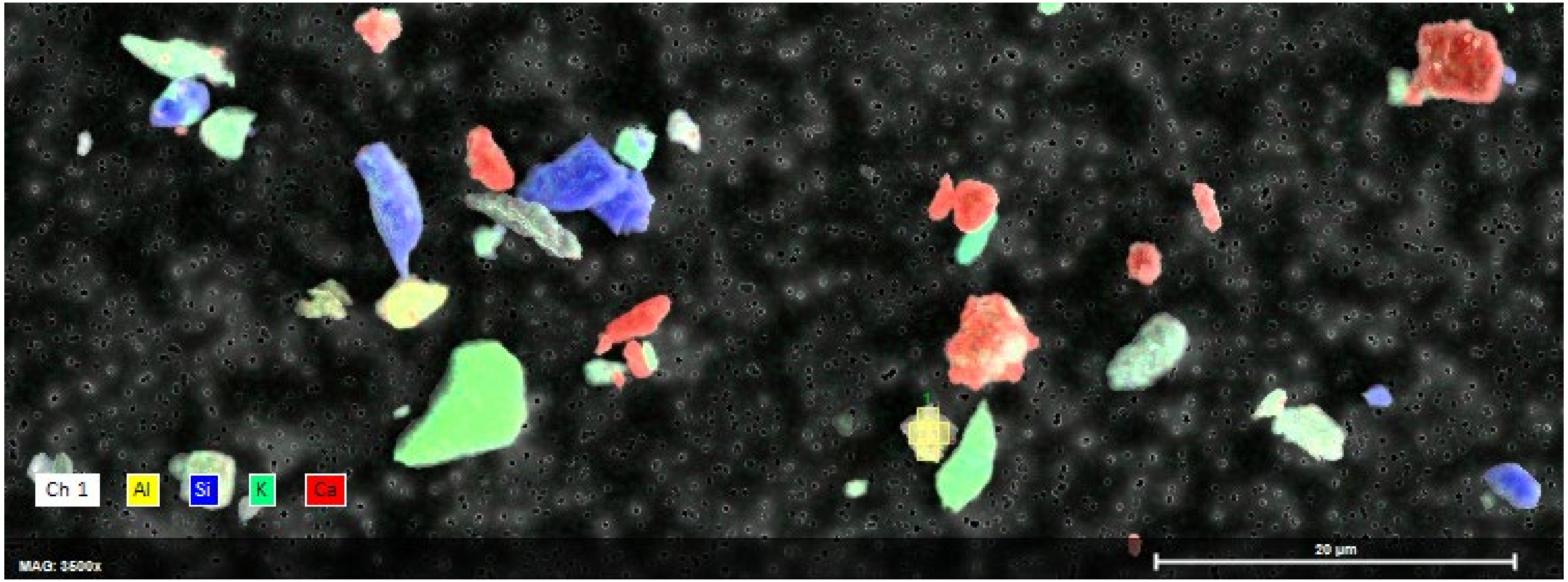

Map 4: Phase maps

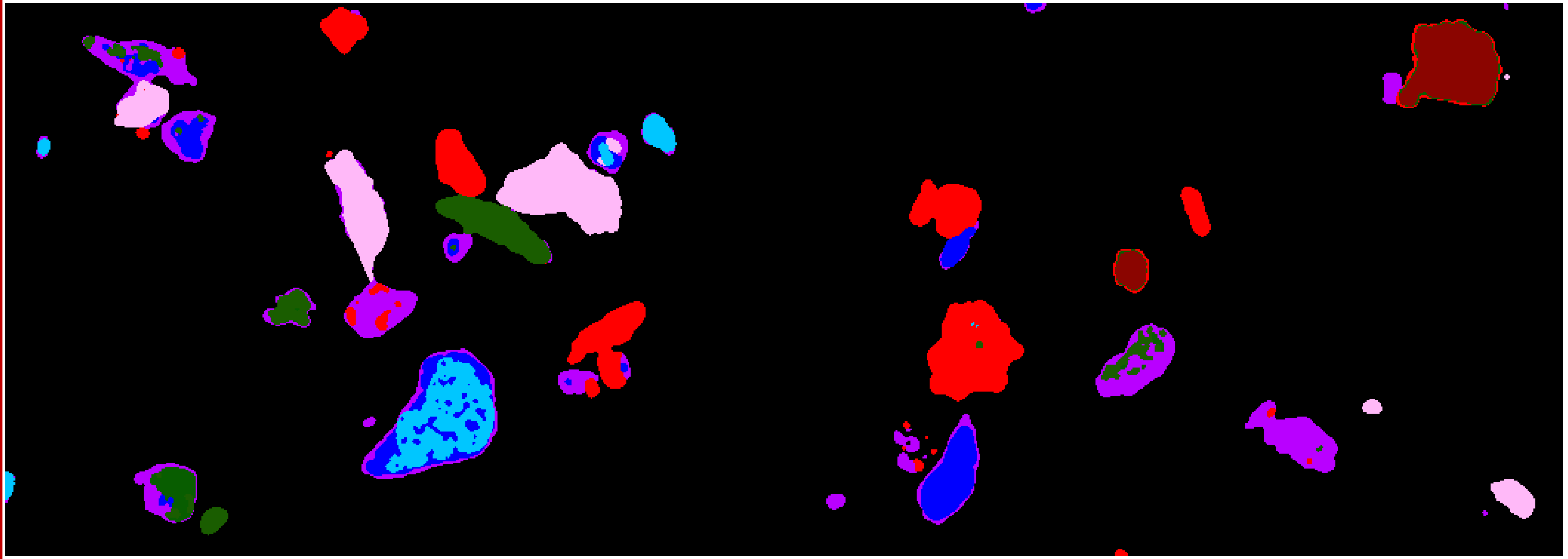

Output JE

Phase	Area %	Element(s)	Area % normalized to 100%
Calcite	22.5%	"Ca"	22.9
Mica/Clay	20.8%	"Al"	21.2
Quartz	16.0%	"Si"	16.3
K-feldspar	11.0%	"K"	11.3
Plagio	9.04%	"Na"	9.2
Fe-oxide	8.15%	"Fe"	8.3
Mg calcite	8.03%	"Ca"+"Mg"	8.2
Olivine + Px	2.58%	"Mg"	2.6

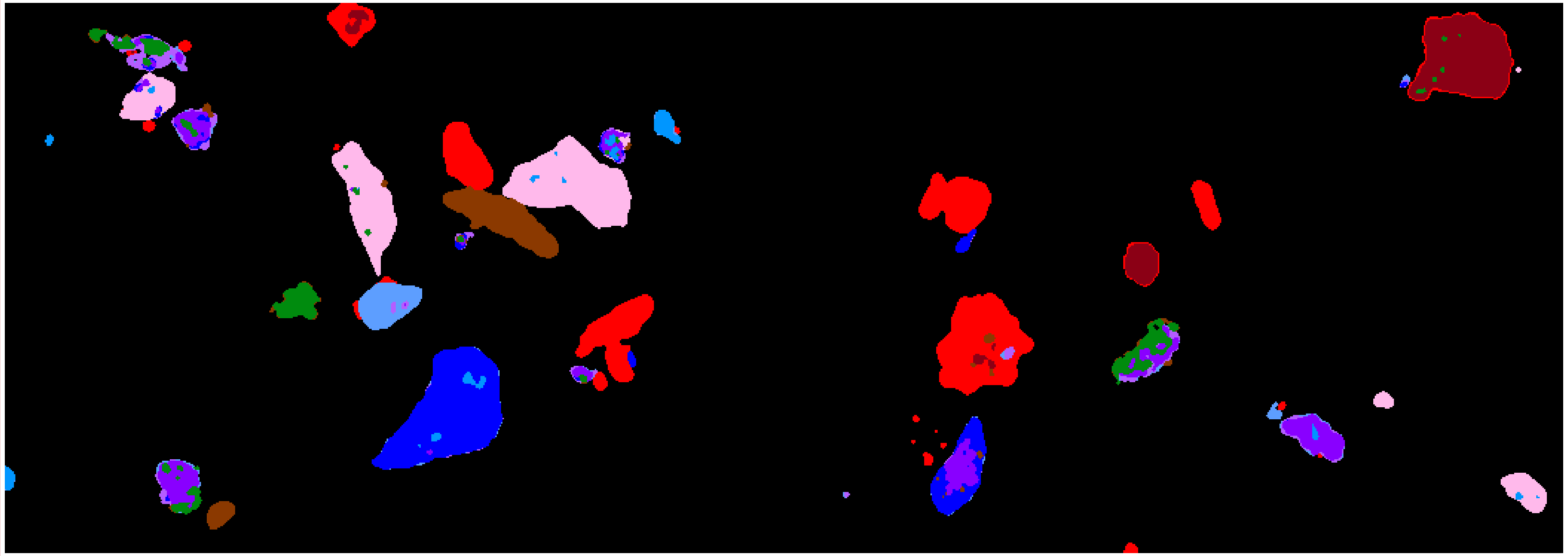

Output AH

Phase	Area %	Element(s)
Calcite	22.6%	"Ca"
Quartz	17.2%	"Si"
K-feldspar	14.3%	"Al"+"K"+"Si"
K-feldspar	14.3%	"Al"+"K"
Mg-calcite	9.73%	"Ca"+"Mg"
Clay?	7.55%	"Al"+"Mg"+"Si"
Fe-oxide	6.10%	"Fe"
Olivine/OPX	5.18%	"Fe"+"Mg"+"Si"
Feldspar	3.42%	"Al"
Clay?	2.70%	"Al"+"K"+"Mg"+"Si"
Feldspar	1.84%	"Na"+"Si"

Area % normalized to 100%	
K-feldspar	15.8
Calcite	25.0
Quartz	19.0
Clay	11.3
Plagioclase	5.8
Fe-Ti oxide	6.7
Olivine/OPX	5.7
Mg-calcite	10.7
S-bearing	0.0

Summary GDD: Mean of maps 1 to 4

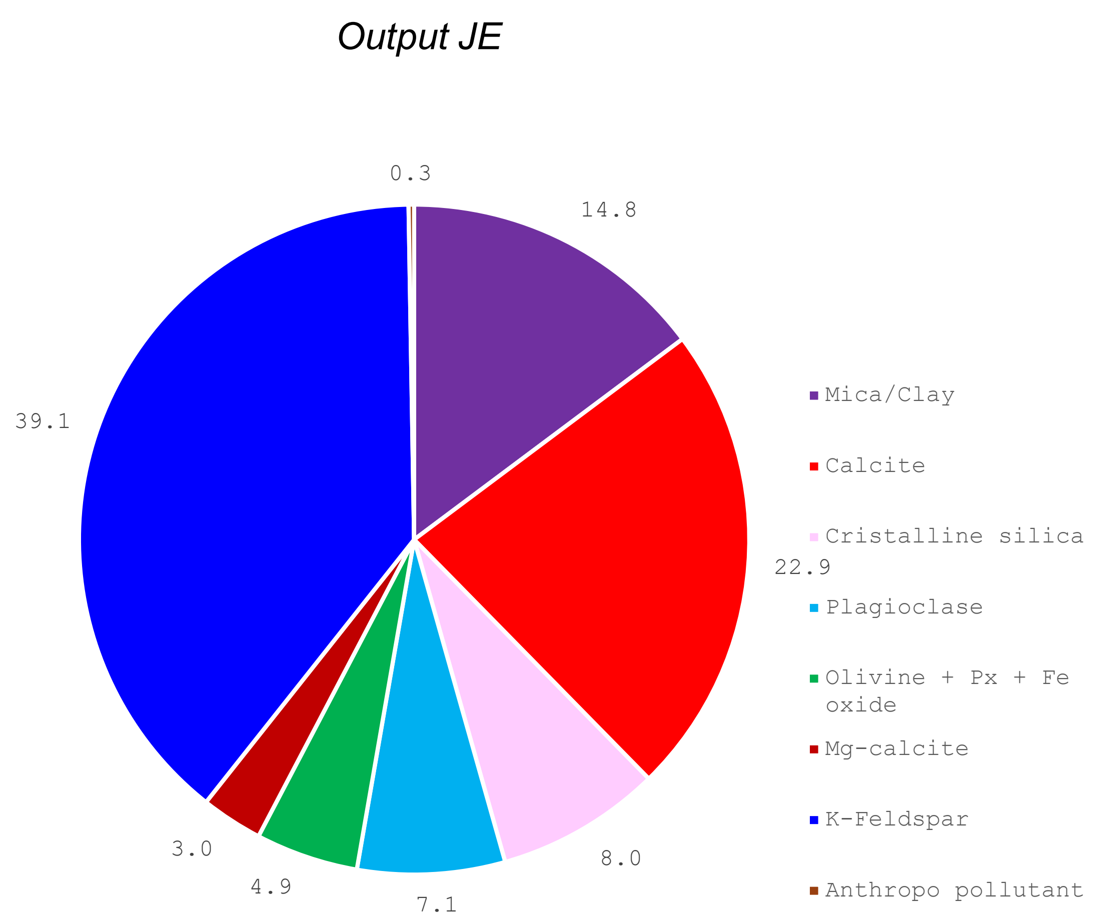

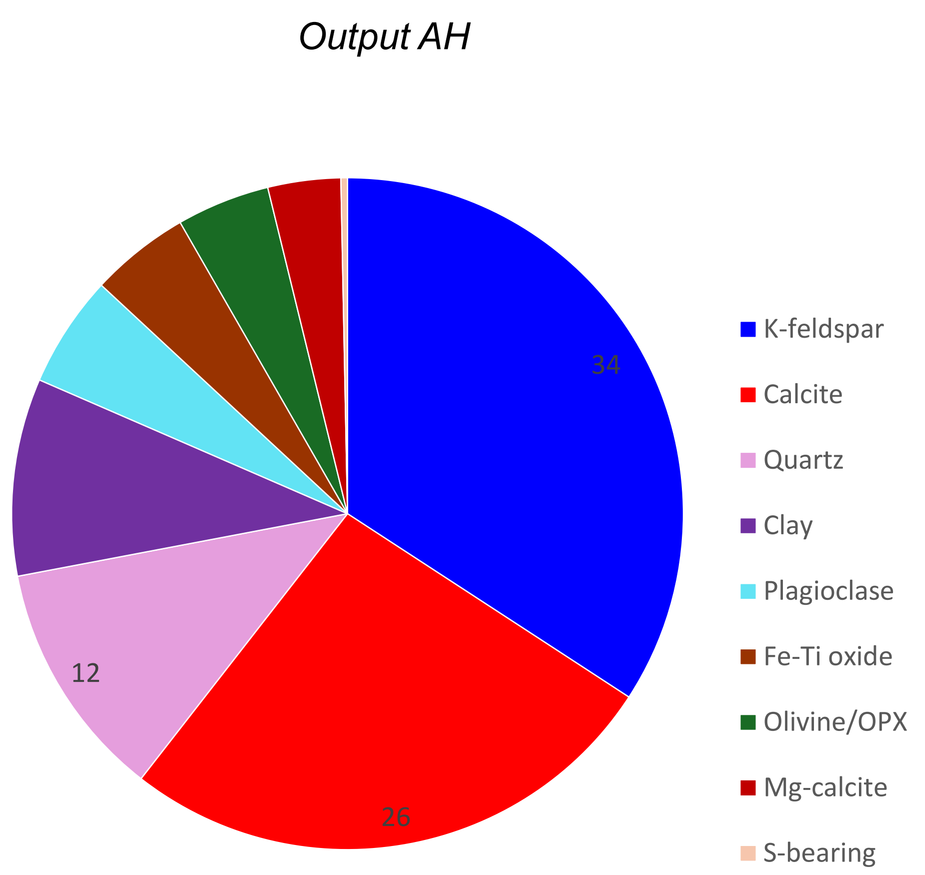

St Vincent volcanic ash (SVA) maps

Map 1:

Multi-elemental EDS map

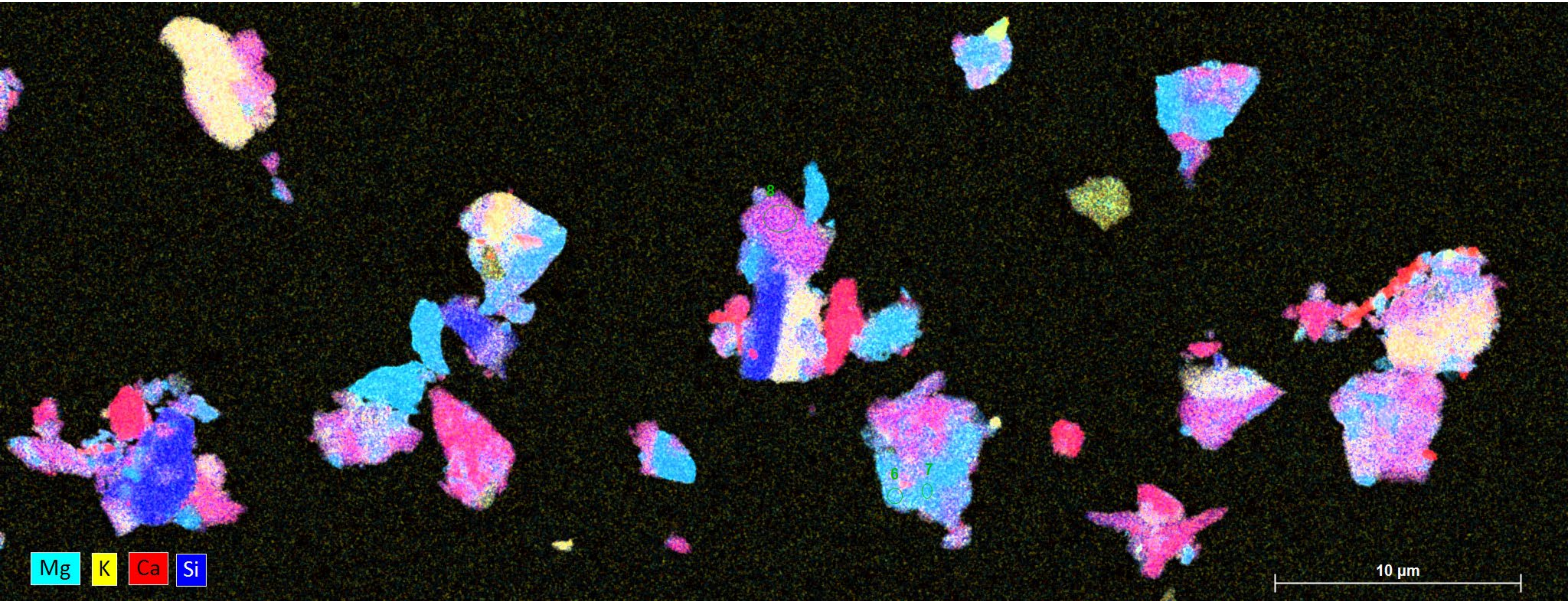

Map 1: Phase maps

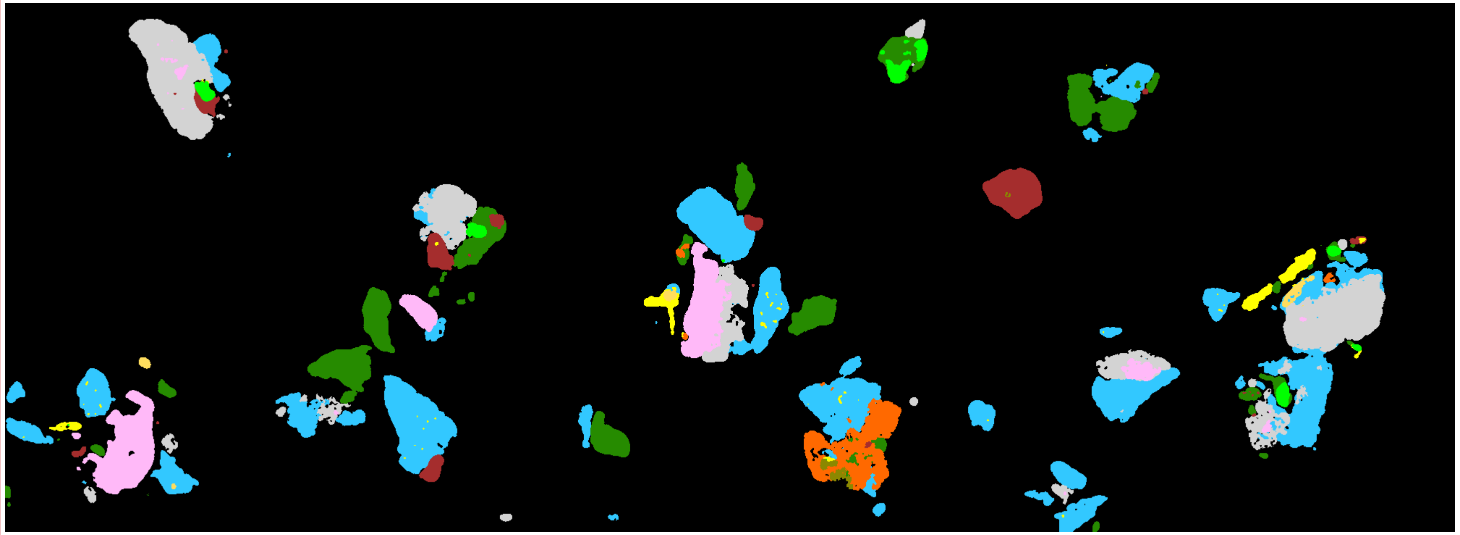

Output JE

Phase	Area %	Elements	Area % normalized to 100%
Plagioclase (albite to anorthite)	25.4%	"Al"+"Ca"+"Na"	37.7
Glass (all compositions)	14.7%	"K"	21.9
Pyroxene (OPX) or Olivine	10.2%	"Mg"	15.1
Crystalline silica	7.05%	"Si"	10.5
Chloride of some type + phosphate	3.28%	"Cl"	4.9
Fe-Ti oxide	3.28%	"Fe"+"Ti"	4.9
Anhydrite	1.43%	"Ca"+"S"	2.1
CPX	1.28%	"Ca"+"Mg"	1.9
Natroalunite	0.42%	"Al"+"S"	0.6
Pyrite and/or Fe-chloride	0.28%	"Cl"+"S"	0.4

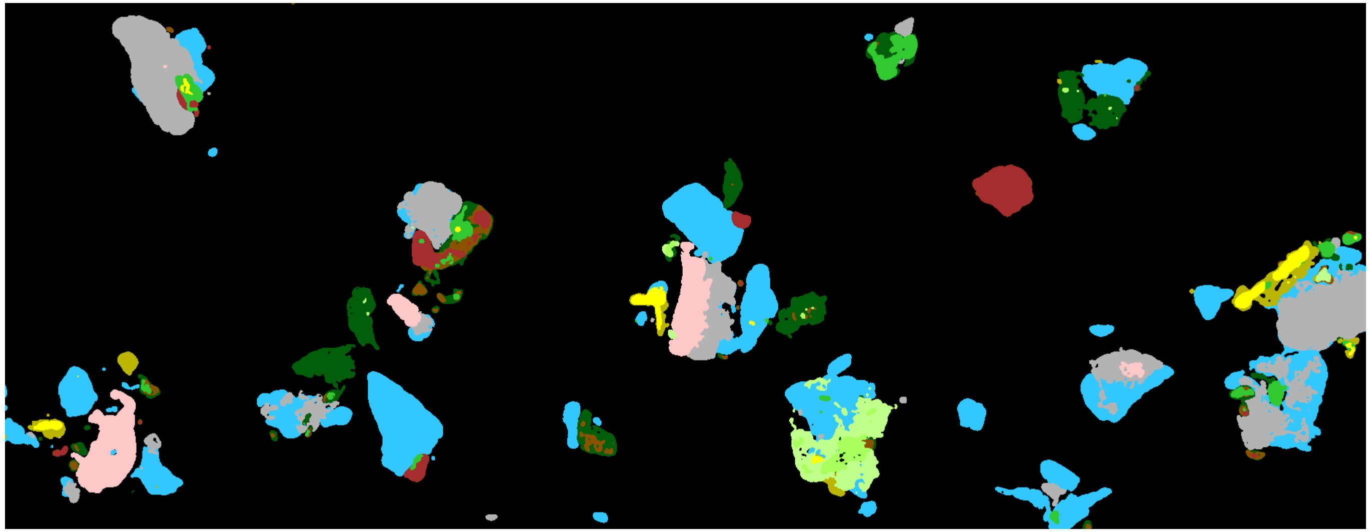

Output AH

Phase	Area %	Elements
Plagioclase (albite to anorthite)	28.7%	"Al"+"Ca"+"Na"
Glass (all compositions)	19.2%	"K"+"Si"
Pyroxene (OPX) or Olivine	8.62%	"Fe"+"Mg"
Crystalline silica	6.17%	"Si"
Chloride of some type	3.62%	"Cl"
Fe-Ti oxide	3.51%	"Fe"+"Ti"
CPX	2.93%	"Ca"+"Mg"+"Fe"
Pyrite and/or sulfate	2.10%	"S"
Fe-oxide	1.63%	"Fe"
Anhydrite	1.61%	"Ca"+"S"
Mg-Chloride?	1.04%	"Cl"+"Mg"

Map 2:

Multi-elemental EDS map

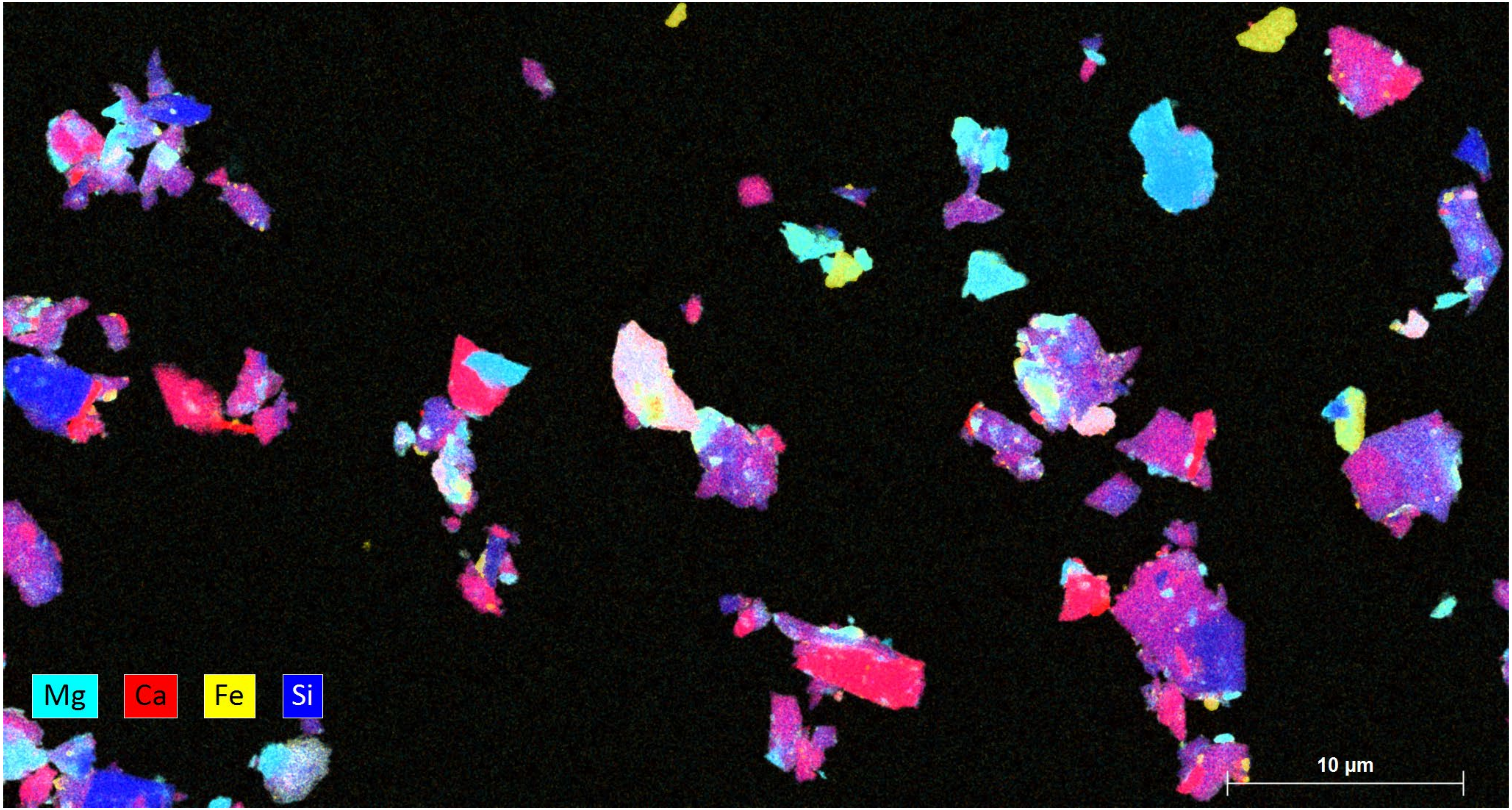

Map 2: Phase maps

Output JE

			Area % normalized to 100%
Plagioclase (albite to anorthite)	22.7%	"Al"+"Ca"+"Na"	34.0
Glass (all compositions)	19.2%	"K"	28.7
Pyroxene (OPX) + Olivine	8.12%	"Mg"	12.1
CPX	3.99%	"Ca"+"Mg"	6.0
Chloride of some type	3.93%	"Cl2"	5.9
Fe-Ti oxide	3.12%	"Fe"+"Ti"	4.7
Crystalline silica	2.23%	"Si"	3.3
Anhydrite	1.45%	"Ca"+"S"	2.2
Mica	1.06%	"K"+"Mg"	1.6
Pyrite and/or Fe-chloride	0.94%	"Cl2"+"S2"	1.4
Natroalunite	0.16%	"Al"+"S2"	0.2

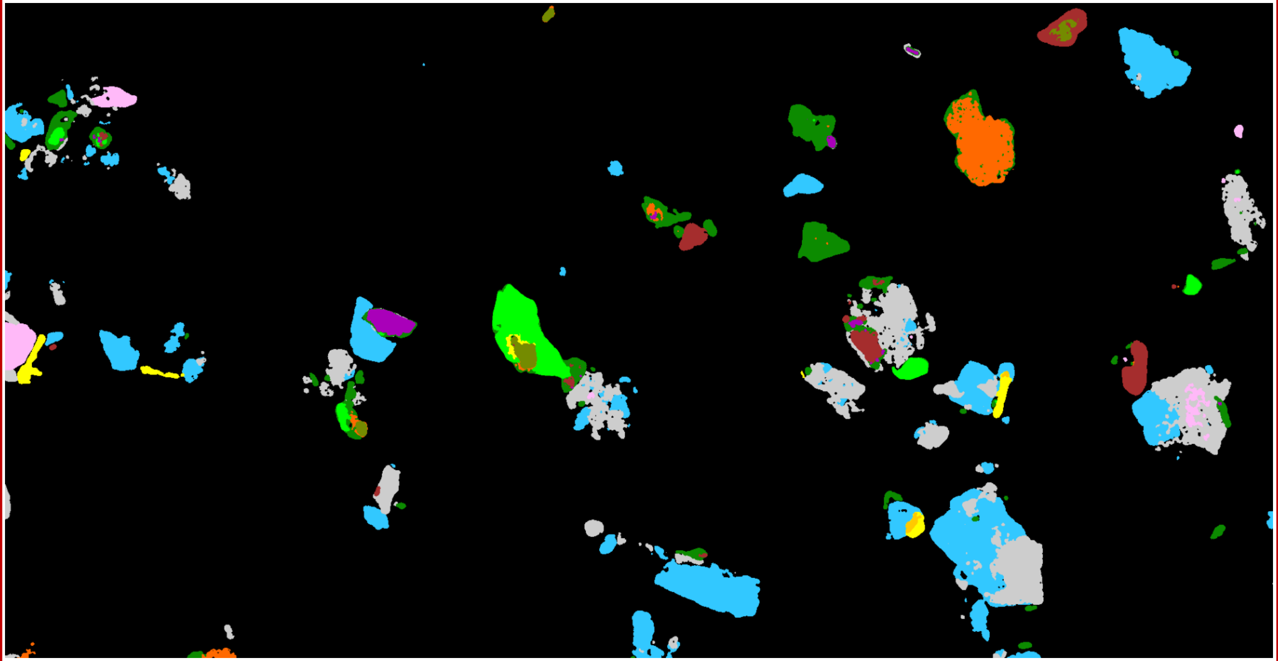

Output AH

Plagioclase (albite to anorthite)	15.6%	"Al"+"Ca"+"Na"
Glass (all compositions)	14.7%	"K"+"Si"
Pyroxene (OPX) or Olivine	9.74%	"Fe"+"Mg"
Plagioclase (albite to anorthite)	8.22%	"Al"+"Na"
Glass (all compositions)	6.34%	"K"+"Na"
CPX	4.66%	"Ca"+"Mg"+"Fe"
Mg-Chloride?	4.37%	"Cl"+"Mg"
Plagioclase (albite to anorthite)	3.51%	"Al"
Fe-Ti oxide	3.31%	"Fe"+"Ti"
Anhydrite	1.90%	"Ca"+"S"
Crystalline silica	1.67%	"Si"
Pyrite and/or Fe-chloride	1.34%	"Cl"+"S"
Mica	0.90%	"K"+"Mg"

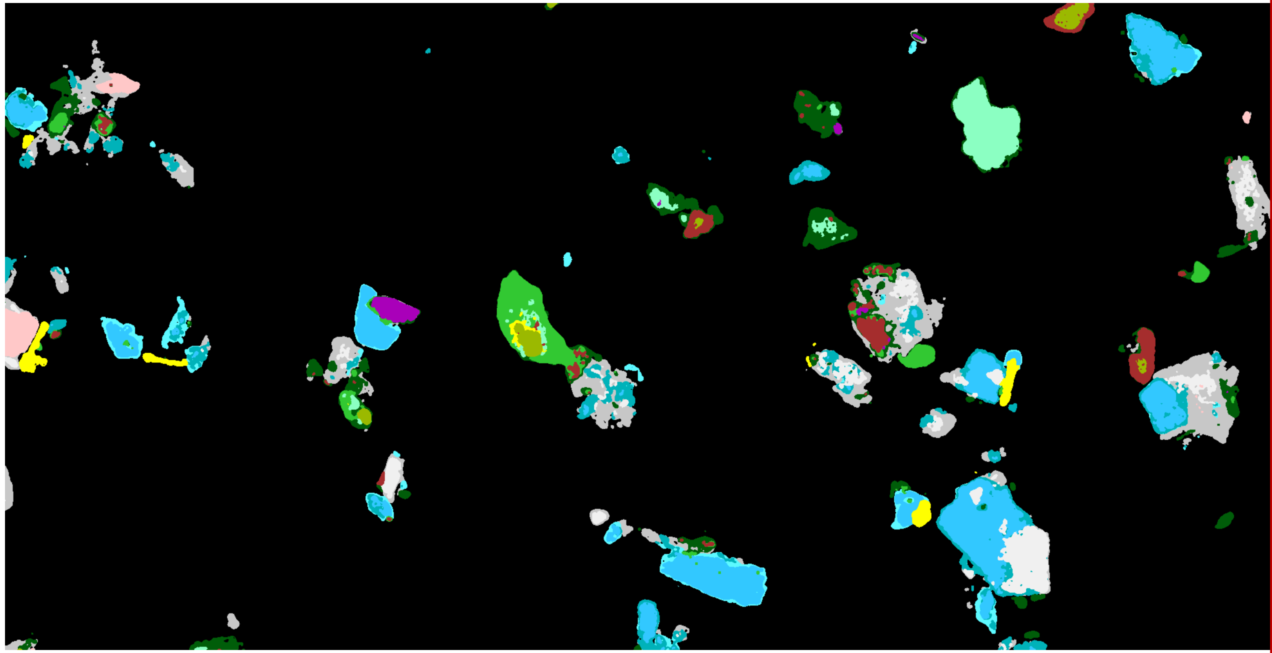

Map 3:

Multi-elemental EDS map

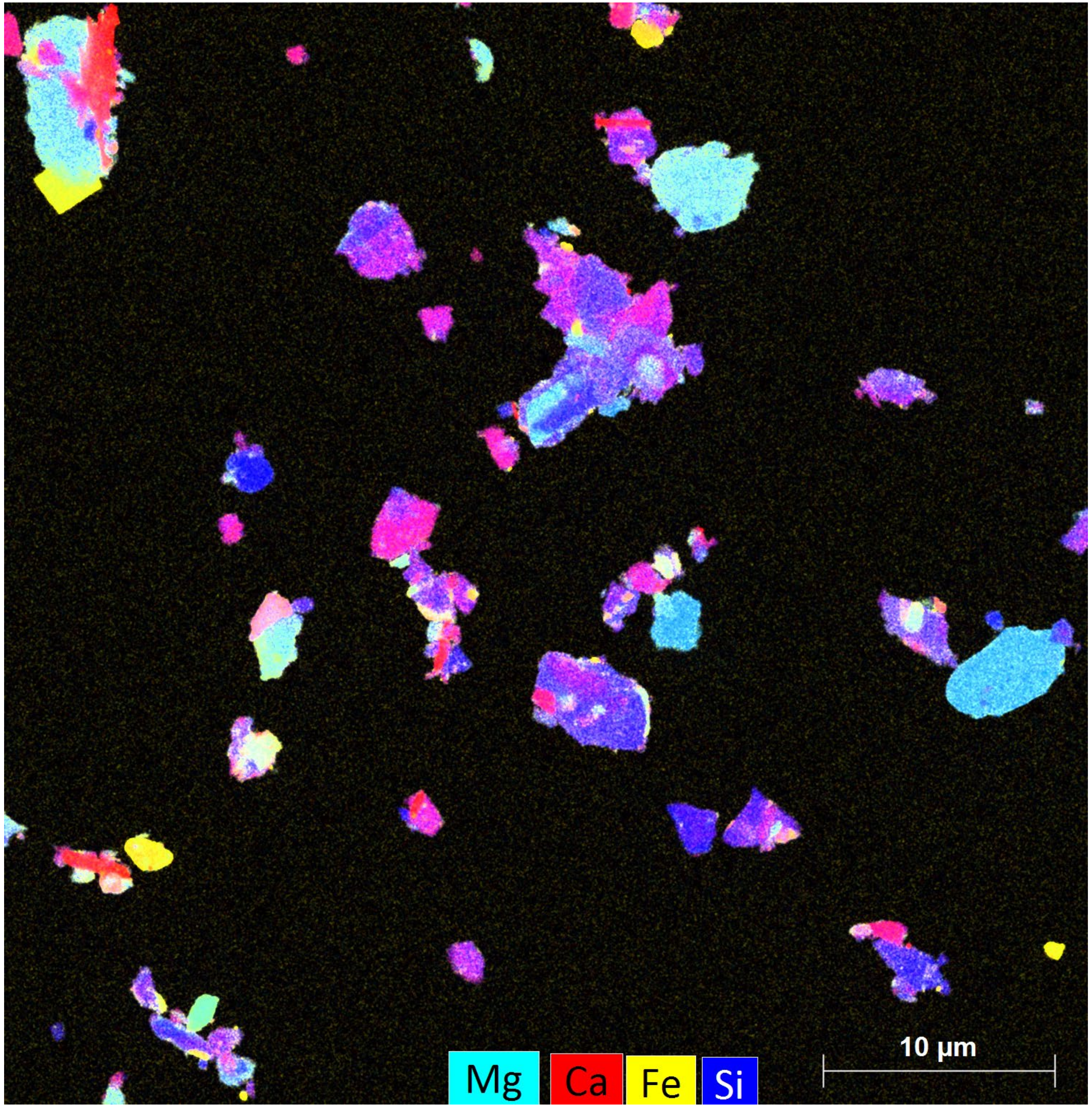

Map 3: Phase maps

Output JE

Phase	Area %	Element(s)	Area % normalized to 100%
Plagioclase (albite to anorthite)	24.6%	"Al"+"Ca"+"Na"	31.2
Pyroxene (OPX) + Olivine	24.6%	"Mg"	31.2
Glass (all compositions)	16.1%	"K"	20.5
CPX	4.05%	"Ca"+"Mg"	5.1
Anhydrite	3.88%	"Ca"+"S"	4.9
Fe-Ti oxide	3.12%	"Fe"+"Ti"	4.0
Natroalunite	1.13%	"Al"+"S"	1.4
Chloride of some type	0.88%	"Cl2"	1.1
Pyrite and/or Fe-chloride	0.39%	"Cl2"+"S"	0.5

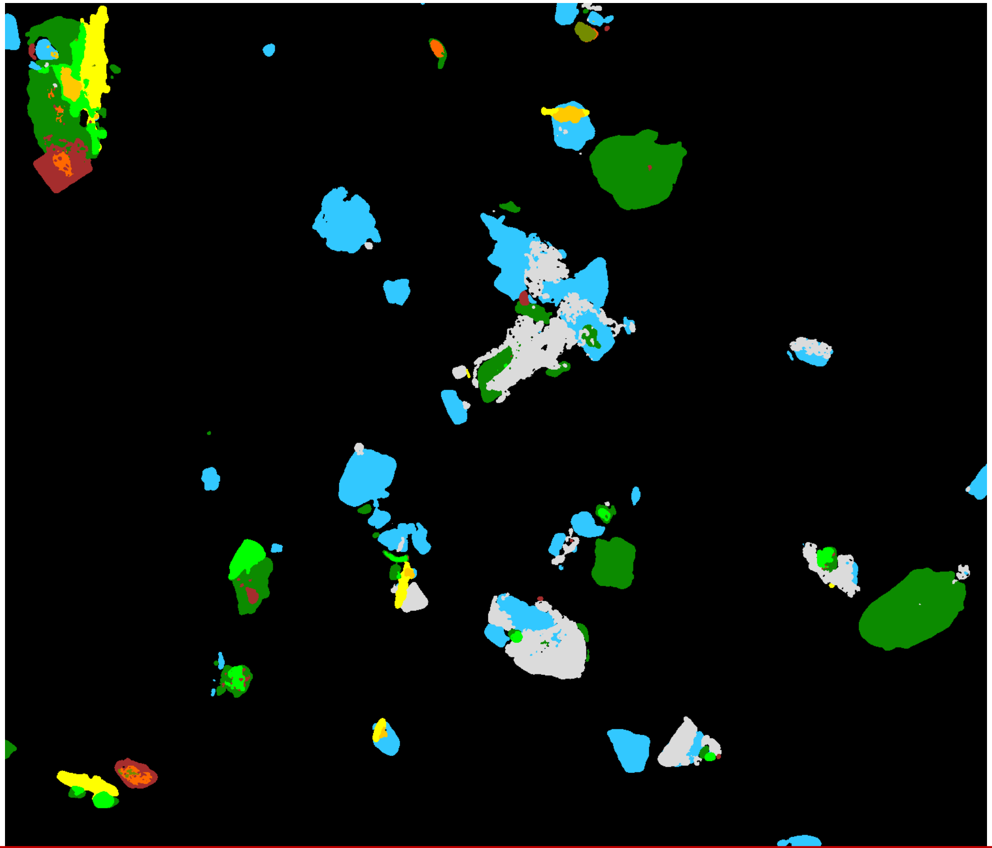

Output AH

Phase	Area %	Element(s)
Plagioclase (albite to anorthite)	21.8%	"Al"+"Ca"+"Na"
Pyroxene (OPX) or Olivine	17.9%	"Fe"+"Mg"
Glass (all compositions)	13.9%	"K"+"Si"
Crystalline silica	8.33%	"Si"
Anhydrite	7.16%	"Ca"+"S"
Fe-Ti oxide	5.24%	"Fe"+"Ti"
Mg-Chloride?	4.72%	"Cl"+"Mg"
CPX	3.89%	"Ca"+"Mg"+"Fe"

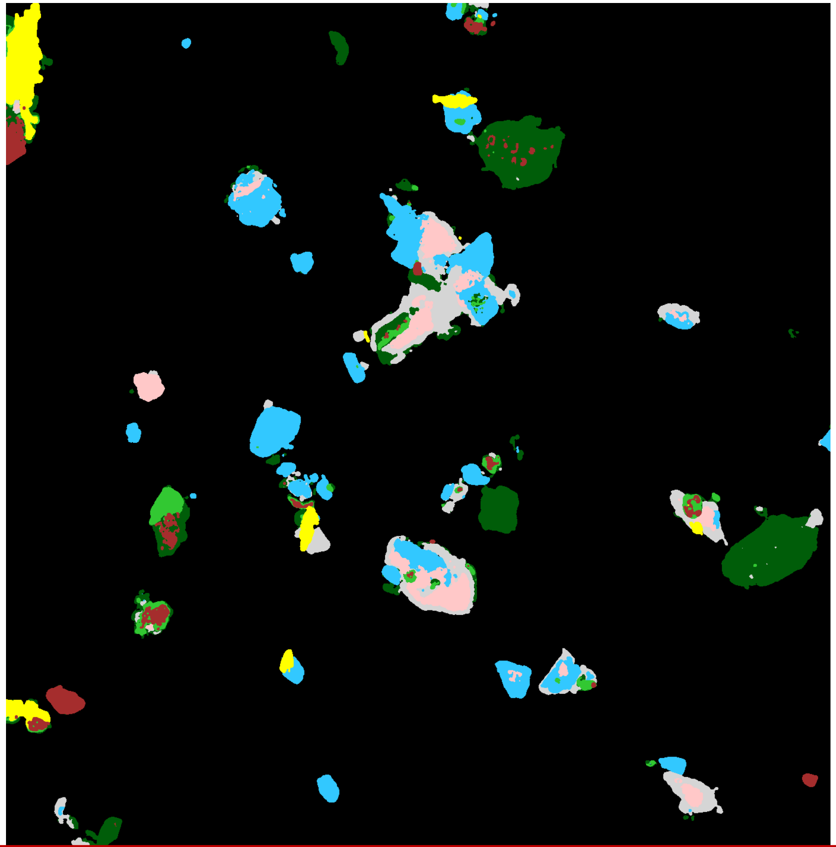

Map 4:

Multi-elemental EDS map

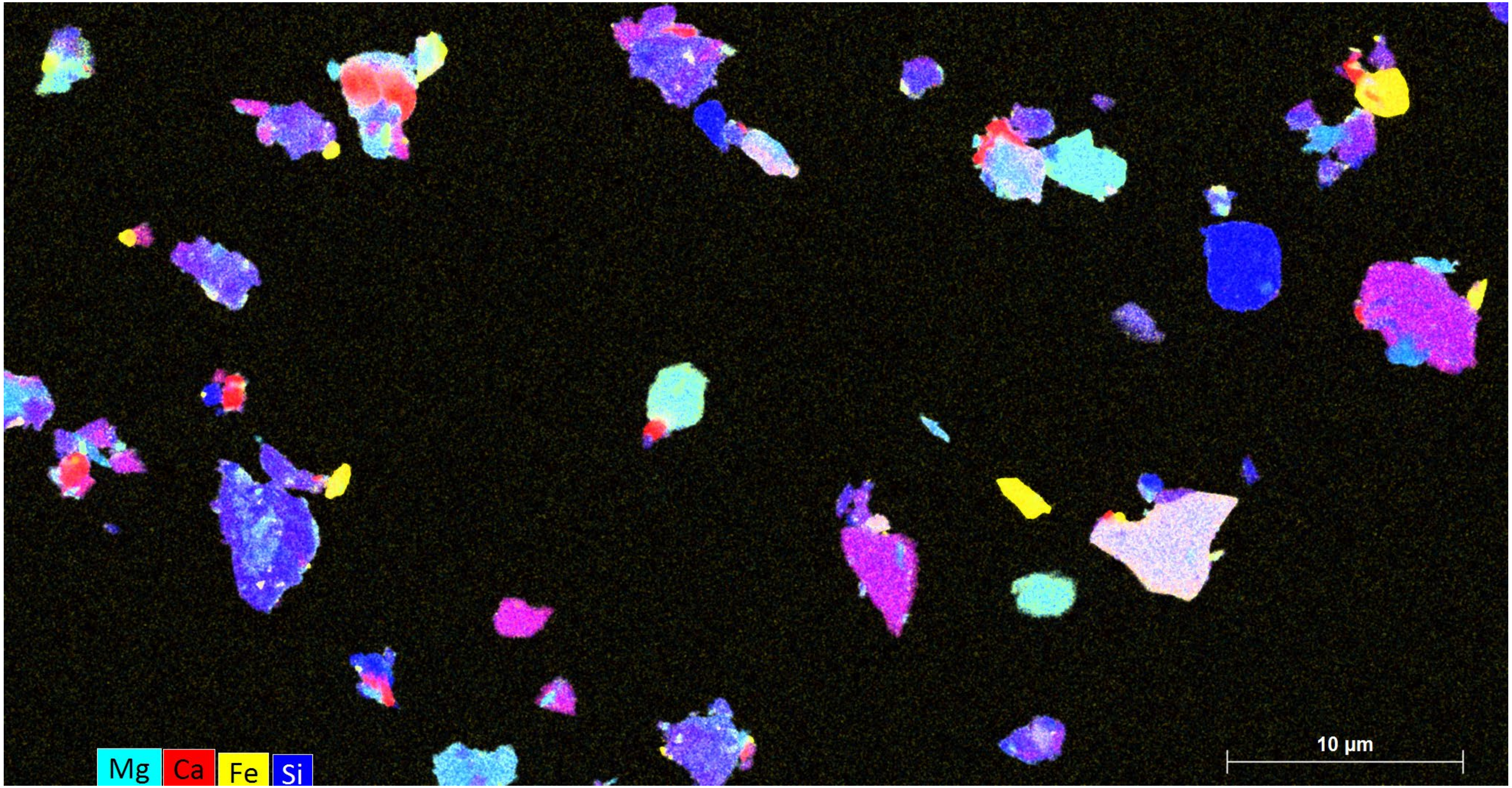

Map 4: Phase maps

Output JE

			Area % normalized to 100%
Plagioclase (albite to anorthite)	27.9%	"Al"+"Ca"+"Na"	38.4
Glass (all compositions)	12.7%	"K"	17.6
Pyroxene (OPX) + Olivine	8.40%	"Mg"	11.5
CPX	8.34%	"Ca"+"Mg"	11.5
Crystalline silica	5.14%	"Si"	7.1
Fe-Ti oxide	3.67%	"Fe"+"Ti"	5.0
Anhydrite	2.66%	"Ca"+"S"	3.7
Chloride of some type	2.01%	"Cl2"	2.8
Natroalunite	1.04%	"Al"+"S"	1.4
Pyrite and/or Fe-chloride	0.73%	"Cl2"+"S"	1.0

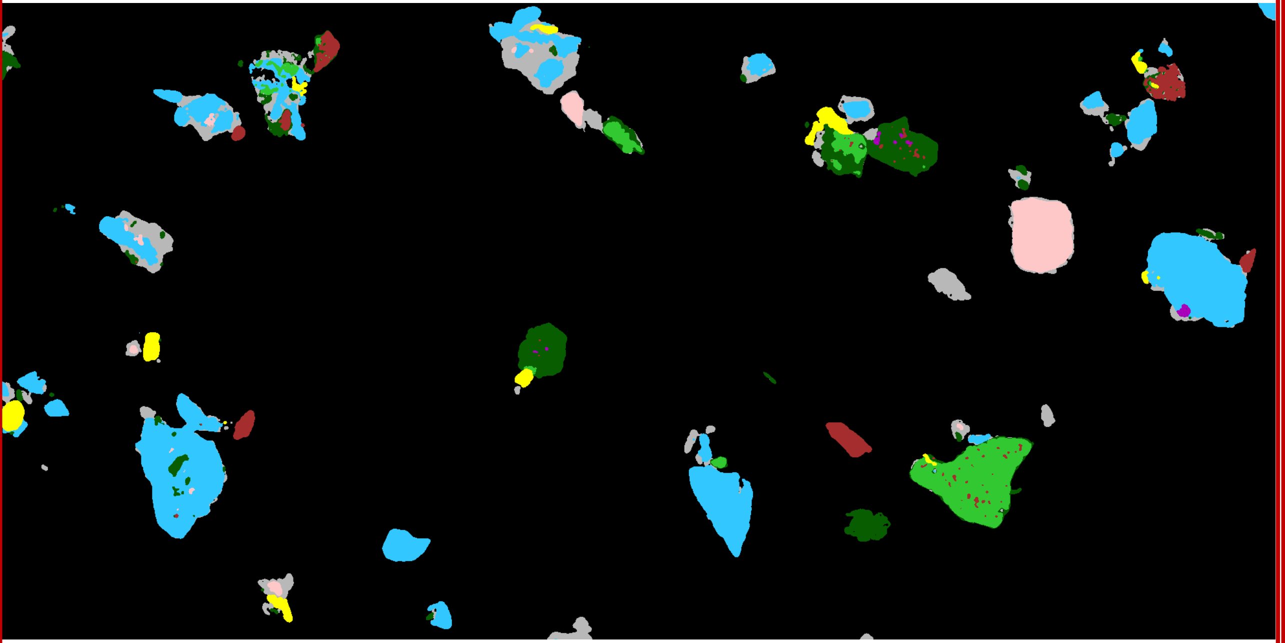

Output AH

Plagioclase (albite to anorthite)	33.9%	"Al"+"Ca"+"Na"
Glass (all compositions)	13.7%	"K"+"Si"
Pyroxene (OPX) or Olivine	11.3%	"Fe"+"Mg"
CPX	8.98%	"Ca"+"Mg"+"Fe"
Crystalline silica	5.62%	"Si"
Fe-Ti oxide	4.26%	"Fe"+"Ti"
Anhydrite	3.45%	"Ca"+"S"
Mica	0.28%	"K"+"Mg"

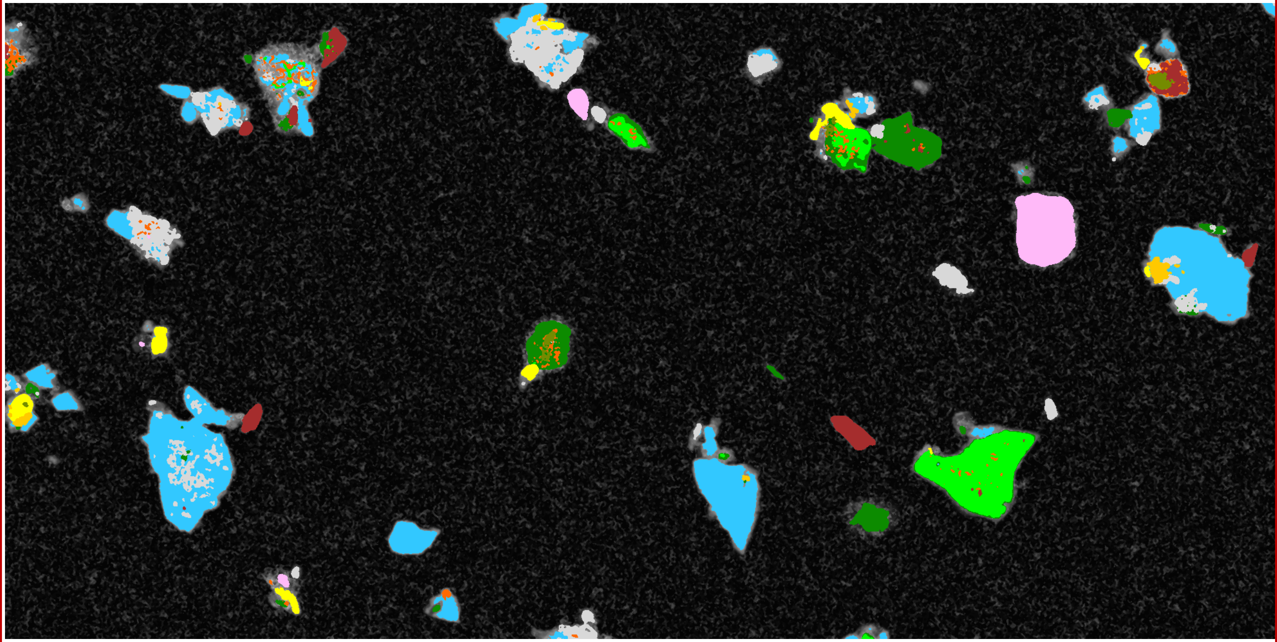

Summary SVA: Mean of maps 1 to 4

Output JE

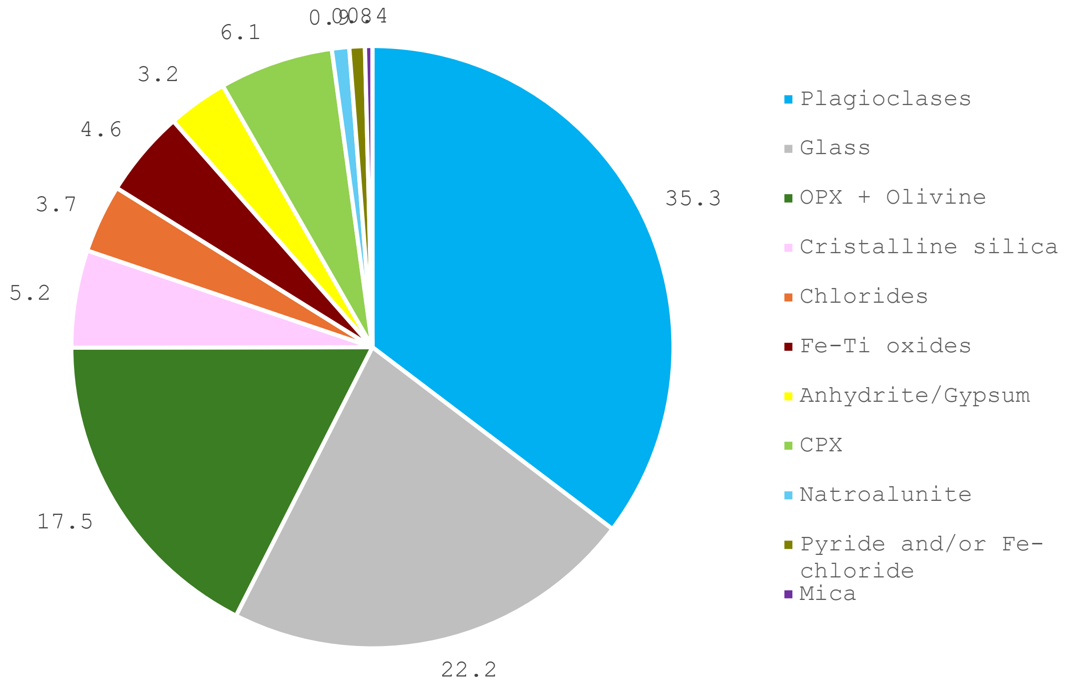

Output AH

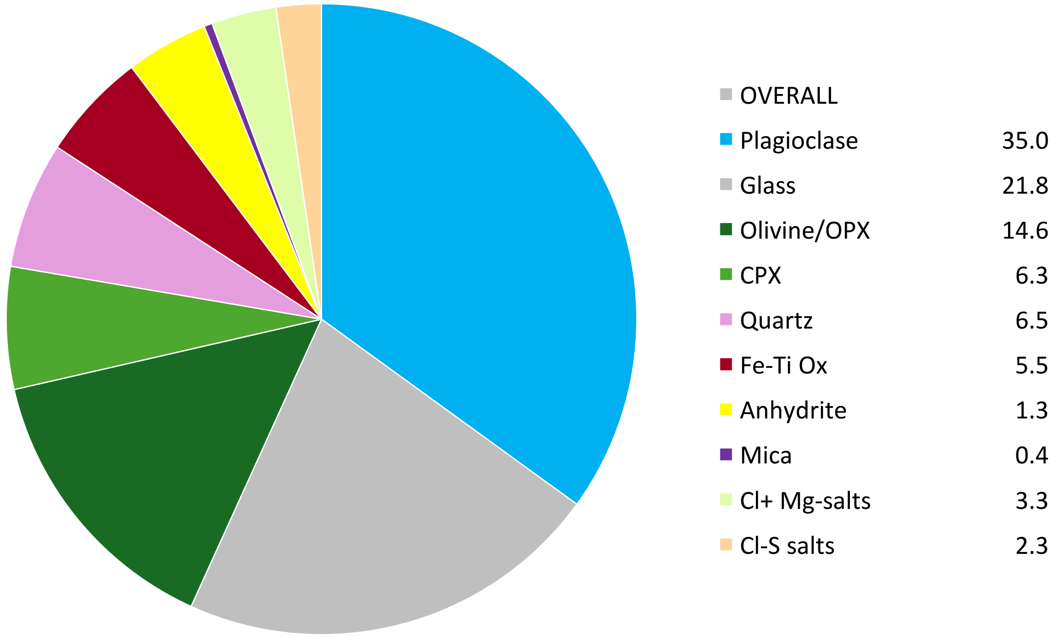

Supplementary file 3:

SE images, EDS maps and EDS spectra for example surface species observed on representative ATD, GDD and SVA particles.

Analytical conditions:

SE imaging: 2 kV, 50 pA, 4 mm working distance

EDS analyses: Xflash detector, 5kV, 3.2nA, 4 mm working distance, 2048px, 2048μs, 30kcps

Arizona Test Dust (ATD) surface species

ATD

Quartz particle – **SE images**

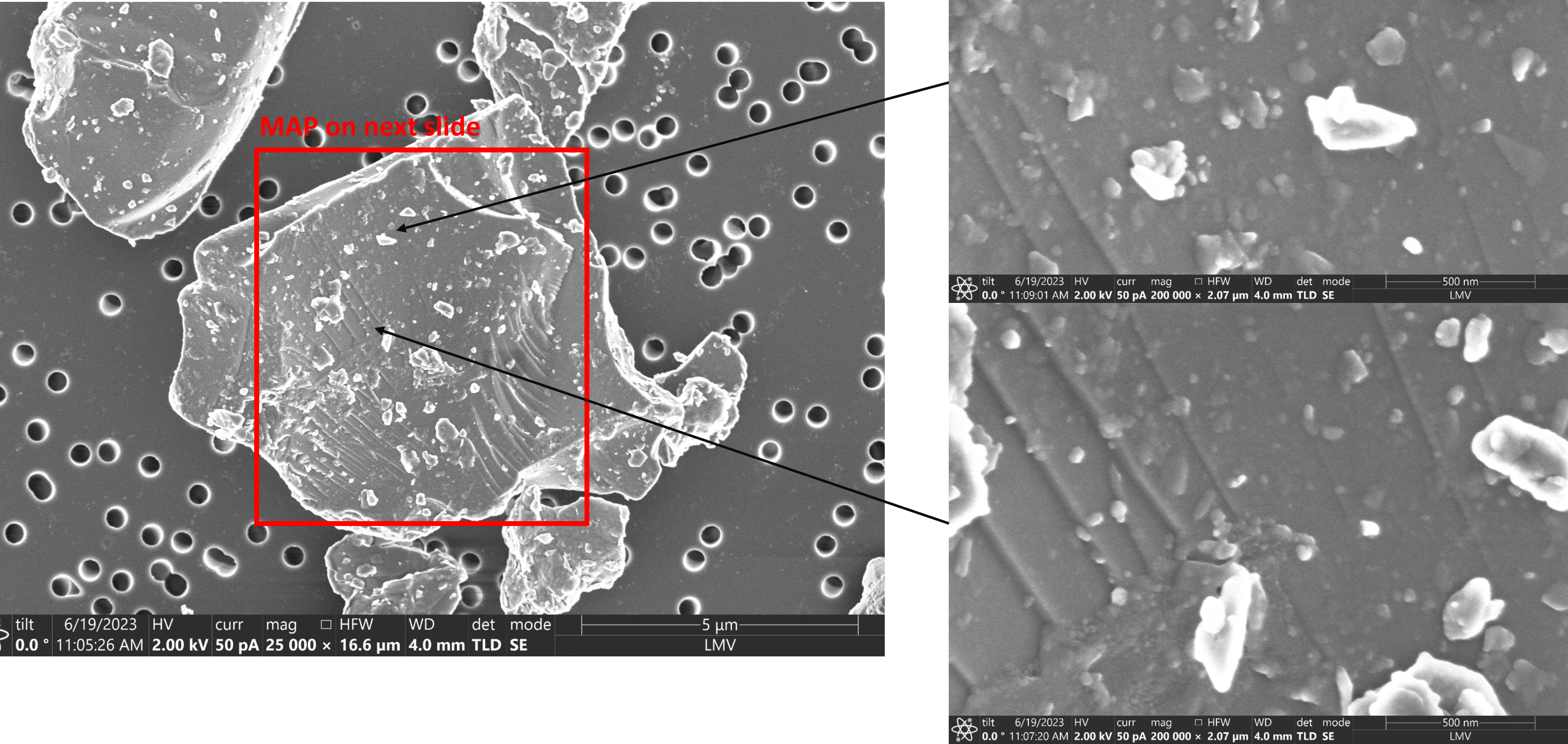

ATD

Quartz particle – **EDS elemental MAPS**

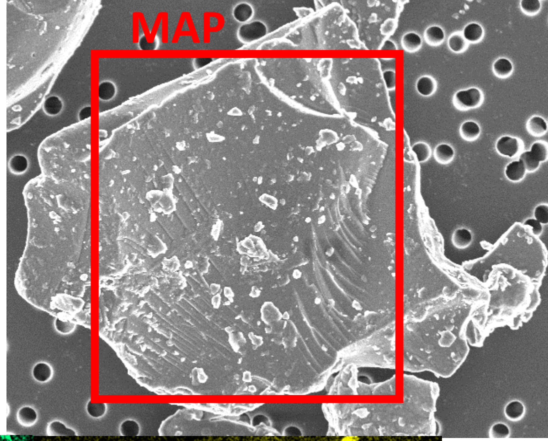

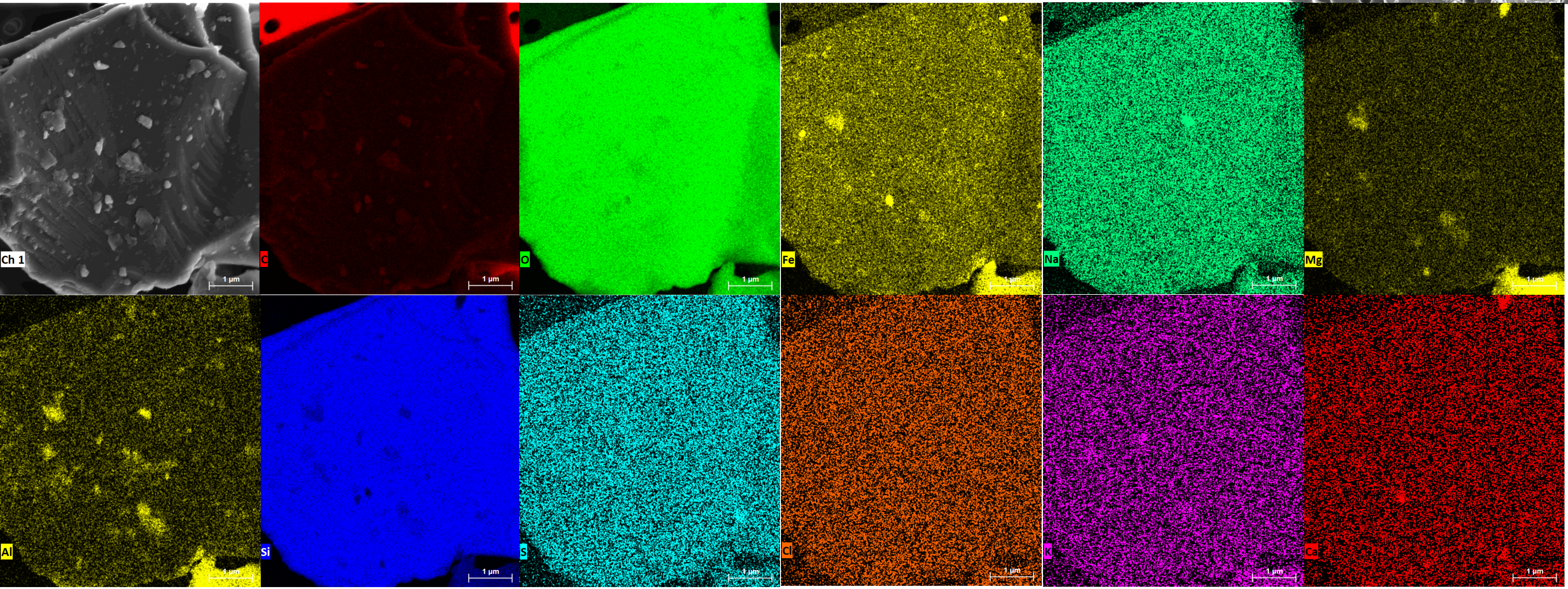

ATD

Quartz particle – EDS spectra

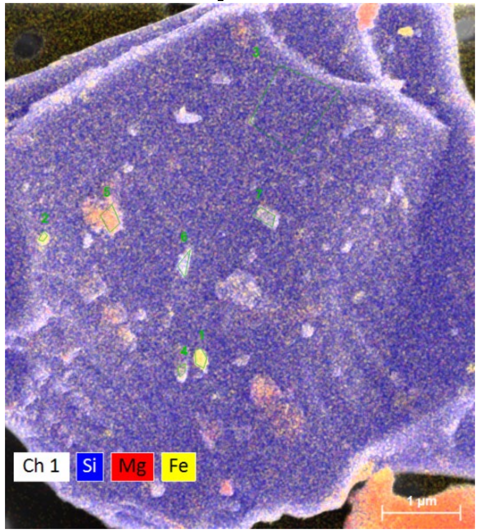

Location on a multi-elemental EDS map of the zones for which the spectra on the right are extracted from.

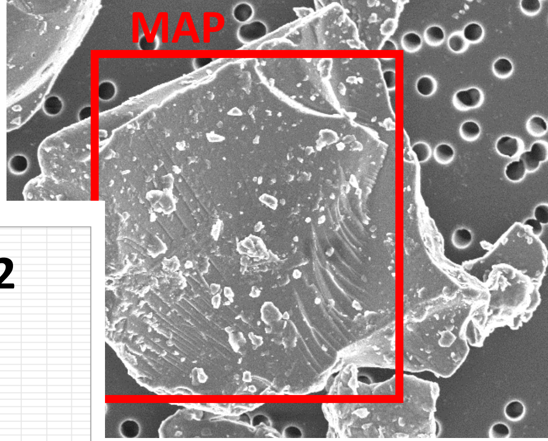

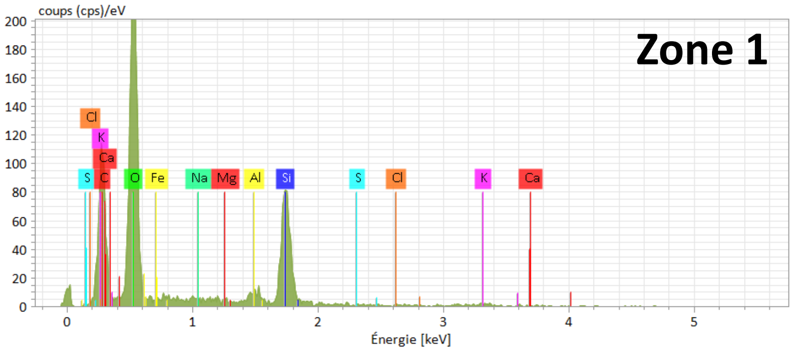

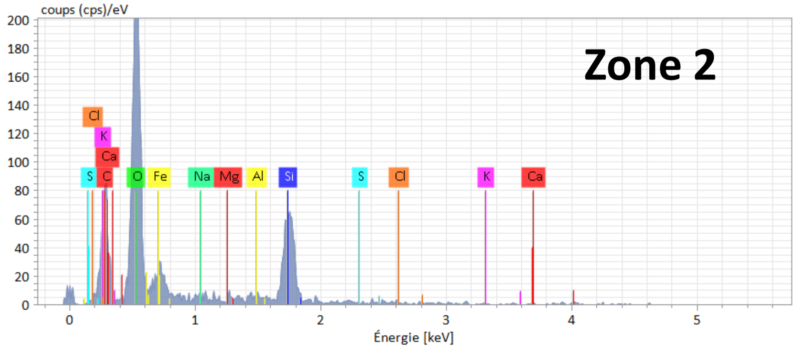

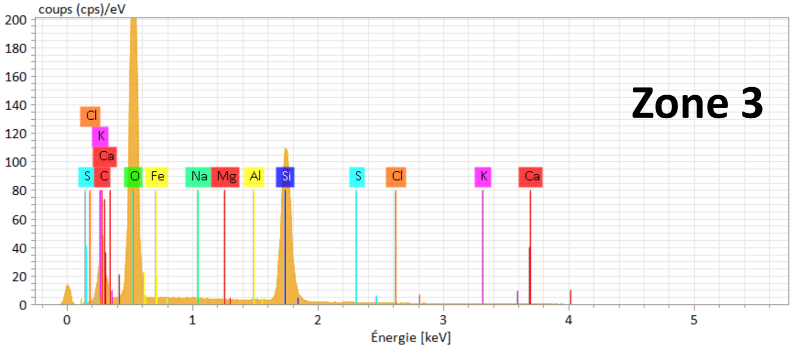

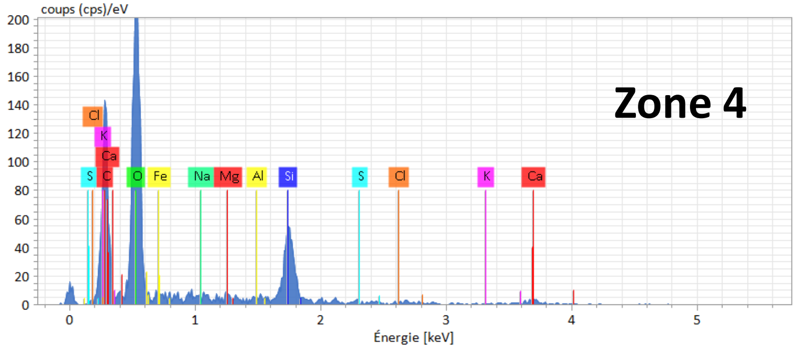

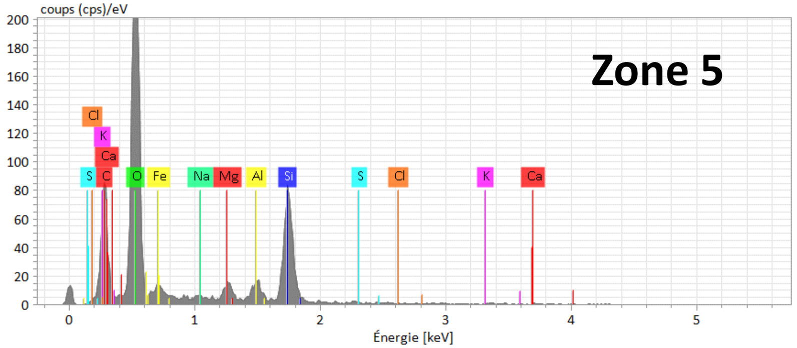

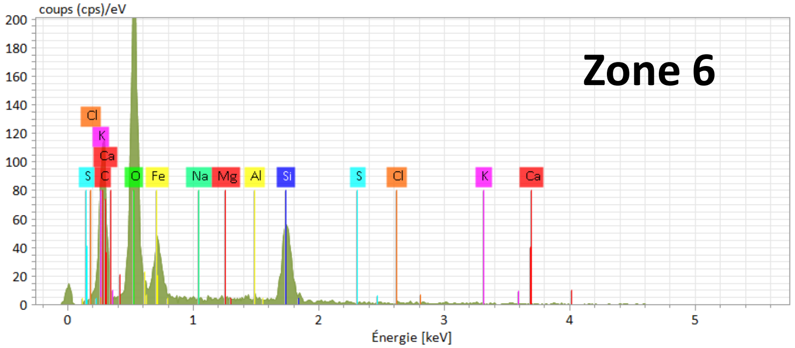

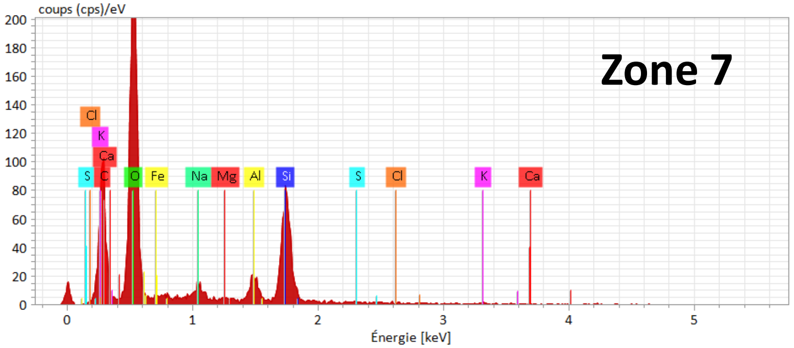

ATD

Mg-calcite particle – **SE images**

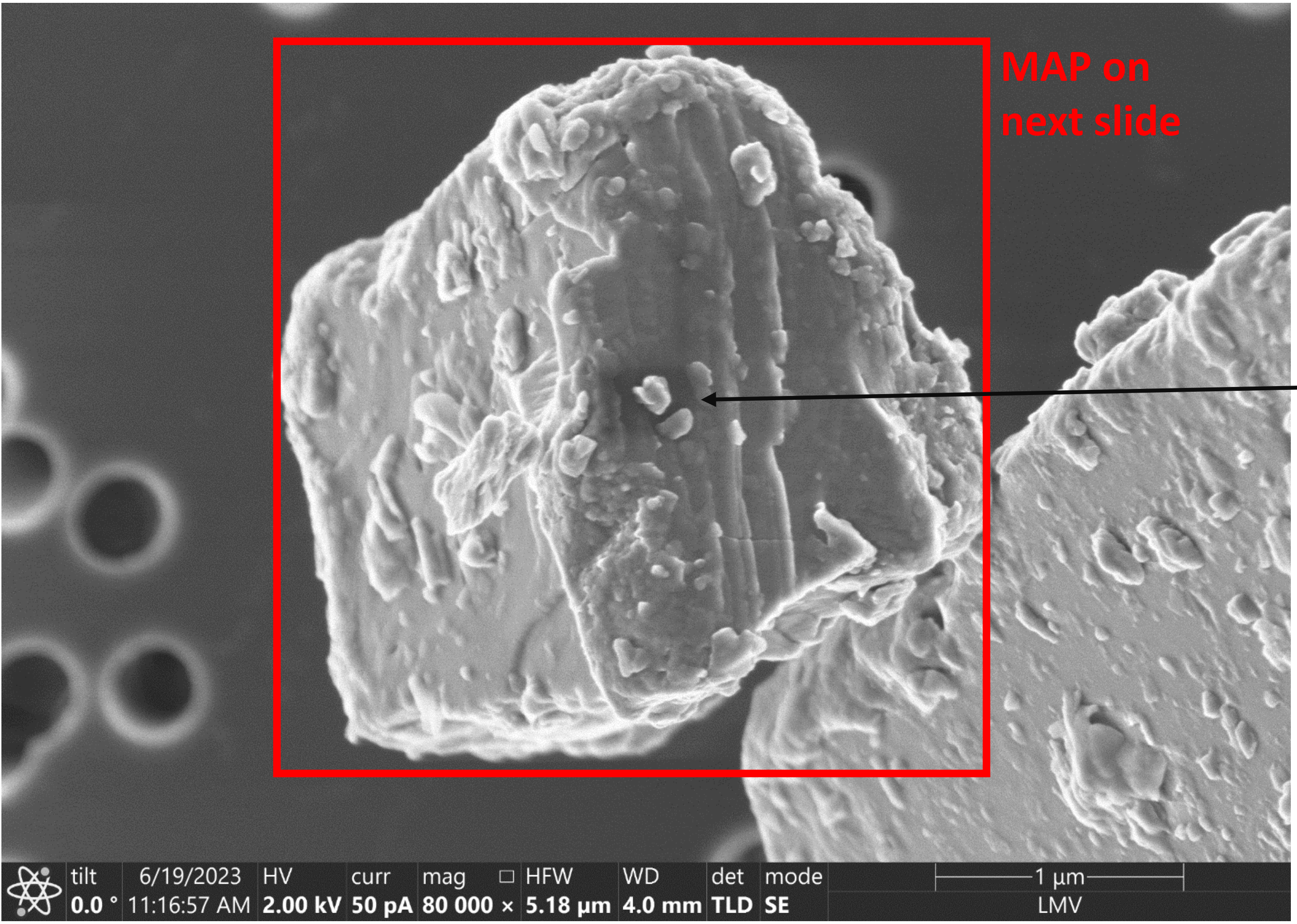

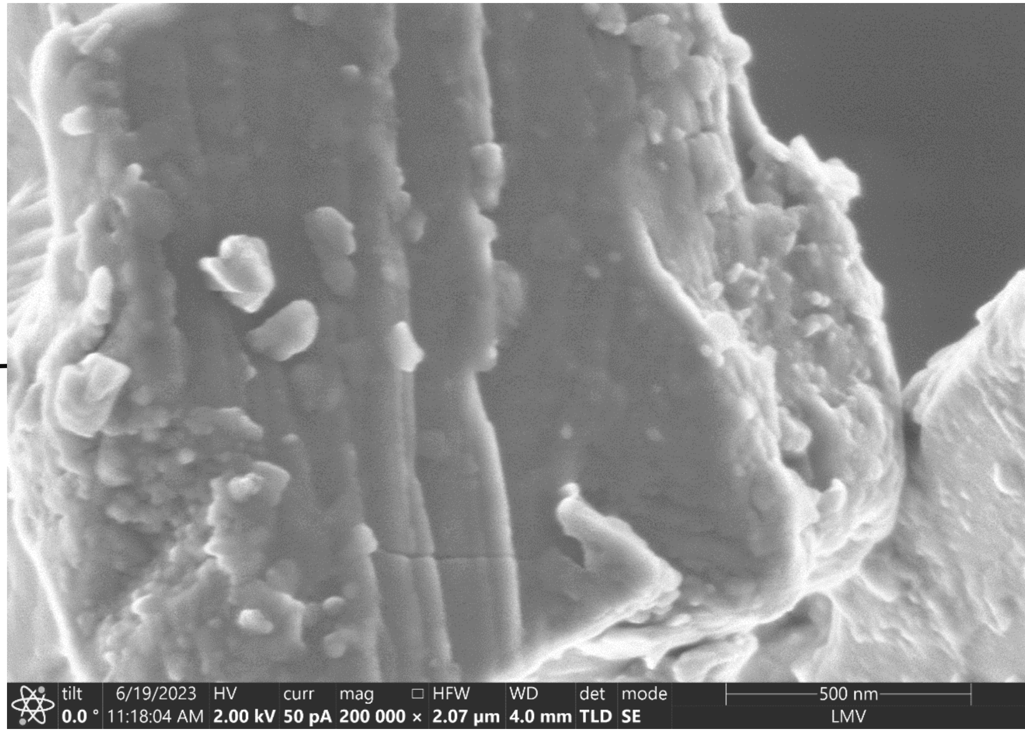

ATD

Mg-calcite particle – **EDS elemental MAPS**

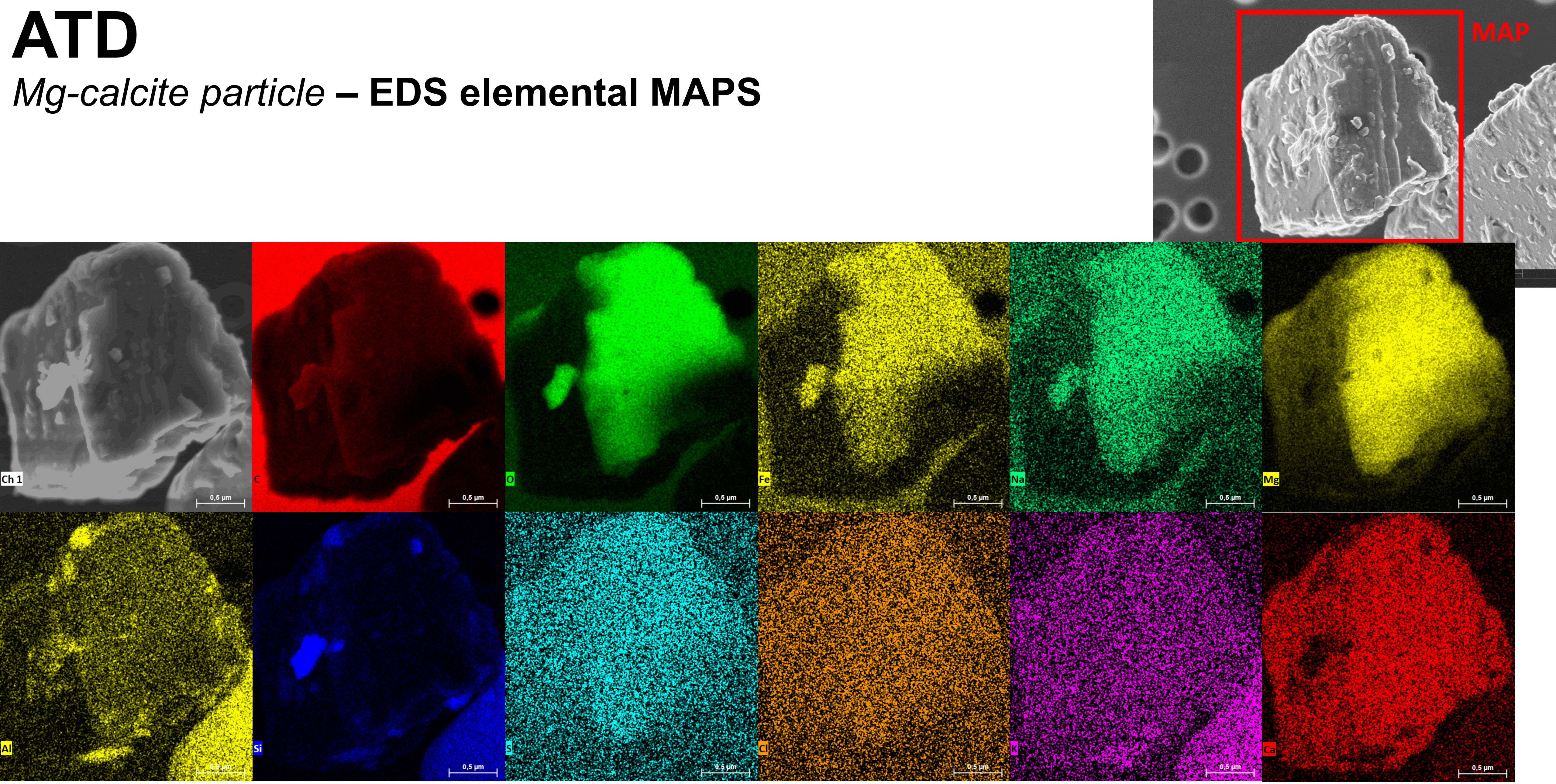

ATD

Mg-calcite particle – EDS spectra

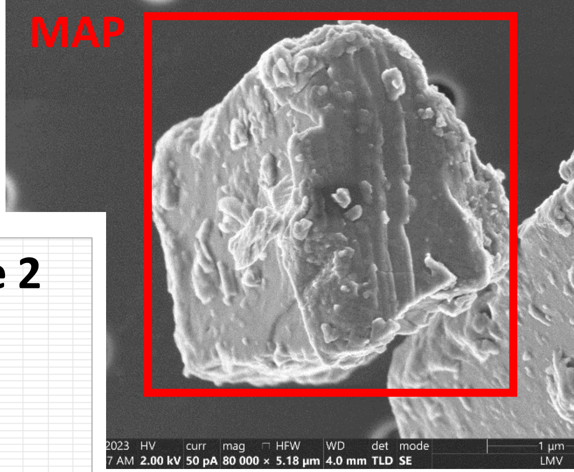

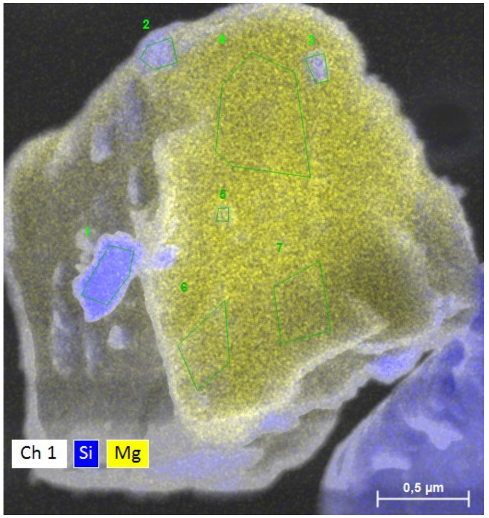

Location on a multi-elemental EDS map of the zones for which the spectra on the right are extracted from.

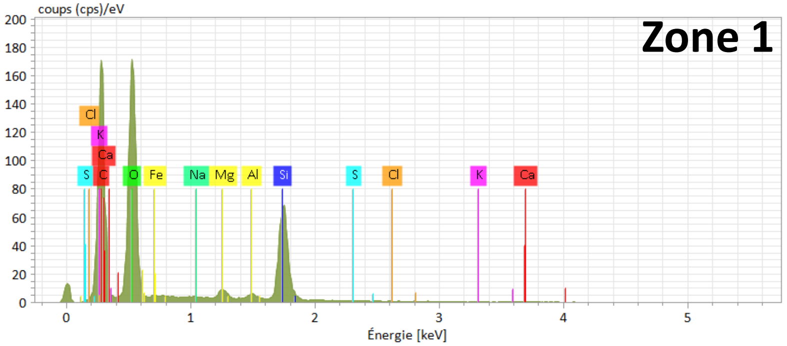

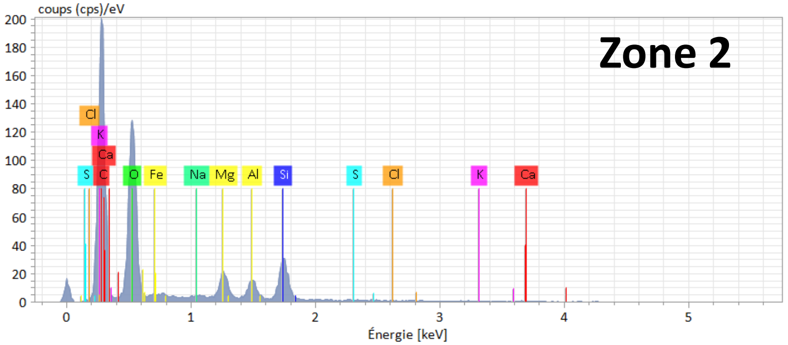

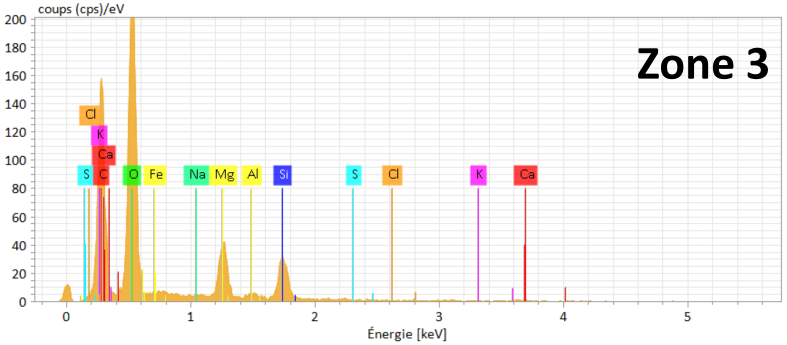

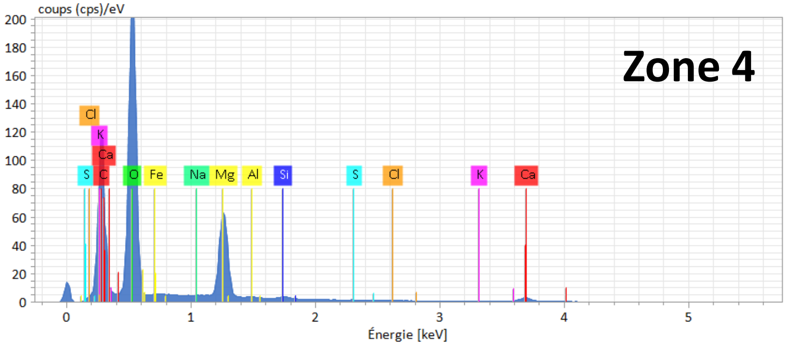

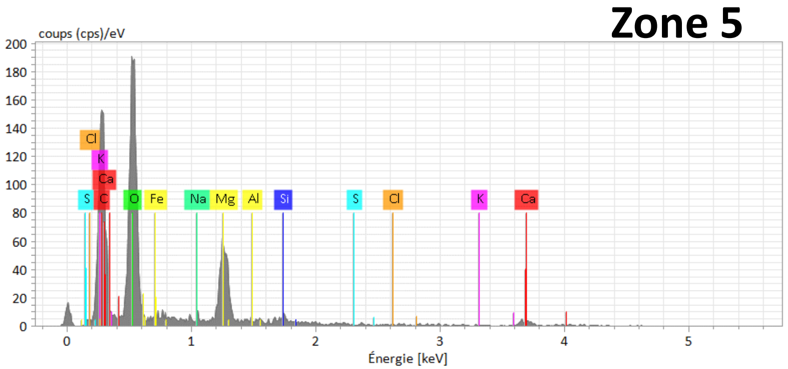

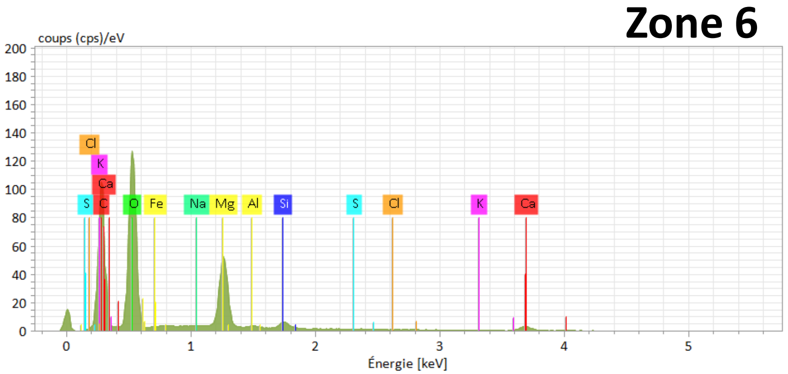

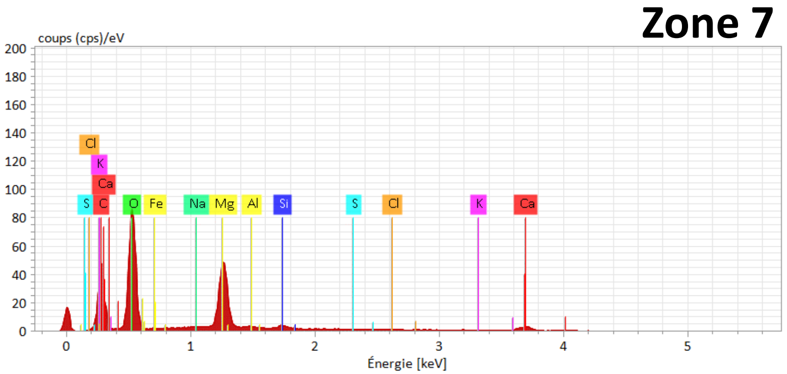

Gobi Desert Dust (GDD) surface species

GDD

Calcite particle – **SE images**

MAP on next slide

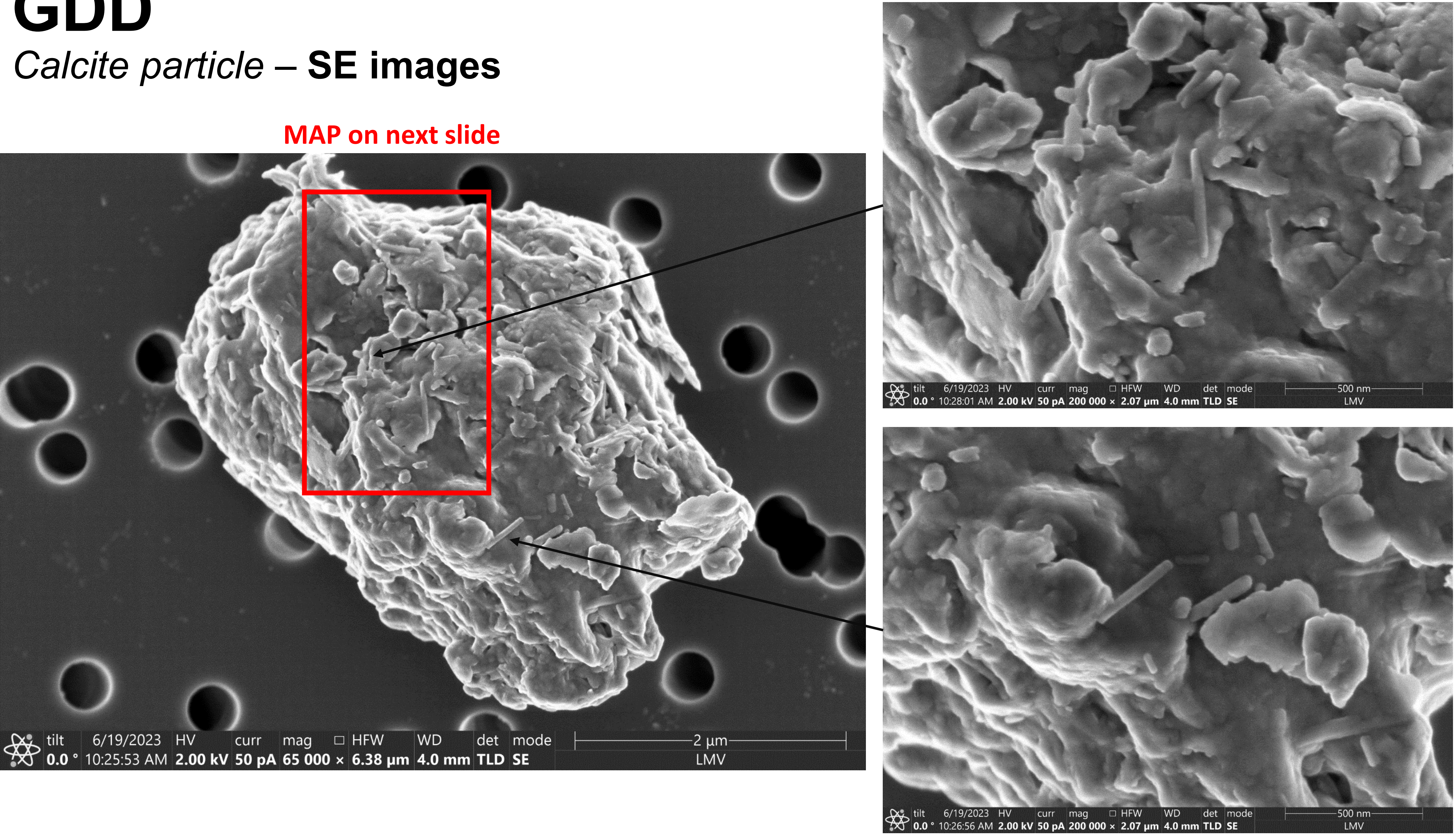

GDD

Calcite particle – **EDS elemental MAPS**

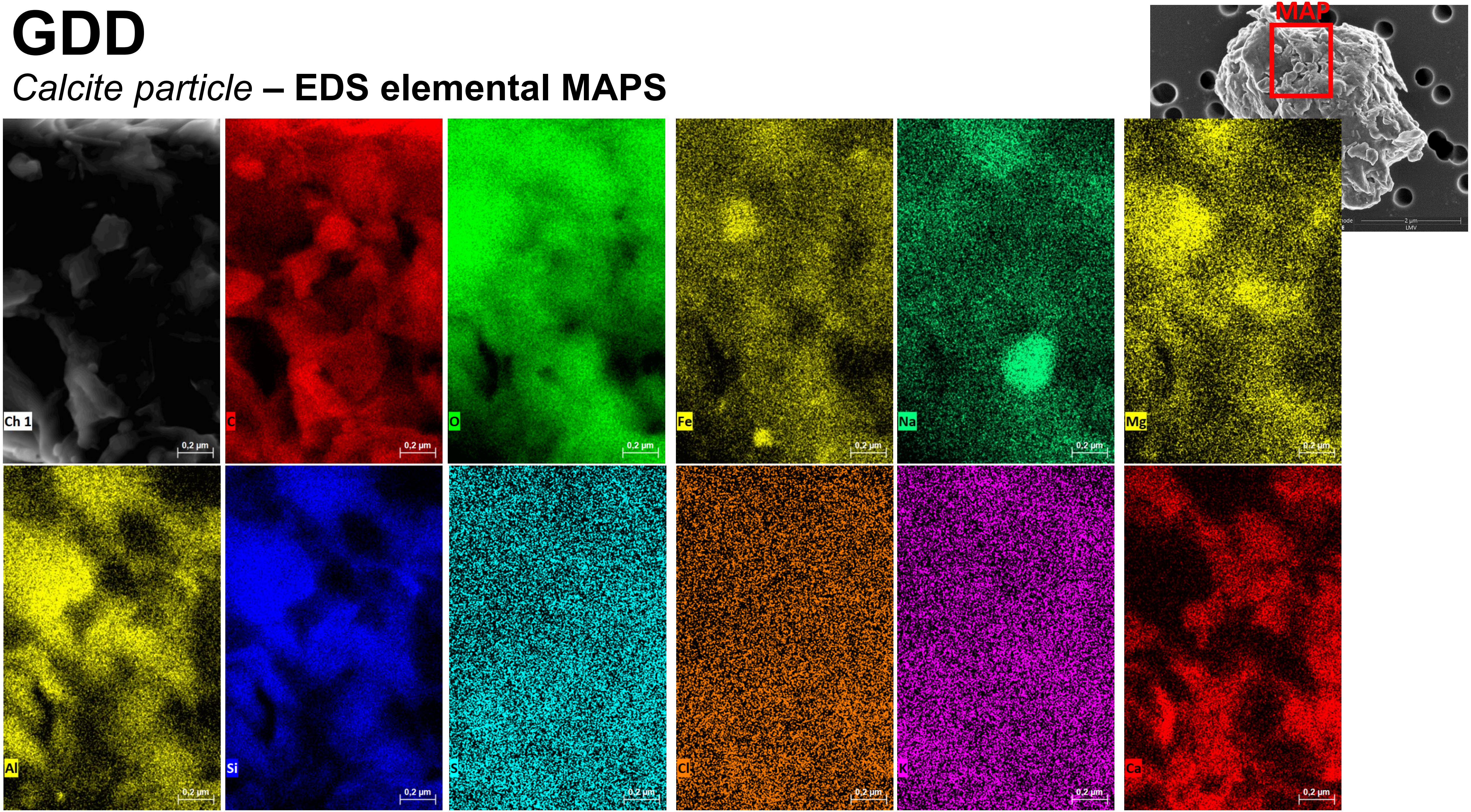

GDD

Calcite particle – EDS spectra

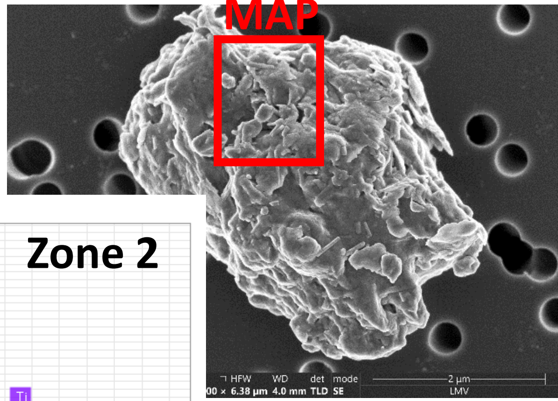

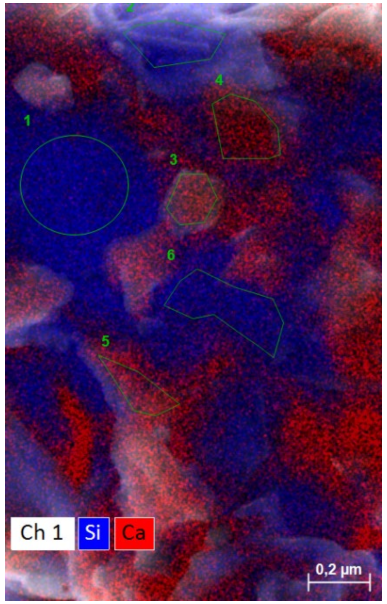

Location on a multi-elemental EDS map of the zones for which the spectra on the right are extracted from.

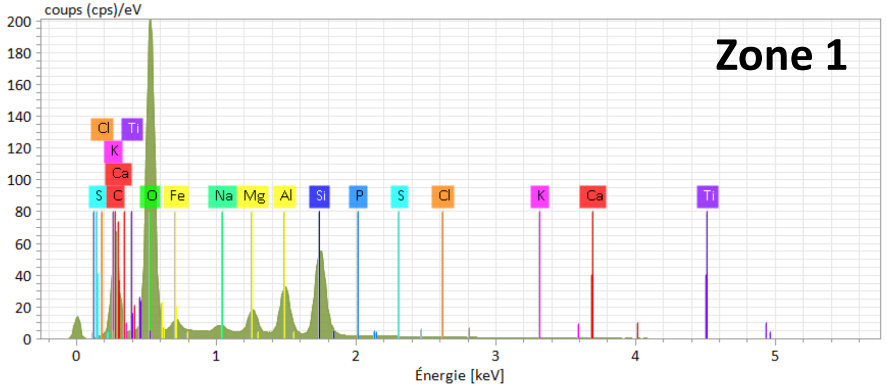

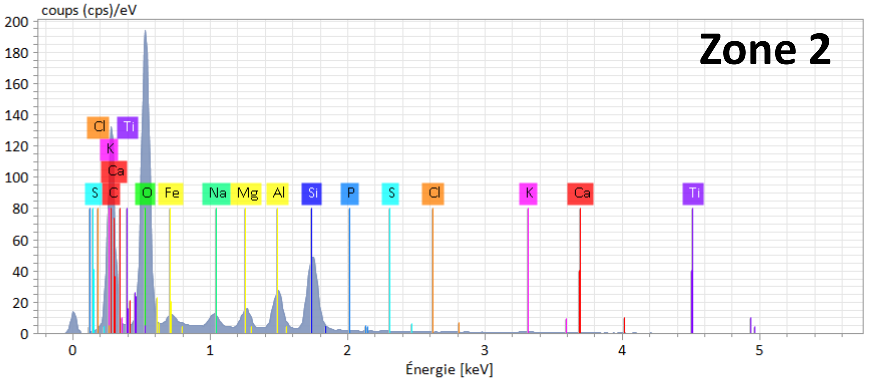

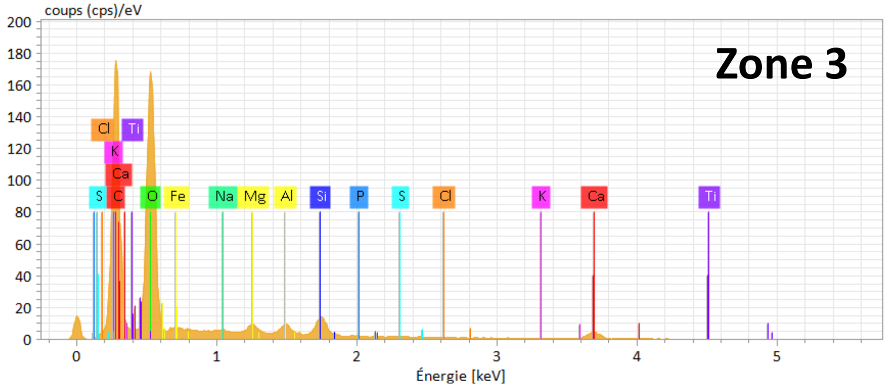

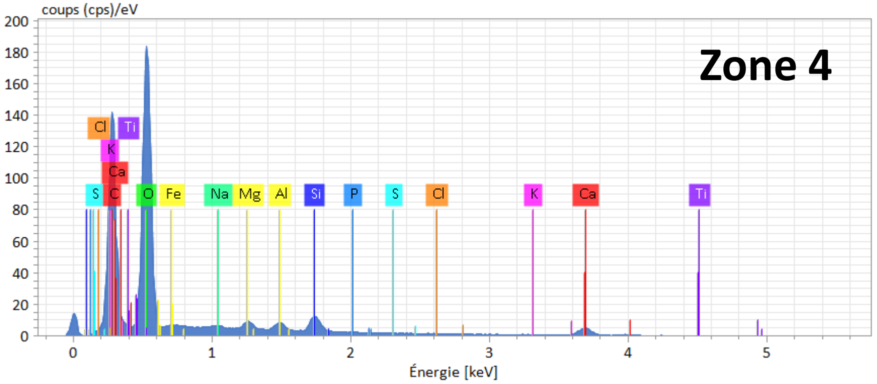

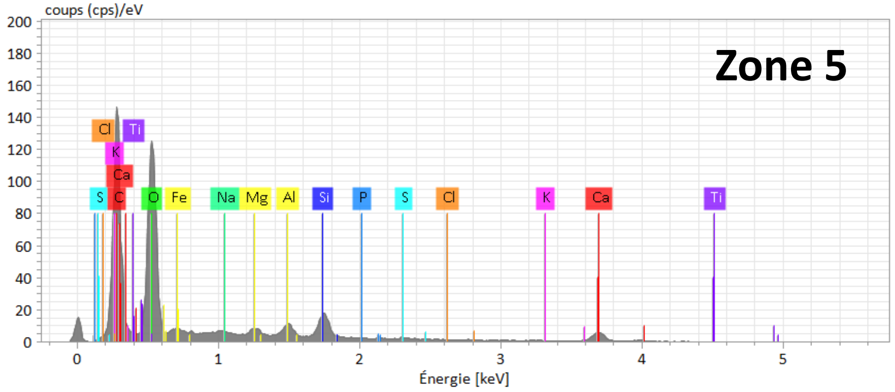

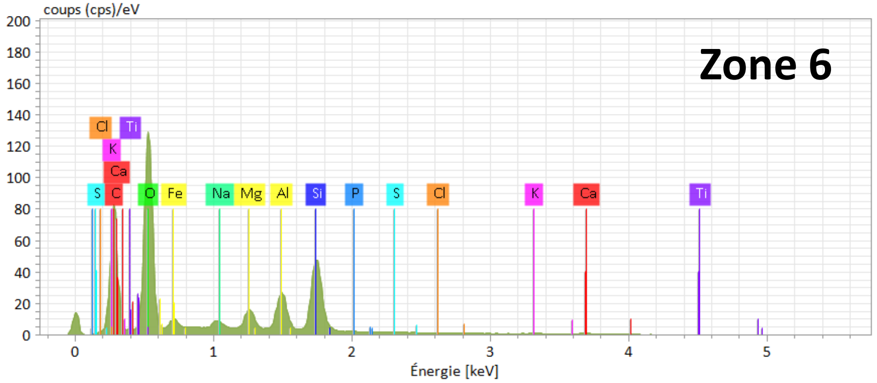

GDD

Mica particle – **SE images**

MAP on next slide

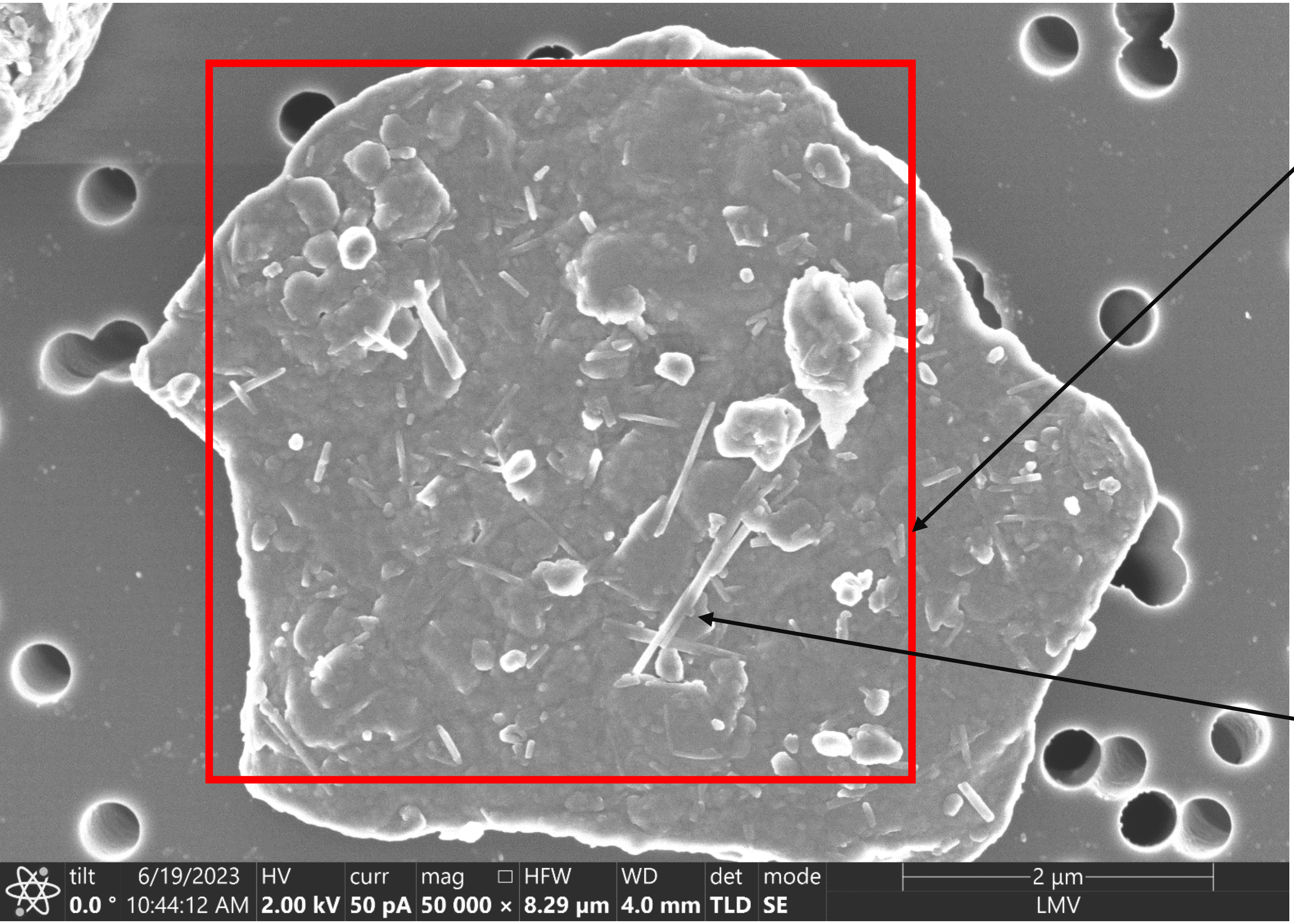

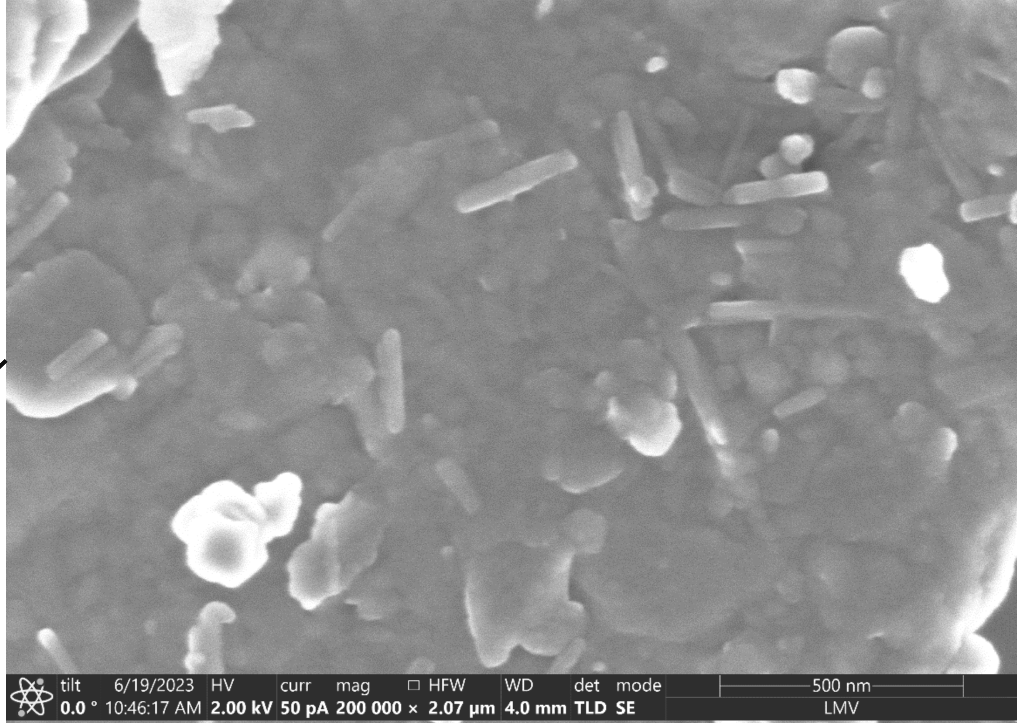

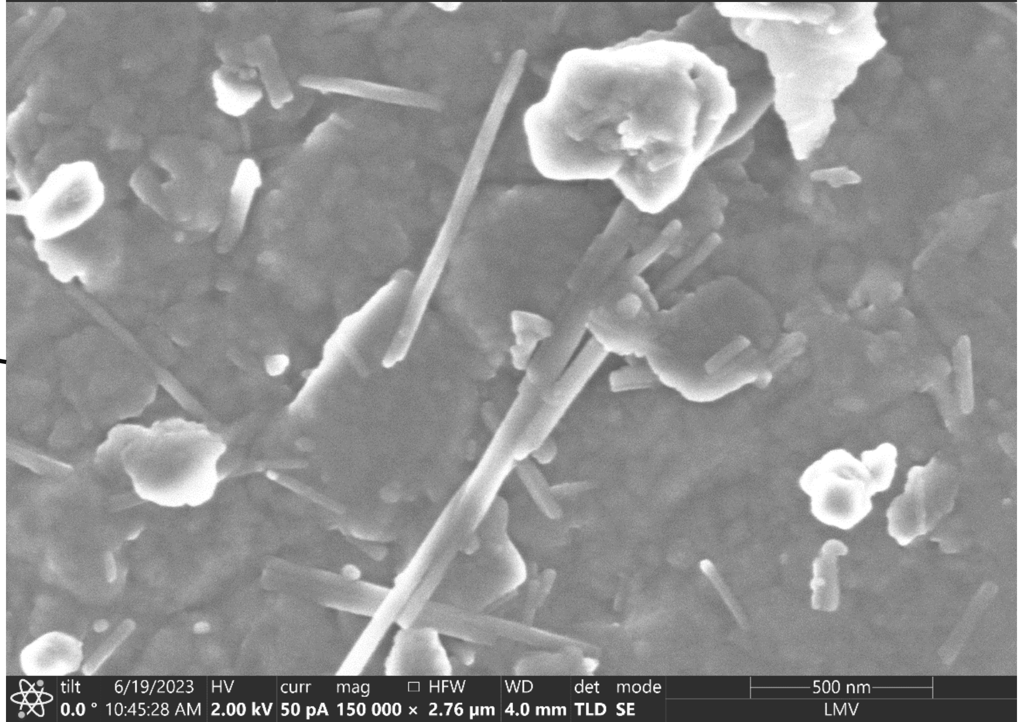

GDD

Mica particle – EDS elemental MAPS

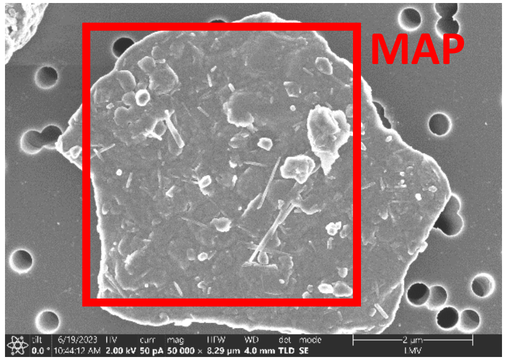

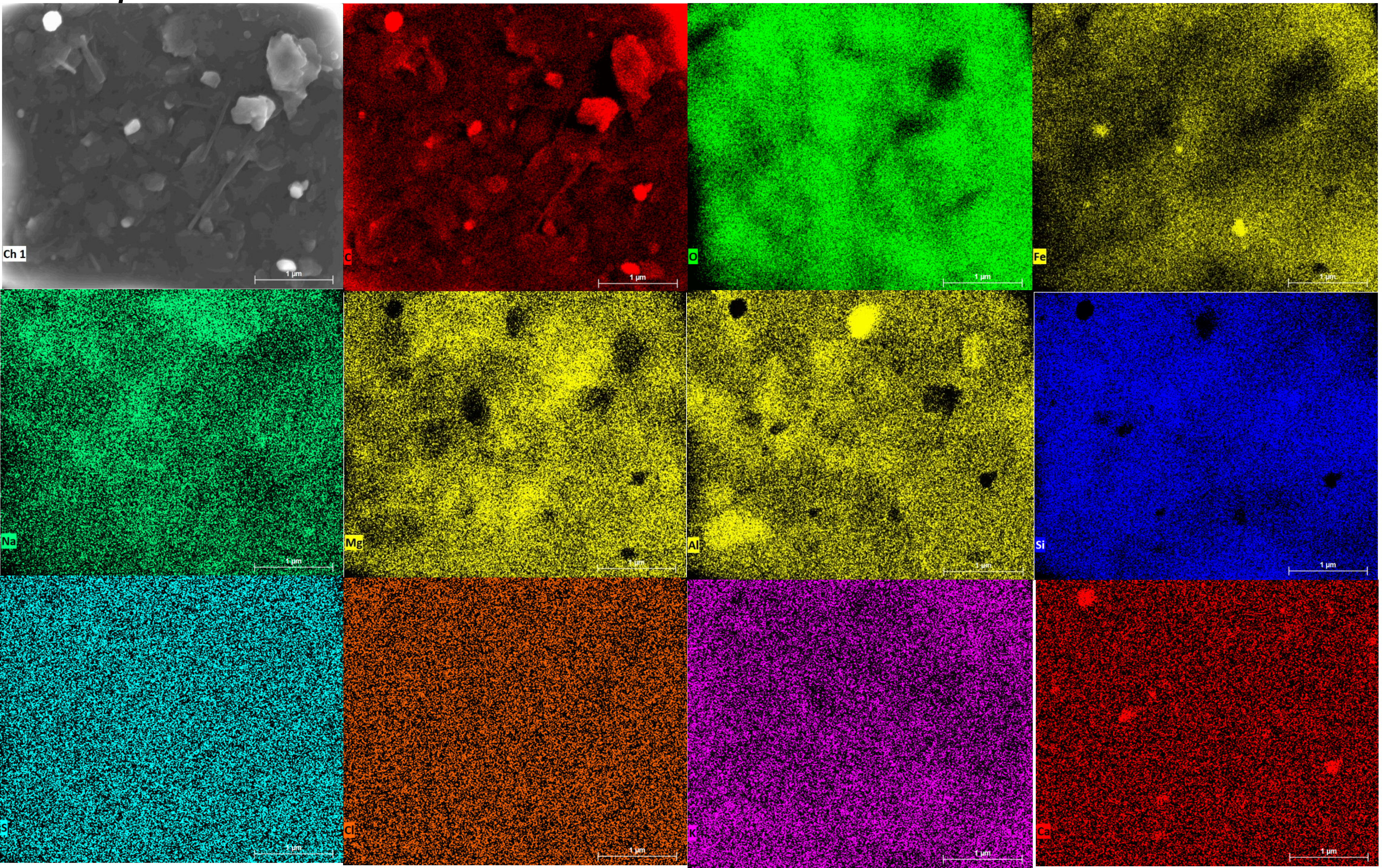

GDD

Mica particle – EDS spectra

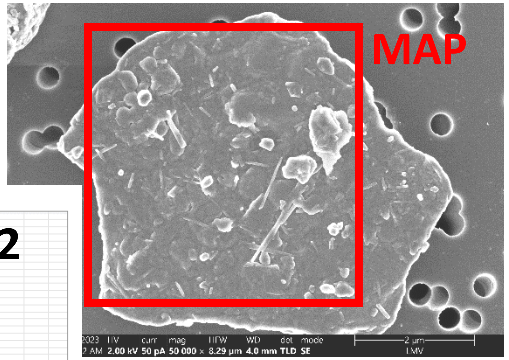

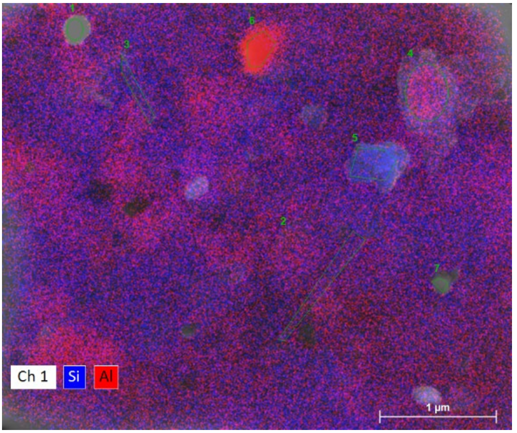

Location on a multi-elemental EDS map of the zones for which the spectra on the right are extracted from.

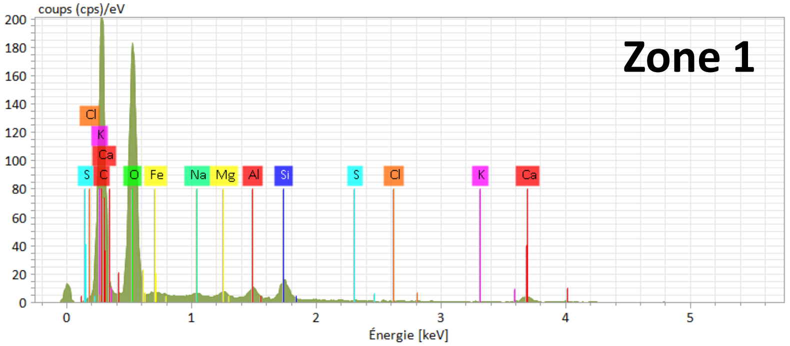

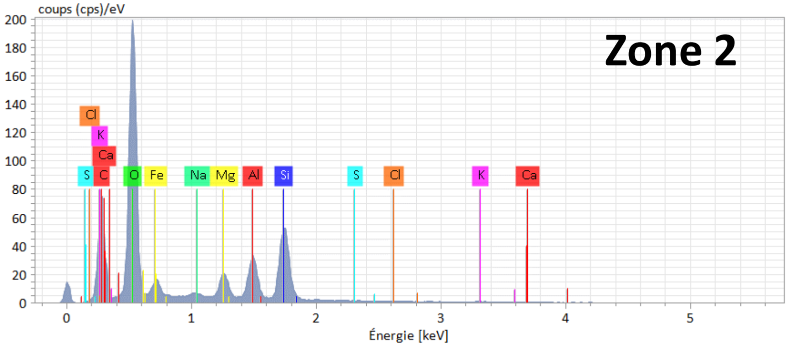

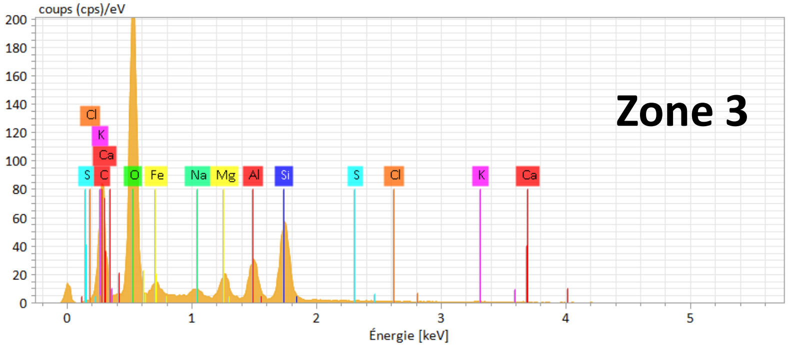

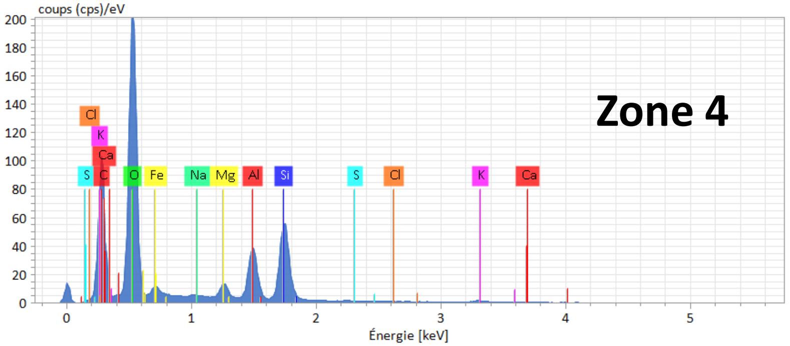

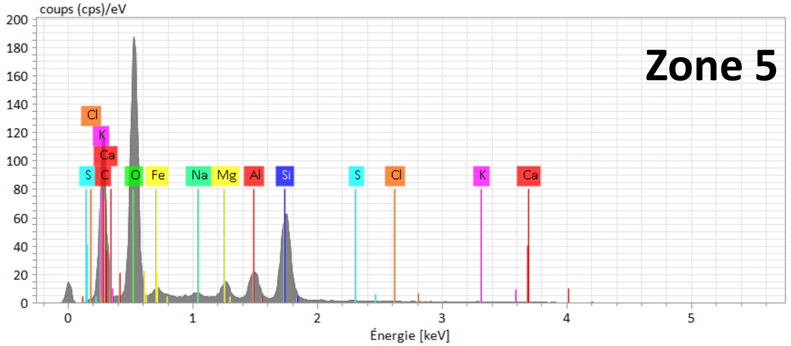

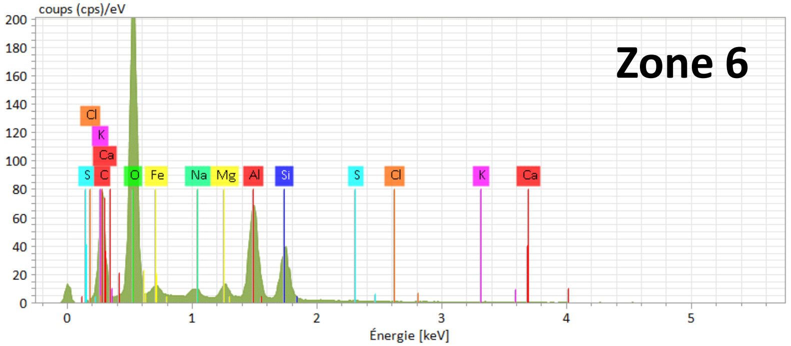

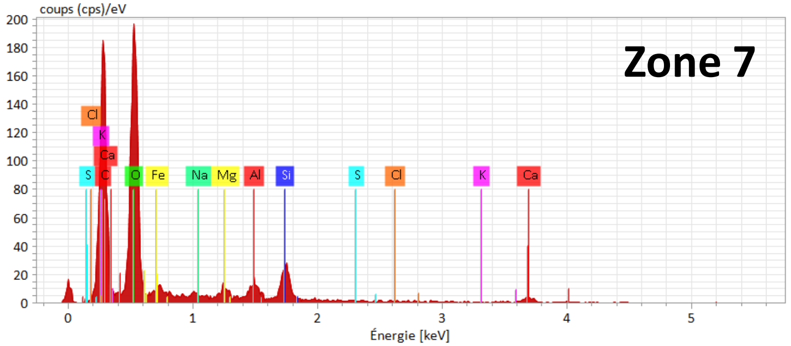

St Vincent ash (SVA) surface species

SVA

Pyroxene particle – **SE images**

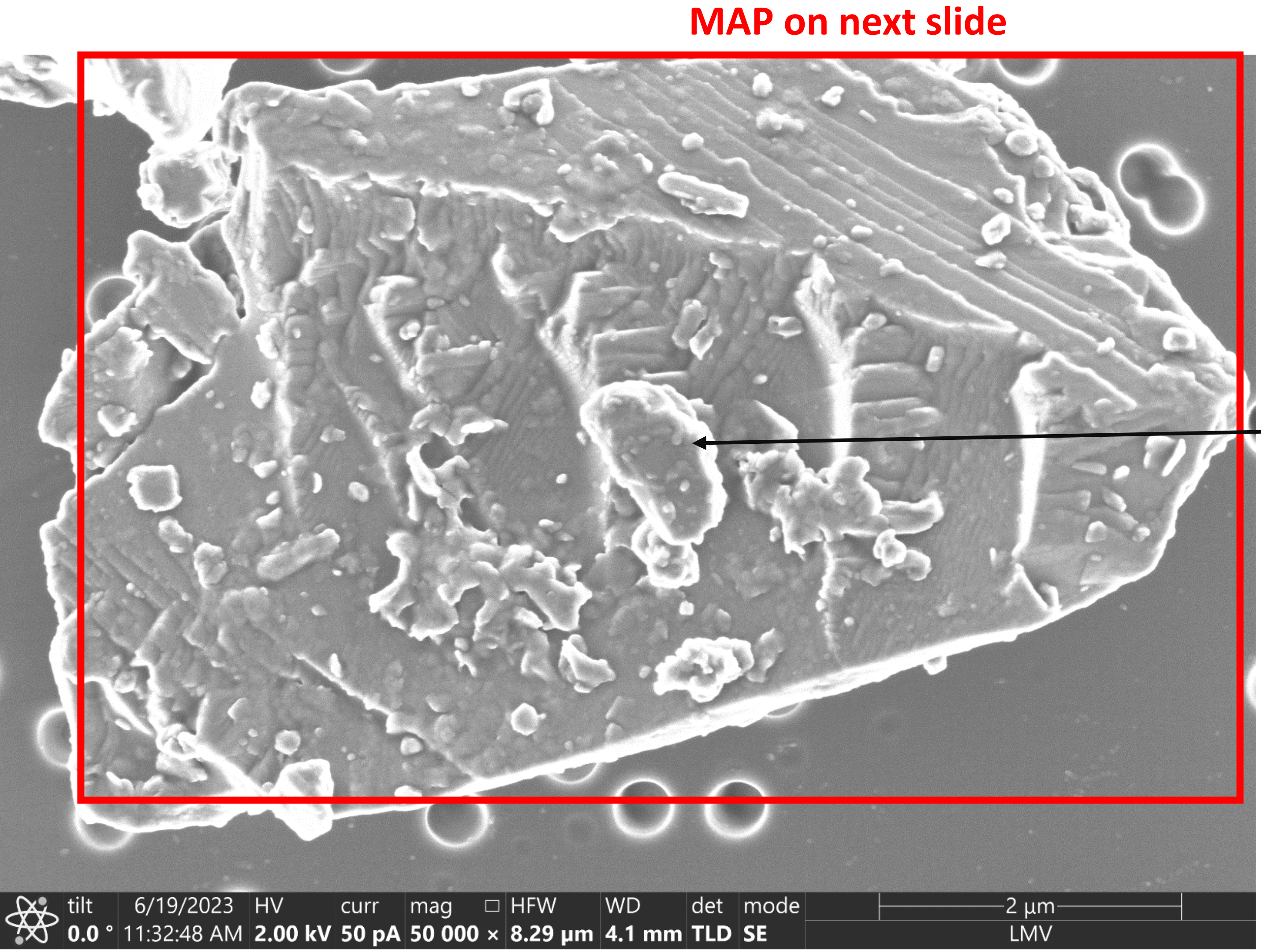

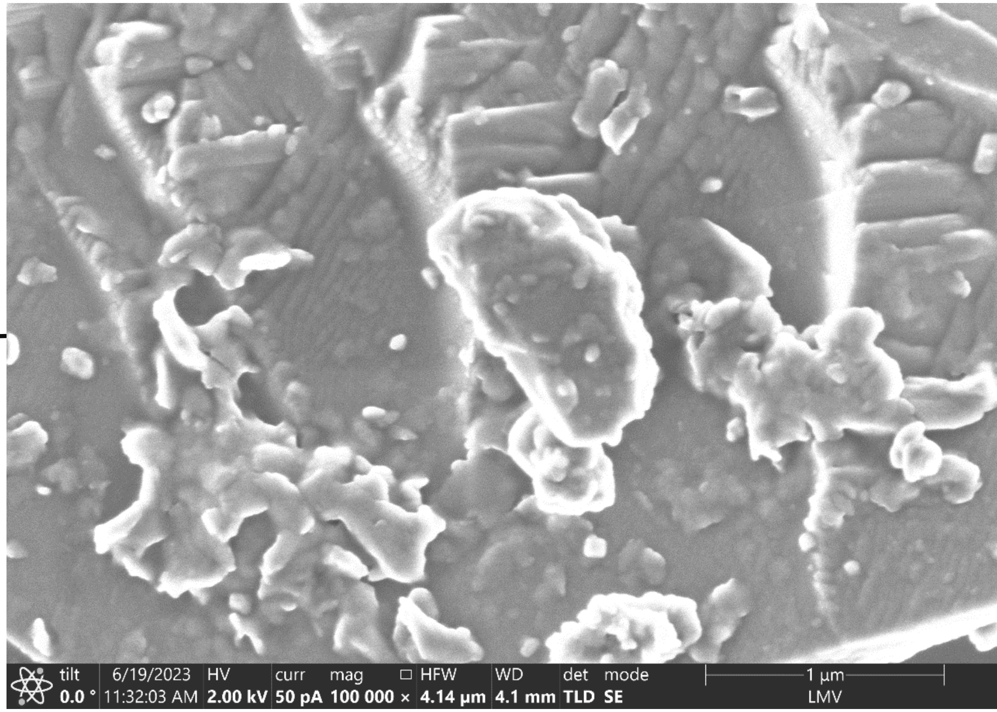

SVA

Pyroxene particle – **EDS elemental MAPS**

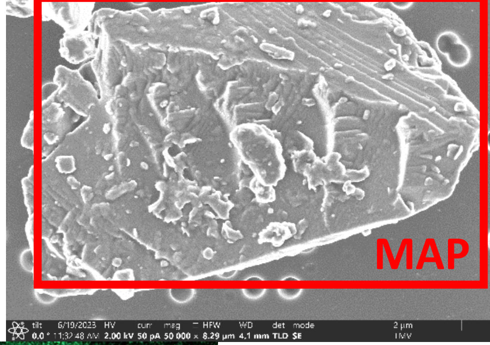

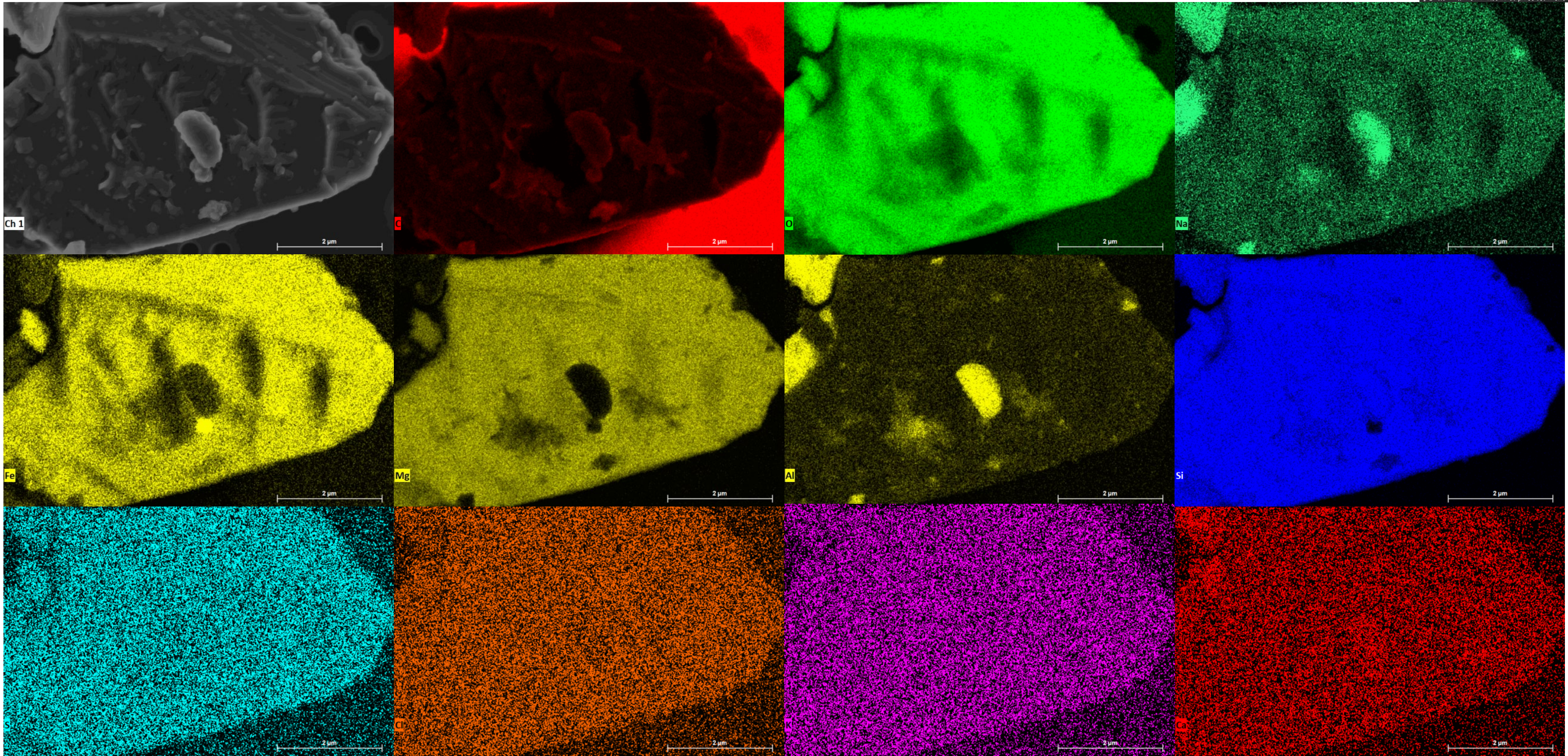

SVA

Pyroxene particle – EDS spectra

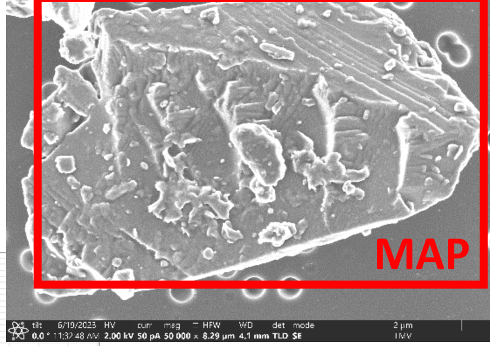

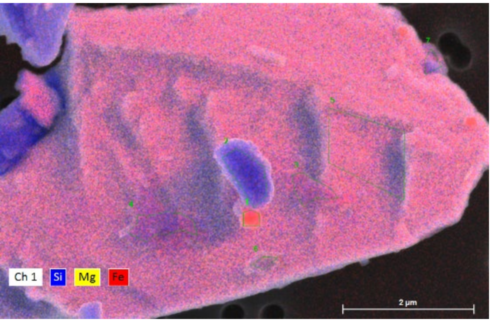

Location on a multi-elemental EDS map of the zones for which the spectra on the right are extracted from.

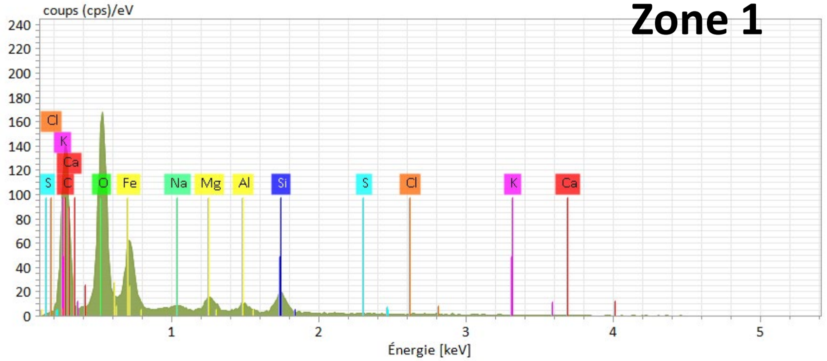

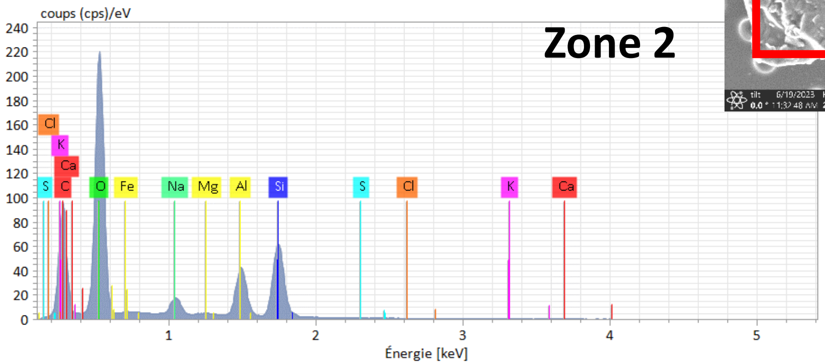

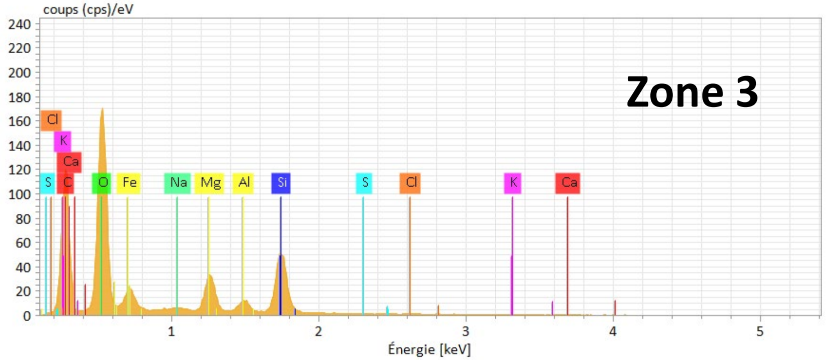

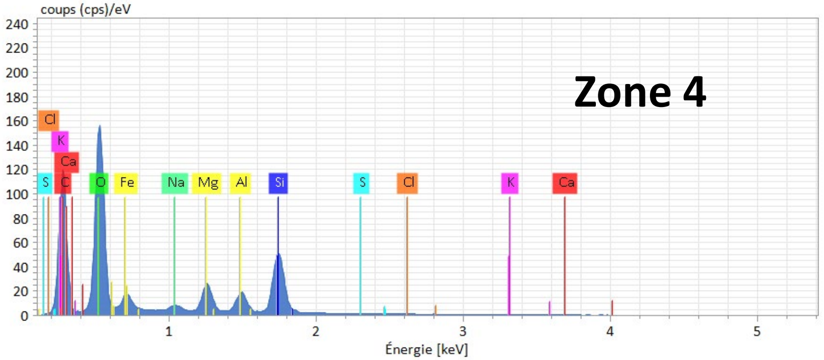

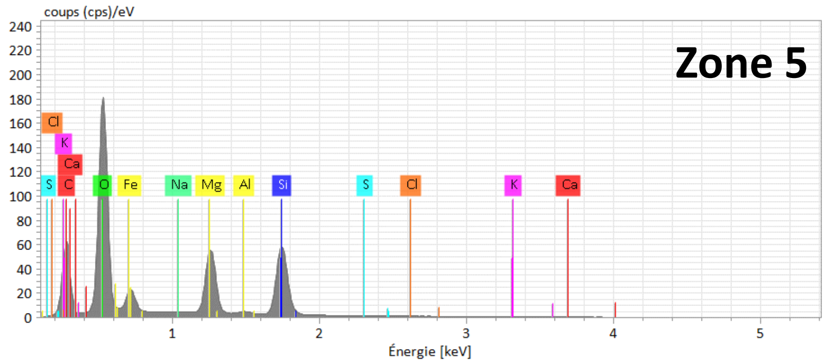

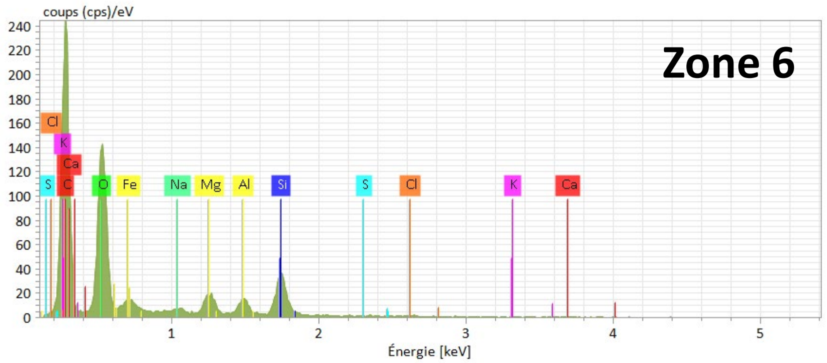

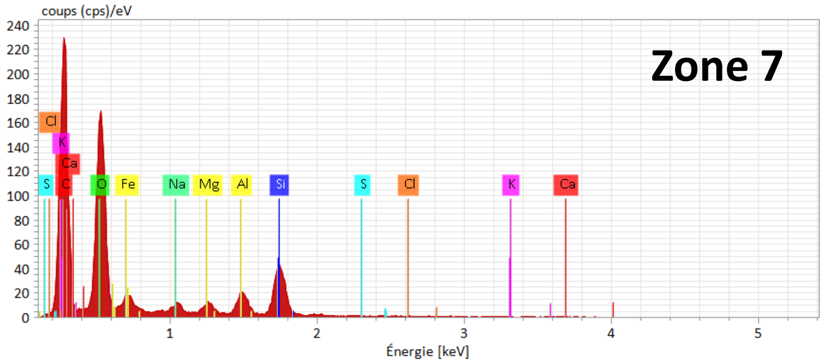

SVA

Glass and crystal aggregate – **SE images**

MAP on next slide

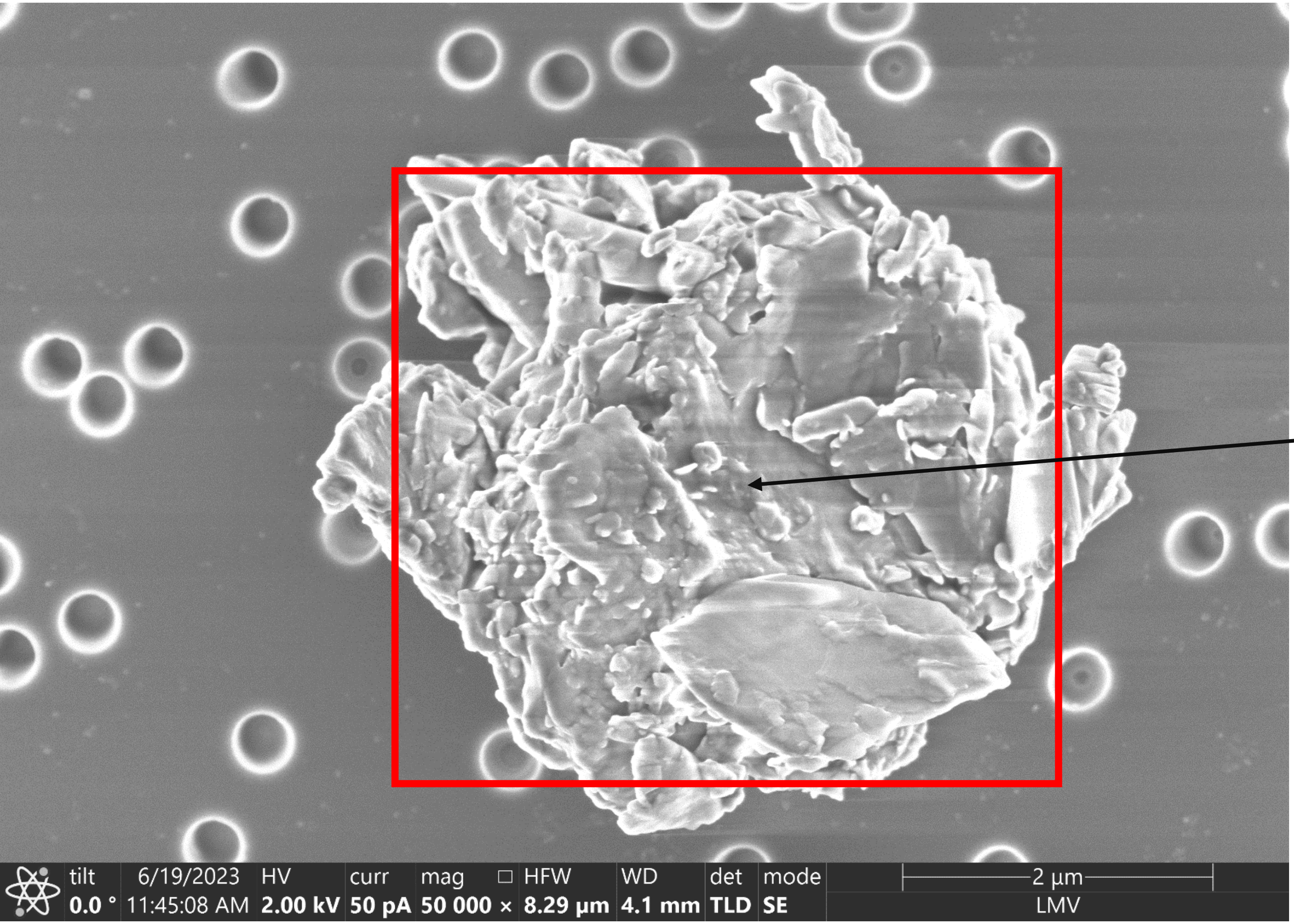

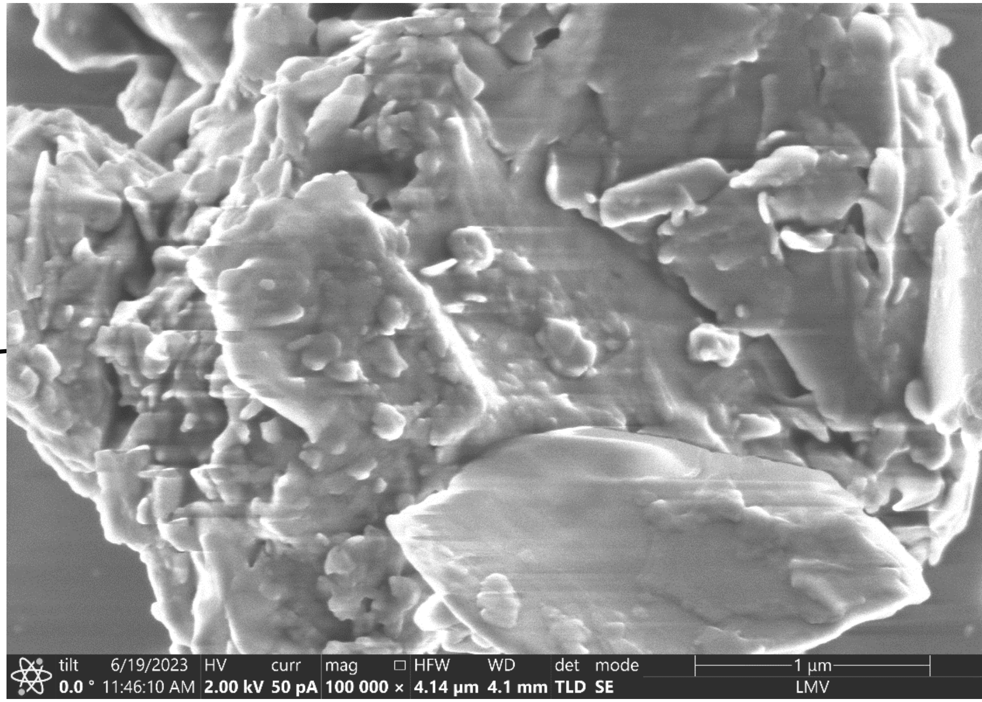

SVA

Glass and crystal aggregate – EDS elemental MAPS

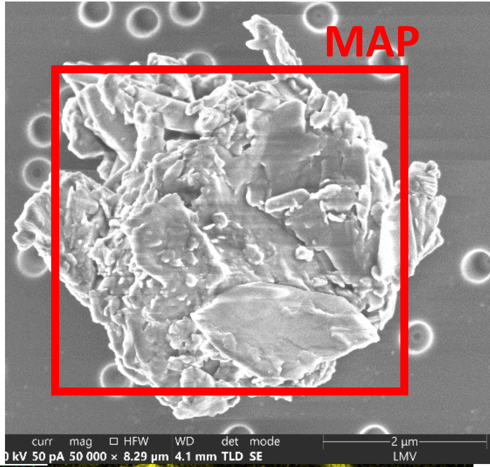

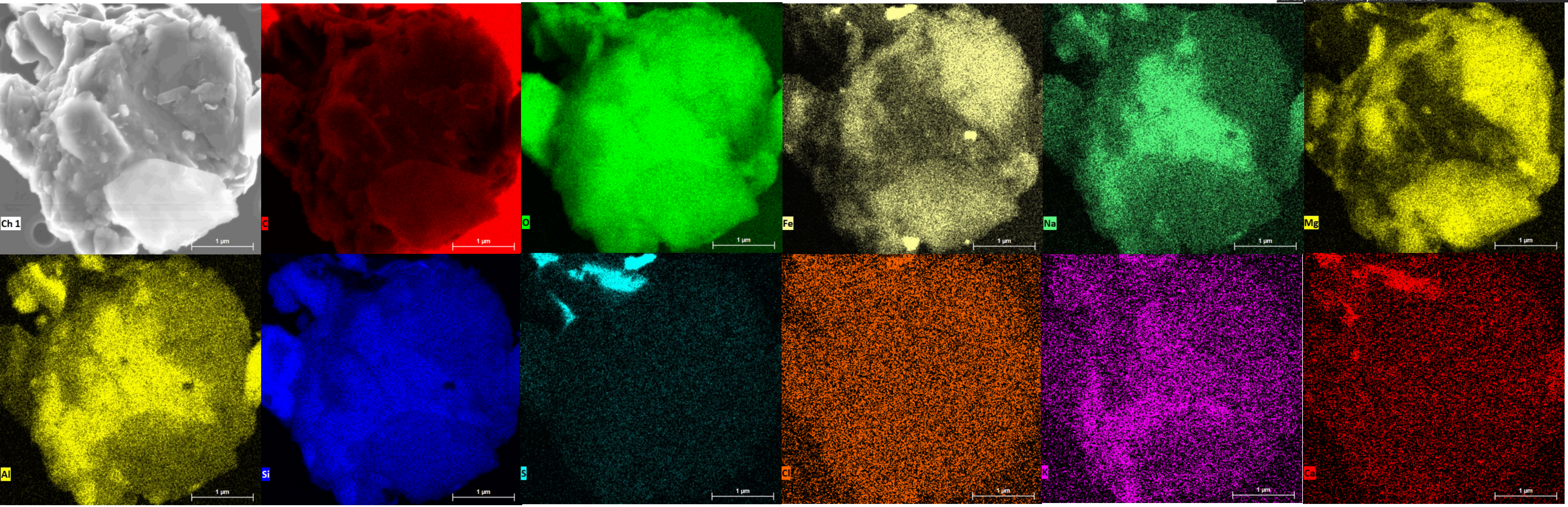

SVA

Glass and crystal aggregate – EDS spectra

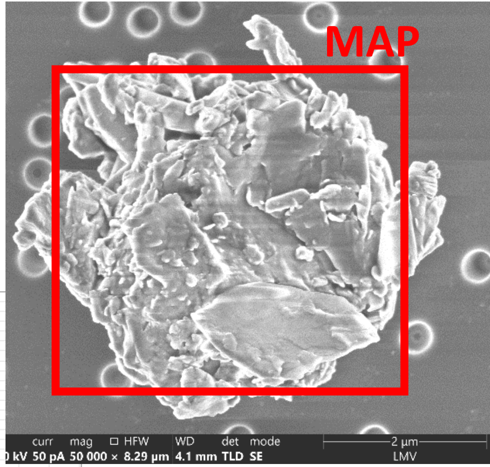

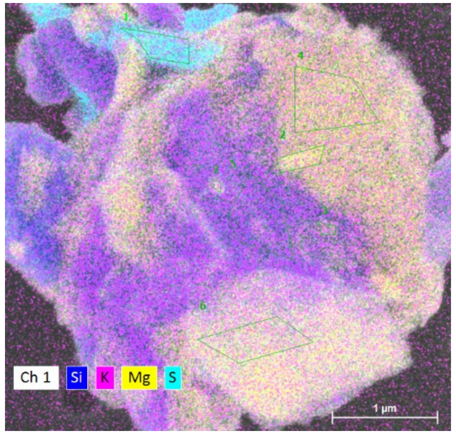

Location on a multi-elemental EDS map of the zones for which the spectra on the right are extracted from.

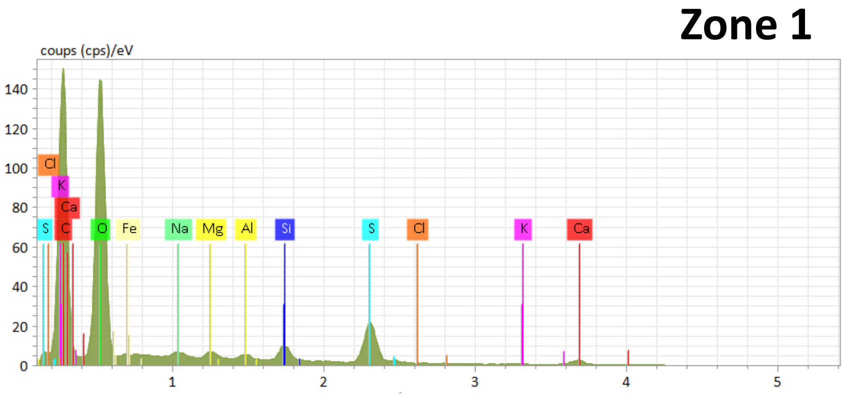

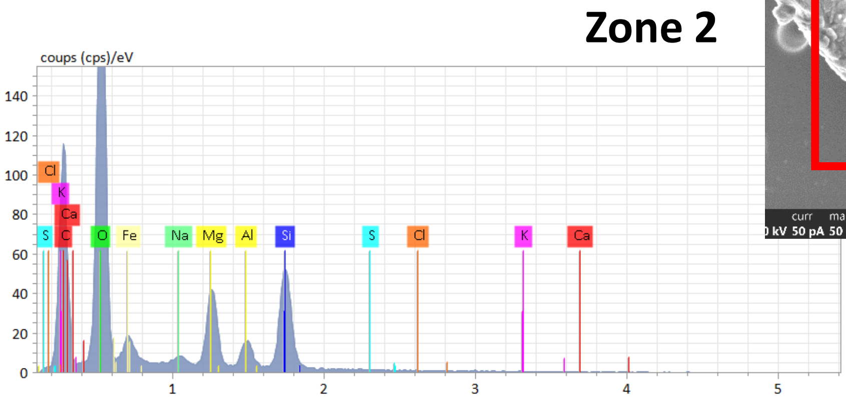

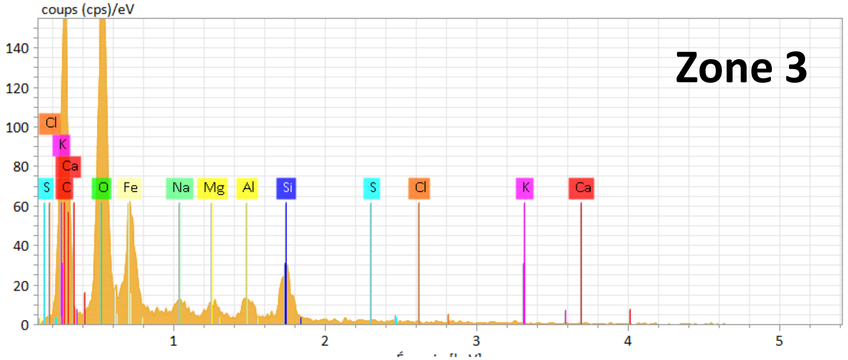

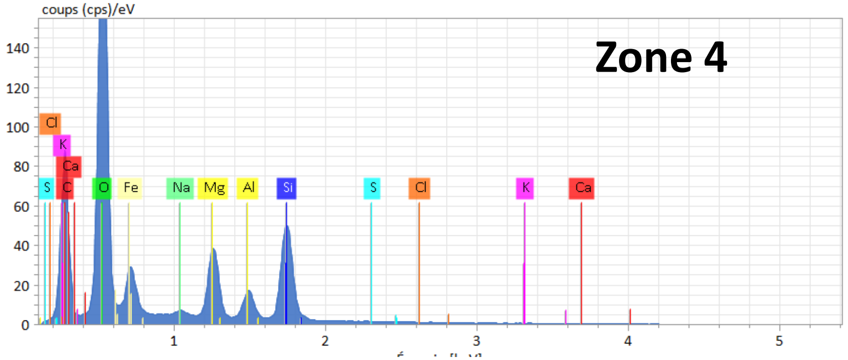

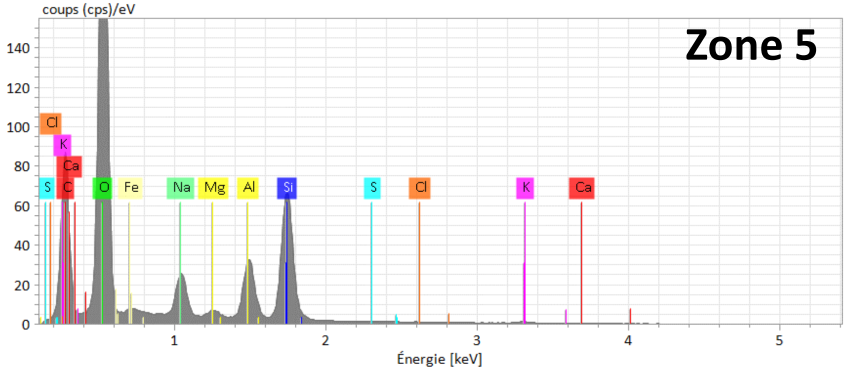

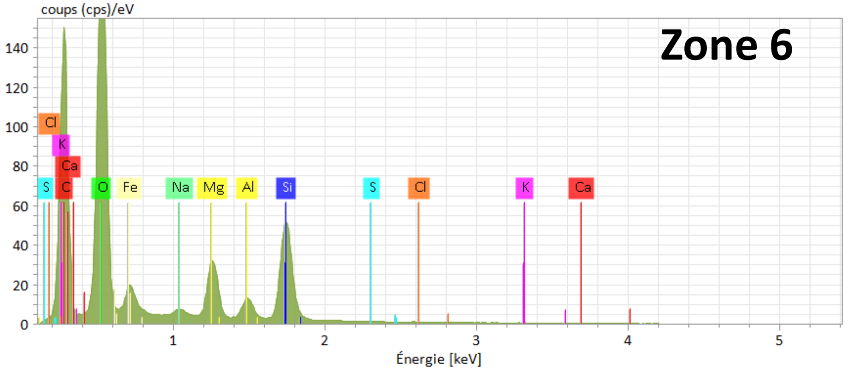

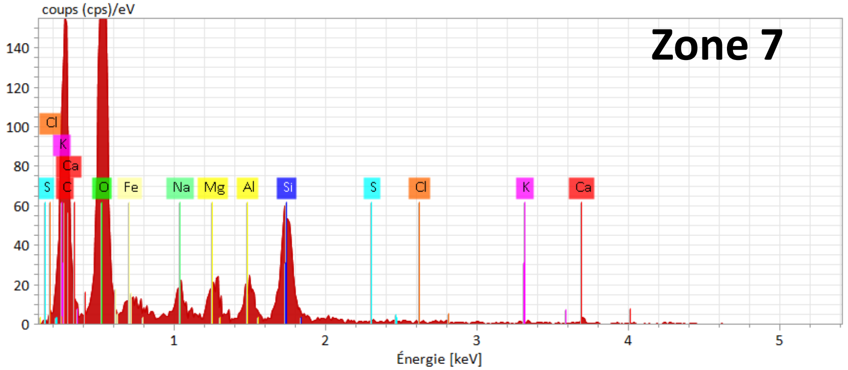

Supplement: Supplementary file 1 — Supporting Information S1 [file GH2-9-e2024GH001171-s001.pdf]
